# Top ten priorities identified by healthcare professionals to support the clinical care of individuals with attention-deficit/hyperactivity disorder: A Canadian Delphi study

**DOI:** 10.1371/journal.pone.0339378

**Published:** 2025-12-19

**Authors:** Ariana Walji, Debra A. Butt, Emma A. Climie, Penny Corkum, Lily Hechtman, Anne-Claude V. Bedard, Yuanyuan Jiang, Natalie Grizenko, Stacey D. Espinet, Carlin J. Miller, Maria A. Rogers, Maggie E. Toplak, Brandy L. Callahan

**Affiliations:** 1 Temerty Faculty of Medicine, Institute of Medical Science, University of Toronto, Toronto, Ontario, Canada; 2 Department of Family and Community Medicine, Scarborough General Hospital, Scarborough Health Network, Scarborough, Ontario, Canada; 3 Department of Family and Community Medicine, Temerty Faculty of Medicine, University of Toronto, Toronto, Ontario, Canada; 4 School & Applied Child Psychology, Werklund School of Education, University of Calgary, Calgary, Alberta, Canada; 5 Department of Psychology, Faculty of Arts, University of Calgary, Calgary, Alberta, Canada; 6 Departments of Psychology and Neuroscience, and Psychiatry, Dalhousie University, Halifax, Nova Scotia, Canada; 7 Child and Adolescent Psychiatry, Montreal Children’s Hospital, McGill University Health Centre, Montreal, Quebec, Canada; 8 Department of Applied Psychology and Human Development, Ontario Institute for Studies in Education, The University of Toronto, Toronto, Ontario, Canada; 9 Faculty of Human Sciences, School of Counselling, Psychotherapy, and Spirituality, Saint Paul University, Ottawa, Ontario, Canada; 10 Faculty of Education, College of Social Sciences and Humanities, University of Alberta, Edmonton, Alberta, Canada; 11 Department of Psychiatry, Douglas Institute, McGill University, Verdun, Quebec, Canada; 12 CADDRA - Canadian ADHD Resource Alliance, Toronto, Ontario, Canada; 13 Department of Psychology, University of Windsor, Windsor, Ontario, Canada; 14 Department of Psychology, Carleton University, Ottawa, Ontario, Canada; 15 Children’s Hospital of Eastern Ontario Research Institute, Ottawa, Ontario, Canada; 16 Department of Psychology, LaMarsh Centre for Child and Youth Research, York University, Toronto, Ontario, Canada; 17 Department of Psychology, Faculty of Arts, University of Calgary, Calgary, Alberta, Canada; 18 Hotchkiss Brain Institute, Calgary, Alberta, Canada; Universitair Kinderziekenhuis Koningin Fabiola: Hopital Universitaire des Enfants Reine Fabiola, BELGIUM

## Abstract

**Background:**

Limited research exists on key issues that healthcare professionals perceive as important for optimizing Attention-Deficit/Hyperactivity Disorder (ADHD) care. This study identified the top ten priorities that healthcare professionals consider vital to support ADHD care in Canada.

**Methods:**

A three-round online Delphi study was conducted using electronic surveys from 2022–2024 across Canada. In Round 1, healthcare professionals were asked to rate 21 predetermined items using a 5-point Likert scale and re-evaluated those rankings in Round 2. In Round 2, a new set of 34 items identified from Round 1 were rated and the rankings re-evaluated in Round 3. Consensus was determined by percentage agreement ≥ 90% with a Likert score ≥ 4. For each priority item, the mean Likert score, standard deviation, 95% confidence interval (CI), and the minimum and maximum Likert scores were calculated.

**Results:**

96 Canadian healthcare professionals completed Round 1. 82 (85% response rate) completed Round 2 and 73 (89% response rate) completed Round 3. The two highest ranked priorities that achieved 100% consensus agreement were: providing access to well-trained healthcare providers in ADHD (mean score 4.74, 95% CI 4.65–4.84) and access to ADHD-related services (mean score 4.50, 95% CI 4.39–4.61). Among the top ten consensus-derived items, the highest frequency pertained to providing access to healthcare experts in ADHD and related-services (50%) followed by research into ADHD on socio-emotional functioning, co-existing conditions and in diagnosing ADHD in females (30%). Increasing knowledge and educating healthcare professionals and school systems on ADHD was also identified among the top ten priorities (20%).

**Conclusions:**

Healthcare professionals identified ten top priorities by consensus where most focused on providing access to trained healthcare providers and services to support ADHD care in Canada. Implementing strategies to improve access on a national level will improve the quality of life for individuals living with ADHD.

## Introduction

Attention-Deficit/Hyperactivity Disorder (ADHD) is a chronic neurodevelopmental condition that starts in childhood and is characterized by symptoms of inattention, hyperactivity and/or impulsiveness [1]. Individuals with ADHD experience significantly more impairments related to academic, social, occupational, and mental functions compared to those without ADHD [1,2]. Although some ADHD symptoms (e.g., hyperactivity) tend to decrease with age, others (e.g., attentional problems) often persist and cause significant difficulties. At least 50% of children with ADHD experience symptoms that may continue to persist into adulthood [3,4]. ADHD is commonly associated with other psychiatric conditions [5,6], and individuals with ADHD are also at risk of accidents, injuries, and even premature death [2,7]. Early detection, diagnosis, and treatment of ADHD in the Canadian healthcare system is of critical importance in helping to alleviate the adverse consequences associated with this condition.

However, recent evidence indicates that Canadian healthcare professionals are struggling to meet the growing demands of individuals with ADHD [8–10]. The Canadian healthcare system is currently facing a shortage of healthcare professionals commonly involved in the clinical care of individuals with ADHD such as specialists, family physicians and publicly funded psychologists, and this shortage has contributed to a significant gap in access to equitable ADHD care [8,11,12]. In 2024, approximately 6.5 million people living in Canada were without a family physician [13]. Moreover, the Ontario Medical Association estimates a 15% decline in psychiatrists by the year 2030 [14]. In a survey of 1600 pediatricians affiliated with the American Academy of Pediatrics, over 90% agreed they should be responsible for diagnosing ADHD in children and youth, and 70% thought they should treat or manage ADHD [15]. Yet, pediatricians are failing to provide adequate monitoring of their patients with ADHD over time after initial diagnoses and treatment [16]. A study involving children and youth with ADHD that were prescribed medication or receiving psychosocial treatment found that less than half (47.4%) received a follow-up visit within a month from their pediatrician, and parent- and teacher-ratings were rarely collected contrary to national guidelines to do so [16]. Although national ADHD guidelines have been in place for over a decade, the majority of primary care physicians and specialists have not been successful in fully implementing them into practice [16–20]. These shortages in the physician workforce in Canada contribute to a growing healthcare crisis, as it reduces the pool of physicians available to provide clinical care and prescribe medications to treat ADHD.

In addition, there is an urgent need for more psychologists to provide ADHD diagnosis and management in Canada. An online survey conducted by Nanos in 2020 indicated that 75% of Canadians aged 18 years and older (n = 3070) felt that a significant barrier to accessing care was the lack of mental health services to a psychologist [21]. In fact, most clinical care offered by psychologists is not covered by the Canadian healthcare system, creating health inequities and unmet healthcare needs in the ADHD population among those with private coverage under their employment health benefits versus those without [10,22–24]. In another online survey conducted by Nanos in 2020 involving 657 Ontario residents, about 80% favoured psychologists working collaboratively with other healthcare professionals like family physicians in a primary care team to address their mental healthcare needs [21].

In light of these shortages among healthcare professionals, Canadians have more difficulty finding healthcare services and obtaining treatment for ADHD compared to other mental health conditions such as anxiety and depression [10]. Population health data have demonstrated an increase in both prevalence and incidence rates of diagnosed ADHD in children and youth aged 1−24 years in the province of Ontario from 2014−2021 [25]. As a result, primary care physicians in Ontario are increasingly being sought by individuals with ADHD-related concerns to provide healthcare services and the use of these services substantially increased by 32% during the COVID-19 pandemic from 2020−2021, compared to the pre-pandemic period from 2017−2019 [9]. Furthermore, the COVID-19 pandemic was associated with a global rise in ADHD symptoms, which is expected to continue in the upcoming years [26]. Given these circumstances, it is imperative that we understand the current challenges and/or barriers that healthcare professionals are facing in their ability to deliver clinical services amidst a rising demand for ADHD care.

There is a paucity of research published on the perspectives of healthcare professionals who provide clinical care to individuals with ADHD, particularly in identifying issues that merit critical attention. To develop a comprehensive understanding of the key priorities that Canadian healthcare professionals perceive as important in the clinical care of individuals with ADHD, it is necessary to conduct qualitative research, a method that is often overlooked in ADHD research [27,28]. Specifically, the Delphi methodology, which dates back to the 1950s, generates consensus-based data using a multistage survey approach among a panel of experts within a designated field [29,30]. The technique is well-recognized and commended for applying anonymity when gathering data and offering structured feedback to participants [30–32]. The aim of this study is to use the Delphi method to achieve consensus on the top ten priorities that healthcare professionals consider clinically important to support ADHD care in Canada. The findings from this study will raise awareness of the major issues that need to be addressed in the Canadian healthcare system to tackle the unmet healthcare needs of individuals with ADHD and consequently, assist healthcare professionals in optimizing the quality of care provided to those with ADHD.

## Methods

### Study design

This study used the Delphi method to collect opinions and reach agreement on key issues that Canadian healthcare professionals perceive as important in the clinical care of individuals with ADHD. The Delphi methodology has been widely applied in health sciences research and is best suited for gathering individual opinions across a broad geographical area like Canada from a panel of experts referred to as panelists, to generate group consensus and formulate high priority issues [29–34]. This study uses essential features of the classic Delphi method to ensure good quality design measures, including specific sampling and anonymity of panelists, iterative Delphi rounds, controlled feedback, and consensus criteria [31–34]. The study also follows Delphi reporting guidelines in DELPHI studies in social and health sciences- recommendations for a STAndardized Reporting (DELPHISTAR) [35] and consensus guidelines from the ACcurate COnsensus Reporting Document (ACCORD) [36]. These completed checklists can be found in the Supporting information (S1 and S2 Checklists). Also, this study protocol was not registered on any platform.

The study was approved by the Institutional Review Board of the University of Calgary (REB 22−0011, approved April 4, 2022). Informed consent was obtained from all subjects involved in the study.

### Panelist recruitment and selection

Subjects who participated in this Delphi study were recruited from November 22, 2022 to February 7, 2023 via electronically distributed surveys after providing online informed consent to participate in the study. The surveys were distributed through social media platforms and via email to the members of two national organizations in ADHD: Canadian ADHD Resource Alliance (CADDRA) and Centre for ADHD Awareness Canada (CADDAC). Surveys were also distributed by some study authors to their professional organizations. Participants had the option of taking the surveys in English or French (the two official languages of Canada) (S1 Survey).

This study is one component of a larger Delphi study that involved a total of four different stakeholder groups collectively referred to as the survey participants, which consisted of healthcare professionals, educators, researchers, and people with lived experiences related to ADHD. The current study only evaluates the responses of the healthcare professional stakeholder group, consisting of participants who self-identified their primary role as a “clinical practitioner serving a clientele with ADHD” or as “students or trainees with plans to continue as a clinician” in Round 1 of the survey. Therefore, the panelists for this Delphi study are defined as Canadian healthcare professionals.

### Procedure

Each survey round was distributed via e-mail to the panelists using Qualtrics, an online research platform. The survey priority items were rated using a 5-point Likert scale from 1 (not a priority), 2 (low priority), 3 (undecided), 4 (high priority) to 5 (critical priority). Responses to the Delphi rounds were kept anonymous (by following a direct online link provided by email to the individual recipient) to allow for honest and unbiased responses and participant personal information was de-identified prior to conducting analyses.

#### Round 1.

**Fig 1:**
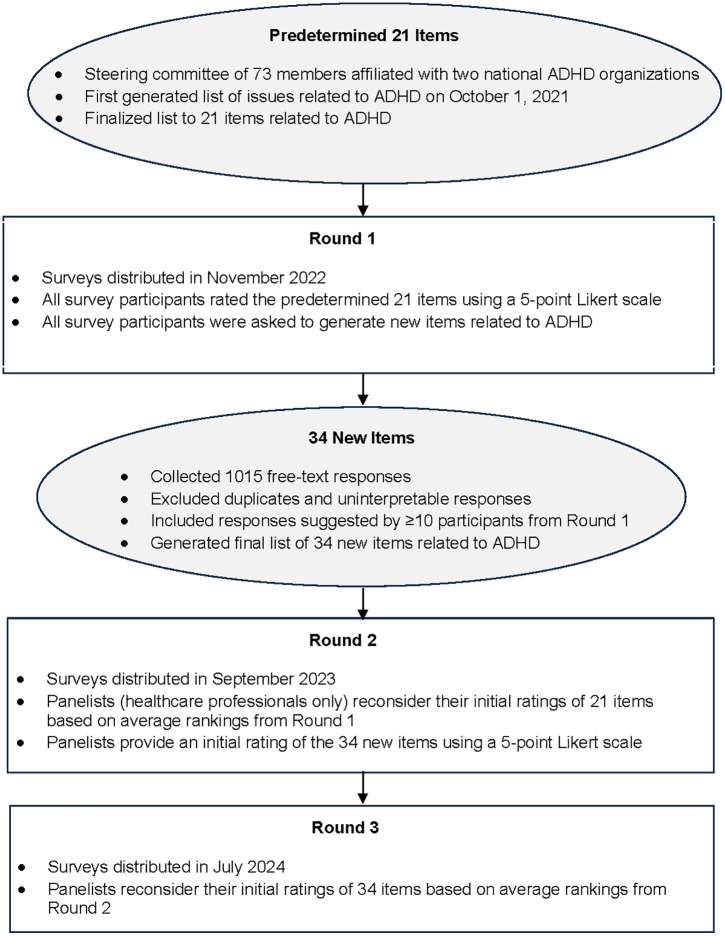
Overview of the Delphi method. ADHD = Attention-Deficit/Hyperactivity Disorder; Survey participants = all the participants who responded to the survey which included healthcare professionals.

The first survey served three purposes: i) to collect the initial ratings of the 21 predetermined items, ii) to collect suggestions for additional issues related to ADHD, and iii) to identify the primary role of the survey participants in order to distribute role-specific surveys in subsequent rounds to the panelists (healthcare professionals). All participants in Round 1 received the same survey. All survey participants were asked some general demographic questions pertaining to gender, age category, province and clinical discipline, and to provide a rating of 21 items related to ADHD using a 5-point Likert scale. A list of ADHD-related priorities was initially created by a steering committee consisting of 73 experts in ADHD representing physicians, psychologists, allied health care professionals and researchers who came together at the 8^th^ Annual CADDRA ADHD Research Day. This list was then shortlisted to 21 items. A complete list of the 21 predetermined items can be found in the Supporting information (S1 Table). Fig 1 outlines the flow of the Delphi study.

Survey participants were also prompted to provide open-ended suggestions of additional items that were not captured by the 21 predetermined items related to ADHD. Data collection remained open for 11 weeks from November 22, 2022 until February 7, 2023, after releasing the initial email invitation. One reminder email was sent two weeks prior to closing Round 1. Participants in this round were given the chance to win one of 10, $50 gift cards to encourage participation.

A total of 1015 free-text responses were received from Round 1 (Fig 1). For details regarding the derivation of the 34 new items, see S1 Appendix.

#### Round 2.

The second survey was distributed online on September 12, 2023 to the panelists from Round 1 who consented to participate in subsequent rounds and was available until November 2, 2023. Panelists were provided with a graphical representation of the average rankings for each of the 21 items from Round 1. Based on this summary, panelists were asked to reconsider their initial ratings of the 21 items using the 5-point Likert scale. Panelists were also shown the 34 new items generated from Round 1 and were asked to provide their initial ratings using the 5-point Likert scale. Multiple e-mail reminders were sent during Round 2. Participants in Round 2 were given the chance to win 1 of 10, $50 gift cards to encourage participation.

#### Round 3.

The third survey was distributed online to panelists from July 10, 2024 to August 22, 2024. Additional demographic data were included in the third survey such as ethnic background, practice setting, job status, household income, location of clinical specialty training, years of experience, insured versus uninsured services offered, and types of clinical assessments. Panelists were provided with graphical representations of the average rankings for each of the 34 new items from Round 2. Based on this summary, panelists were asked to reconsider their initial ratings of the 34 items using the 5-point Likert scale. Panelists were sent several e-mail reminders throughout the data collection period. Participants in this round were given the chance to win one of 10, $50 gift cards to encourage participation.

### Statistical analysis

Counts and percentages were used to describe the sociodemographic characteristics of the healthcare professionals who participated in the Delphi study. Consensus, an objective measure for observing the level of agreement among the panelists [33], was determined as ≥ 90% of healthcare professionals in agreement that a particular item represented a high or critical priority area (designated by a Likert score of 4 or 5), and this was defined as percentage agreement. The percentage agreement was calculated by adding the number of healthcare professionals who rated a specific item as 4 or 5, divided by the total number of healthcare professionals who provided a response for that item, multiplied by 100. We calculated consensus after two rounds of the same survey items. This occurred in Round 2 for the 21 predetermined items and again in Round 3 for the 34 new items. For each item, the mean Likert score, standard deviation, 95% confidence interval (CI), and the minimum and maximum Likert scores were calculated. After consensus was achieved, we ranked the issues from highest to lowest priority based on the highest percentage agreement and in cases where the percentage agreement was identical, the order was determined by the highest mean Likert score. Priority outcomes based on consensus in Round 2 for the 21 predetermined items, and for the 34 items in Round 3 were presented separately similar to other published studies using the Delphi method [33–37]. The top ten priority items from Round 2 and Round 3 were then ranked in order of importance from 1 to 10. A robustness check using Kendall’s W was done using different consensus thresholds from 70–80% to ensure stability of the top ten priority rankings. We also performed a post hoc analysis examining the top ten priorities among specific groups of healthcare professionals. All statistical analyses were performed using Python software (version 3.10.11) and libraries such as Pandas, NumPy, and SciPy for data manipulation.

## Results

### Demographic characteristics of the panelists

There were 96 panelists who self-identified their primary role in ADHD care as being a healthcare professional in Round 1. Of these panelists, 82 (85%) proceeded to Round 2. In Round 3, there were 76 healthcare professionals but three were excluded because they did not provide ratings for any of the 34 priority items, leaving 73 (89%) who were retained as the final number of panelists. Common characteristics of the panelists who completed all three Rounds are described in the Supporting information (S2 Table).

Table 1 describes more detailed sociodemographic characteristics of the healthcare professionals who completed Round 3. The majority of the Round 3 respondents were female (79.5%) and in the age group 40–59 years (46.6%). The largest reported ethnicity among healthcare professionals was Caucasian/European/White (87.7%). Most healthcare professionals reported residing in Ontario (28.8%) and working in an urban area (87.7%). The majority of healthcare professionals self-identified as physicians (psychiatrists, family physicians, and pediatricians) who represented 32.9% of the sample, followed by psychologists (24.7%), psychotherapists (19.2%), and nurses (4.1%). Other healthcare professionals (6.8%) included social workers (2), a school counsellor, an ADHD coach, and an occupational therapist. The highest proportion of healthcare professionals were employed full-time (46.6%) and earned a household income greater than $200,000 Canadian dollars (35.6%). Most graduated in Canada (84.9%) and reported having over 20 years of clinical experience (45.2%). In terms of health services provided, most healthcare professionals reported offering both private and insured health services (50.7%), as well as a combination of virtual and in-person assessments (56.2%) (Table 1).

**Table 1:** Demographic characteristics of healthcare professionals (N = 73).

Characteristics	No. (%)	
**Gender**		
Female	58	(79.5)
Male	12	(16.4)
Other	2	(2.7)
Prefer not to say	1	(1.4)
**Age Category (years)**		
25-39	16	(21.9)
40-59	34	(46.6)
60+	23	(31.5)
**Ethnic Background** ^a^		
Caucasian/European/White	64	(87.7)
Asian (e.g., Chinese, Korean, Japanese)	4	(5.5)
Black/African	3	(4.1)
Middle Eastern/Arab/West Asian (e.g., Egyptian, Kuwaiti, Iranian)	3	(4.1)
South Asian/East Indian (e.g., Pakistani, Sri Lankan)	3	(4.1)
Metis	3	(4.1)
Latin American/Hispanic (e.g., Mexican, Chilean)	2	(2.7)
Filipino/Pacific Islander	2	(2.7)
First Nations/Aboriginal	1	(1.4)
Prefer not to say	1	(1.4)
Other	1	(1.4)
Inuit/Inuk	0	(0.0)
**Province**		
Alberta	14	(19.2)
British Columbia	10	(13.7)
Manitoba	1	(1.4)
New Brunswick	2	(2.7)
Newfoundland and Labrador	6	(8.2)
Nova Scotia	1	(1.4)
Ontario	21	(28.8)
Prince Edward Island	2	(2.7)
Quebec	13	(17.8)
Saskatchewan	3	(4.1)
**Practice Setting**		
Urban	64	(87.7)
Rural	9	(12.3)
**Clinical Discipline**		
Adult Psychiatry	4	(5.5)
Adult Psychology	10	(13.7
Child & Adolescent Psychiatry	3	(4.1)
Child & Adolescent Psychology	8	(11)
Psychotherapy	14	(19.2)
Family Medicine	10	(13.7)
Pediatrics	7	(9.6)
Nursing	3	(4.1)
Other	5	(6.8)
Missing	9	(12.3)
**Job Status**		
Employed full time	34	(46.6)
Employed part-time	8	(11.0)
Self-employed	28	(38.4)
Student	1	(1.4)
Other	2	(2.7)
**Household Income**		
<$25K	2	(2.7)
$25K–$50K	3	(4.1)
$50K–$75K	2	(2.7)
$75K–$100K	4	(5.5)
$100K–$150K	21	(28.8)
$150K–$200K	12	(16.4)
> $200K	26	(35.6)
Missing	3	(4.1)
**Location of Clinical Specialty Training**		
Graduated in Canada	62	(84.9)
Graduated outside Canada	11	(15.1)
**Years of Experience**		
<5 years	11	(15.1)
5–10 years	13	(17.8)
11–20 years	16	(21.9)
>20 years	33	(45.2)
**Services Offered**		
Insured only	26	(35.6)
Private only	10	(13.7)
Both	37	(50.7)
**Type of Clinical Assessment**		
In-person only	17	(23.3)
Virtual only	10	(13.7)
Both	41	(56.2)
Missing	5	(6.8)

^a^Panelists were asked to select all that apply so may not add up to 100% given the multiple responses.

#### Round 2.

The healthcare professionals reached consensus on six out of the 21 predetermined items (Table 2). A complete list of the rankings for the 21 items can be found in the Supporting information (S3 Table). The two highest ranked issues that achieved 100% agreement were: providing access to well-trained healthcare providers in ADHD (mean score 4.74, 95% CI 4.65–4.84) and access to specific ADHD services (mean score 4.50, 95% CI 4.39–4.61). The dominant theme that emerged was issues related to access for individuals with ADHD. The subsequent items ranked from 3–4 both pertained to issues related to the need for research on co-morbidities in ADHD and diagnosing ADHD in females, which both reached 96% agreement. The last two priority items ranked from 5–6 were related to increasing knowledge about ADHD among educators and parents, which both reached 94% agreement.

**Table 2:** Priority items identified by healthcare professionals in Round 2 (n = 82).

Order	Item	Percentage Agreement (%)	Mean	95% CI	SD	Median	IQR	Min	Max	N
1	Providing access to healthcare providers who are well-trained to recognize ADHD	100.00	4.74	4.65-4.84	0.44	5.00	0.75	4.00	5.00	82
2	Providing access to ADHD services (e.g., CBT, coaching, skills-based training, employment programs)	100.00	4.50	4.39-4.61	0.50	4.50	1.00	4.00	5.00	82
3	Research on how co-existing experiences (e.g., depression, anxiety) should be considered when diagnosing ADHD	96.30	4.48	4.35-4.61	0.57	5.00	1.00	3.00	5.00	81
4	Research on diagnosing ADHD in girls and women	96.30	4.44	4.32-4.57	0.57	4.00	1.00	3.00	5.00	81
5	Increasing knowledge about ADHD among teachers and educators	93.90	4.54	4.39-4.68	0.65	5.00	1.00	2.00	5.00	82
6	Increasing knowledge about ADHD among parents	93.90	4.34	4.20-4.48	0.63	4.00	1.00	2.00	5.00	82

ADHD = Attention-Deficit/Hyperactivity Disorder; CBT = Cognitive Behavioural Therapy; CI = Confidence Interval; IQR = Interquartile Range; Max = maximum Likert score; Mean = mean Likert score; Median = median Likert score; Min = minimum Likert score; N = number of healthcare professionals that responded; SD = Standard Deviation.

#### Round 3.

The healthcare professionals reached consensus on 13 out of the 34 priority items (Table 3). A list of the rankings of all 34 items can be found in the Supporting information (S4 Table). The highest percentage agreement (98.5%) was achieved for research on socio-emotional functioning in ADHD (mean score 4.50, 95% CI 4.37–4.63) followed by increasing knowledge and training about ADHD and associated stigmas among healthcare providers, as well as providing access to funded services for individuals with ADHD and their loved ones, which both had a percentage agreement of 97.3%. Common themes that emerged from these high priority items were providing access to funded healthcare services for ADHD (items 3, 5, 6); the need for research into specific areas related to ADHD (items 1, 8, 11); increasing knowledge and training related to ADHD (items 2, 10, 12); the assessment of ADHD (items 7,9); and the education of ADHD to certain groups (items 4, 13).

**Table 3:** Priority items identified by healthcare professionals in Round 3 (n = 73).

Order	Item	Percentage Agreement (%)	Mean	95% CI	SD	Median	IQR	Min	Max	N
1	Research on socio-emotional functioning in ADHD (e.g., self-esteem issues, ability to regulate emotions) and its impact on relationships	98.50	4.50	4.37-4.63	0.53	5.00	1.00	3.00	5.00	68
2	Increasing knowledge and training about ADHD and associated stigmas among all healthcare and mental health professionals (e.g., family doctors, nurse practitioners, pharmacists, psychologists, counsellors)	97.30	4.71	4.58-4.84	0.56	5.00	0.00	2.00	5.00	73
3	Providing access to funded services for individuals with ADHD and their loved ones (e.g., healthcare coverage for psychological services, and/or affordable options)	97.30	4.59	4.46-4.72	0.55	5.00	1.00	3.00	5.00	73
4	Educating personnel in the school systems on how to best support and teach individuals with ADHD	97.20	4.72	4.60-4.84	0.51	5.00	0.50	3.00	5.00	71
5	Providing access to resources and services to smaller and/or rural communities	95.90	4.41	4.28-4.54	0.57	4.00	1.00	3.00	5.00	73
6	Providing more accessible information and support to navigate the healthcare system and find appropriate services/personnel to assist and advocate for individuals with ADHD	95.90	4.37	4.24-4.50	0.57	4.00	1.00	3.00	5.00	73
7	Optimizing the assessment process through the use of validated tools to improve early diagnosis and diagnostic accuracy, and reduce misdiagnosis	93.20	4.45	4.31-4.60	0.62	5.00	1.00	3.00	5.00	73
8	Research on best treatments for addictions within the context of ADHD (e.g., substances, gaming, gambling, and screens)	92.40	4.48	4.33-4.64	0.64	5.00	1.00	3.00	5.00	66
9	Identifying delays/barriers to assessment and treatment, and the impacts they may have on different systems	91.80	4.49	4.34-4.64	0.65	5.00	1.00	3.00	5.00	73
10	Increasing general awareness of ADHD and its impacts in girls and women (e.g., among healthcare providers, across the lifespan, education, workplace)	91.20	4.31	4.12-4.50	0.80	4.00	1.00	2.00	5.00	68
11	Research on recognizing and diagnosing ADHD in mid-life (35–50)	91.20	4.12	3.96-4.27	0.64	4.00	0.00	2.00	5.00	68
12	Increasing understanding of ADHD as a condition warranting recognition by government and educational systems	90.80	4.48	4.31-4.64	0.66	5.00	1.00	3.00	5.00	65
13	Providing individuals with ADHD with the tools, information, and strategies to self-advocate	90.40	4.21	4.05-4.37	0.69	4.00	1.00	2.00	5.00	73

ADHD = Attention-Deficit/Hyperactivity Disorder; CI = Confidence Interval; IQR = Interquartile Range; Max = maximum Likert score; Mean = mean Likert score; Median = median Likert score; Min = minimum Likert score; N = number of healthcare professionals that responded; SD = Standard Deviation.

### Top ten priorities

Table 4 summarizes the top ten priority items that were identified by healthcare professionals following the consensus process (Table 2 and Table 3). The most frequently endorsed priority items appeared to be related to providing access to healthcare experts in ADHD and specific ADHD-related services (50%: items 1,2,5, 9,10). The other high priority items were related to research into ADHD (30%: items 3,7,8), followed by issues relating to increasing knowledge and educating different groups about ADHD (20%: items 4 and 6). A robustness check using different consensus thresholds from 70–80% indicated that Kendall’s W was 1, demonstrating the consistency and stability of the top ten priority rankings (S3 and S4 Tables). The results of a post hoc analysis examining the top ten priorities among physicians, psychologists and psychotherapists are presented in Supporting information (S5 Table). We observed that at least seven of the top ten priorities for all healthcare professionals were consistently identified across these groups.

**Table 4:** Top ten priorities identified by healthcare professionals.

Order	Item	Percentage Agreement (%)	Mean	Table (#)
1	Providing access to healthcare providers who are well-trained to recognize ADHD	100.00	4.74	2
2	Providing access to ADHD services (e.g., CBT, coaching, skills-based training, employment programs)	100.00	4.50	2
3	Research on socio-emotional functioning in ADHD (e.g., self-esteem issues, ability to regulate emotions) and its impact on relationships	98.50	4.50	3
4	Increasing knowledge and training about ADHD and associated stigmas among all healthcare and mental health professionals (e.g., family doctors, nurse practitioners, pharmacists, psychologists, counsellors)	97.30	4.71	3
5	Providing access to funded services for individuals with ADHD and their loved ones (e.g., healthcare coverage for psychological services, and/or affordable options)	97.30	4.59	3
6	Educating personnel in the school systems on how to best support and teach individuals with ADHD	97.20	4.72	3
7	Research on how co-existing experiences (e.g., depression, anxiety) should be considered when diagnosing ADHD	96.30	4.48	2
8	Research on diagnosing ADHD in girls and women	96.30	4.44	2
9	Providing access to resources and services to smaller and/or rural communities	95.90	4.40	3
10	Providing more accessible information and support to navigate the healthcare system and find appropriate services/personnel to assist and advocate for individuals with ADHD	95.90	4.37	3

ADHD = Attention-Deficit/Hyperactivity Disorder; CBT = Cognitive Behavioural Therapy; Mean = mean Likert score; Table = refers to the table number from which the priority item originated.

## Discussion

This study used the Delphi method to establish consensus on ten top priorities that healthcare professionals find important in the clinical care of individuals with ADHD. Access to healthcare professionals and health services specific to ADHD emerged as the highest ranked priorities where providing access to health services was the dominant theme. Another theme that emerged was related to the need for more research in ADHD, particularly on socio-emotional functioning, co-existing conditions and diagnosing ADHD in females. The healthcare professionals also identified increasing knowledge/training and education among healthcare professionals and school systems about ADHD among the top ten priority issues.

In this study, healthcare professionals unanimously agreed that access to health services specific to ADHD care is a high priority issue. Providing access to mental healthcare services is synonymous with improving an individual’s ability to seek and receive adequate and appropriate care. However, most Canadians are unable to access adequate mental health services [38]. Furthermore, individuals with ADHD face even greater challenges in finding healthcare services specific to their needs [10]. A scoping review examining barriers to mental healthcare in Canada identified by healthcare professionals involved 29 eligible moderate-to-high quality studies where specific themes were identified [38]. “Patient accessibility” (health service utilization, service provision, awareness and wait-times); “training/education” (improving skills and knowledge, clinical care guidelines); and “system availability and complexity” (challenges in accessing and finding appropriate resources, need for financial support) emerged as significant mental healthcare barriers [38]. These themes and the related issues are consistent with the findings of our study pertaining to individuals with ADHD. Healthcare professionals identified access to well-trained healthcare professionals in ADHD of paramount importance. Amidst the growing demand for ADHD-related care [9] and the limited availability of publicly funded healthcare providers who specialize in ADHD in Canada [19,39], front-line primary care providers are facing increasing pressure to diagnose and manage individuals with ADHD [40–42]. However, some primary care physicians do not feel adequately trained and/or even prepared to do so [42,43]. A Canadian study found that family physicians referred 67% of uncomplicated patients with ADHD with no apparent psychiatric comorbid conditions, to specialists with expertise in ADHD [19]. Of these patients, 40% were returned to their family physicians by the specialists immediately after treatment or once stabilized on treatment, suggesting that most of these ADHD cases could have been managed by their primary care physician, had they received sufficient training in diagnosing and managing ADHD [20].

Finding adequate resources and navigating the healthcare system for specific services for ADHD is especially difficult in small or rural communities where there is a lack of healthcare professionals and services for ADHD. In our study, healthcare professionals identified the need to provide access to resources and services to smaller and/or rural communities as one of the top ten priorities in ADHD care. These rural regions face significant shortages in mental health providers compared to urban areas and rely heavily on primary care professionals to address needs related to mental health [44]. A survey conducted among primary care physicians in Elgin County, a rural area of Ontario, showed that over 80% of primary care physicians needed additional training in ADHD [42]. Also, rural primary care physicians were found to be underdiagnosing patients who had sufficient medical information in their chart to meet ADHD criteria, and referrals to specialists were delayed by over 47 weeks [45].

There is also an urgent need, identified by healthcare professionals in this study, to have access to funded services such as healthcare services for psychological support for individuals with ADHD and their families. This high priority issue is dependent on government funding which is somewhat complex in Canada compared to other countries like England, Germany and Australia that spend at least 8% of the public health budget on mental health services [46]. The ability to provide community care services offered by psychologists and allied healthcare providers is not a requirement in the Canada Health Act and as a result, funding for such services is limited and varies from province to province [46].Yet, evidence shows that investing in public insurance to provide community services in Canada would decrease the cost of healthcare and lower productivity losses for employers, leading to savings of $255 billion over 30 years [46]. Therefore, the limited access to trained healthcare professionals in ADHD, the lack of insured healthcare and specialized services for ADHD, and insufficient funding all lead to a multitude of challenges that negatively impact the care of those living with ADHD. Addressing these high priority issues related to providing access to healthcare requires advocacy and effective policy reforms in the Canadian healthcare system.

Another common theme that emerged from the top ten priorities identified by healthcare professionals in this study is the need for research in ADHD related to socio-emotional functioning, co-existing conditions, and in the diagnosis of ADHD in females. Emotional dysregulation is a common characteristic among individuals with ADHD that causes substantial impairment across the lifespan [47] with increased psychological distress [48] further impacting function and even relationships [49]. Healthcare professionals recognize that emotional symptoms are not currently part of the core diagnostic criteria for ADHD as reported in the 5^th^ edition of the Diagnostic and Statistical Manual of Mental Disorders (DSM-5) [50]. However, emotional dysregulation can be seen in some individuals with ADHD, and some suggest it should be a core symptom [51]. Our findings on the need for research into socio-emotional functioning in ADHD align well with an international Delphi study that identified the need for more effective interventions for emotional dysregulation in ADHD among its top ten research priorities [52]. In the present study, healthcare professionals have identified an essential need to better understand and manage social and emotional dysfunction in individuals with ADHD by conducting research in this area to provide more tailored interventions to enhance overall well-being.

In addition to the need for research on socio-emotional functioning in ADHD, healthcare professionals in this study also identified the need to research co-existing experiences such as depression and anxiety when diagnosing ADHD as a high priority. Most adults with ADHD (85%) have been shown to have comorbid medical conditions [40]. For example, studies have found prevalence rates for ADHD to be as high as 53% in individuals with depression and 21% in individuals with bipolar disorder [53]. However, due to the presentation of overlapping symptoms across many of these mental health conditions, it can be very difficult for physicians to diagnose ADHD in individuals who have co-existing conditions [40]. Further research is needed to explore the links between these mental health conditions, which would improve the diagnostic efforts made by healthcare professionals to more effectively detect ADHD in individuals with comorbidities.

Also, research in diagnosing ADHD in females was among the top ten priorities identified by healthcare professionals in Canada. Current assessments and diagnostic criteria for ADHD are predominantly based on how ADHD presents in males [54]. However, females may present with different symptoms compared to males with ADHD across the lifespan [54]. Studies have demonstrated that females present with more inattentive symptoms in childhood compared to males, and present with hyperactive/impulsive symptoms in adulthood [55]. As a result, females tend to be diagnosed later in life compared to males, often undergoing more clinical visits before receiving a diagnosis of ADHD, which reflects a longer and more complex diagnostic journey [56]. Research on females with ADHD remains underprioritized because historical studies focused mainly on males, leading to diagnostic criteria and clinical practices that overlooked female symptoms and resulted in later, less frequent diagnoses of ADHD [57]. Despite emerging evidence, translation of research findings into clinical practice remains limited [57]. Therefore, it is not surprising that females are consistently underdiagnosed and undertreated for ADHD symptoms in clinical practice compared to males, largely due to sex differences in ADHD symptom presentation, and diagnostic bias in assessment tools [56,58]. A Delphi study representing professionals from nine international ADHD organizations examined research priorities for ADHD and ranked the identification and treatment of ADHD in females as the second highest research priority [52]. More research is needed to provide the evidence to improve the early detection and management of females with ADHD, which can help to prevent the health disparities that exist as a result of sex-based differences in diagnosis [54].

Healthcare professionals also indicated from the list of top ten priorities that the need for increased knowledge, education and training for healthcare professionals and in school systems is an important issue related to ADHD care in Canada. A recent systematic review involving 30 studies across four continents (North America, Europe, Asia and South America) evaluating barriers to access and service use among children and adolescents found that more than 50% of included studies reported poor acknowledgment and awareness of ADHD symptoms among clinicians and teachers as barriers to accessing care [59]. Moreover, healthcare professionals in this review recognized the need for training on the stigmas associated with ADHD [59]. Studies have shown that caregivers struggle with discussing their children’s ADHD symptoms with their healthcare providers due to fear of judgement [60]. On the other hand, teachers and educators are often poorly educated on ADHD and on how best to support students with this condition. Over 20% of teachers misclassified ADHD medications as being addictive and 10% were hesitant to use prescribed medication for ADHD for their own child, even when this treatment was recommended by a healthcare professional [61]. Therefore, reducing the knowledge gap that exists among healthcare professionals and educators through education and training of ADHD will promote earlier recognition so that individuals with ADHD can receive timely and effective management.

This study has some strengths and limitations. We used a Delphi method with an appropriate number of panelists. This is consistent with other Delphi studies that have recommended panels consisting of 10–100 panelists as optimal [21,62]. In addition, the panelists represented different healthcare professionals involved in the clinical care of individuals with ADHD across Canada [30], the survey response rates were high in all rounds, and we followed and reported on the quality measures of our study, and on best practices for conducting Delphi studies [35,36]. A limitation of the study is we were unable to capture how many initial invitations were sent to eligible healthcare professionals as the invitations were first distributed by national ADHD organizations (CADDRA and CADDAC) using their confidential mailing lists and some invitations may have been circulated by participants via other social media platforms. Another study limitation is in the selection and sampling of healthcare professionals as panelists. No specific screening of their actual level of expertise in ADHD was determined. The panelists in this study were largely females, from Caucasian/European/White ethnic backgrounds, working in urban areas, with a higher proportion residing in Ontario, and there was also no representation of healthcare professionals from Canada’s northern territories (i.e., Yukon, Northwest Territories, and Nunavut). These factors may have influenced the determination of the top ten priorities for ADHD care. While this study involved a heterogeneous and diverse group of healthcare professionals, the perspectives of patients with ADHD were not included. The findings of this study are reflective of healthcare professionals working in Canada and may not be generalizable to other countries with different healthcare systems.

## Conclusions

This study provides valuable insights from healthcare professionals in Canada on the top ten priorities related to ADHD care. Individuals with ADHD are not receiving adequate access to well-trained healthcare professionals and there is a lack of funded mental health services to meet the needs of this population. The delivery of services in the Canadian healthcare system needs to change in order to accommodate individuals with ADHD. New national strategies are required to address the lack of access to care, to provide research opportunities into ADHD and to raise awareness of ADHD broadly. Policy changes at the government level are urgently needed to increase funding for healthcare professionals who provide community-based services for ADHD and for new collaborative team-based care models among primary care physicians, specialists and other healthcare professionals to provide cost-effective care. Examples are integrated care models that embed behavioural health clinicians within primary care as well as training programs for primary care professionals in ADHD. The top ten priorities identified by Canadian healthcare professionals will inform planning of resources needed to optimize ADHD care. Future research needs to address effective and cost-effective strategies geared to providing access to care for individuals with ADHD by exploring comprehensive team-based care models and addressing barriers related to education and training of ADHD in healthcare and school systems.

## Supporting information

S1 Table 21 Predetermined items. ADHD = Attention-Deficit/Hyperactivity Disorder; CBT = Cognitive Behavioural Therapy.
(DOCX)

S2 Table Panelist characteristics from Round 1 to Round 3. ^a^Panelists that did not provide a response for “Age Category”. ^b^Panelists selected “Other” because they identified a clinical discipline not otherwise categorized. This included 11 panelists in Round 1, who identified: ADHD coaching, social work (3), forensic psychiatry, internal medicine, grade 6–10 school counselling, screening and referral, occupational therapy, behavioural analysis, and children and families impacted by ADHD. In Round 2, the 10 panelists were identical to the other categories except without ADHD coaching.
(DOCX)

S3 Table Ranking of all 21 predetermined items from highest to lowest identified by healthcare professionals in Round 2 (N = 82). ADHD = Attention-Deficit/Hyperactivity Disorder; CBT: = Cognitive Behavioural Therapy; CI = confidence interval; IQR = Interquartile Range; Max = maximum Likert score; Mean = mean Likert score; Median = median Likert score; Min = minimum Likert score; N = number of healthcare professionals that responded; SD = standard deviation.
(DOCX)

S4 Table Ranking of all 34 items from highest to lowest identified by healthcare professionals in Round 3 (n = 73). ADHD = Attention-Deficit/Hyperactivity Disorder; CI = confidence interval; IQR = Interquartile Range; Max = maximum Likert score; Mean = mean Likert score; Median = median Likert score; Min = minimum Likert score; N = number of healthcare professionals that responded; SD = standard deviation.
(DOCX)

S5 Table Subgroup analyses among healthcare professionals. (DOCX)

S1 Appendix Derivation of 34 new items. (DOCX)

S1 Checklist DELPHISTAR. (DOCX)

S2 Checklist ACCORD. (DOCX)

S1 Survey All surveys pertaining to healthcare professionals. (DOCX)

S1 Dataset Minimal data sets. (XLSX)
