# Supplementary material for: Top ten priorities identified by healthcare professionals to support the clinical care of individuals with attention-deficit/hyperactivity disorder: A Canadian Delphi study
Source: PLoS One. 2025 Dec 19;20(12):e0339378. doi: 10.1371/journal.pone.0339378 (PMC12716771; doi:10.1371/journal.pone.0339378)
Supplement: S1 Table — ADHD = Attention-Deficit/Hyperactivity Disorder; CBT = Cognitive Behavioural Therapy. (DOCX) [file pone.0339378.s001.docx]

**S1 Table.** 21 Predetermined items

| **Item** | |
| --- | --- |
| 1 | Providing access to healthcare providers who are well-trained to recognize ADHD |
| 2 | Providing access to ADHD services (e.g. CBT, coaching, skills-based training, employment programs, etc.) |
| 3 | Research on how co-existing experiences (e.g. depression, anxiety) should be considered when diagnosing ADHD |
| 4 | Research on diagnosing ADHD in girls and women |
| 5 | Increasing knowledge about ADHD among teachers and educators |
| 6 | Increasing knowledge about ADHD among parents |
| 7 | Providing access to support for families (spouses, parents, siblings) |
| 8 | Creating new tools to capture how ADHD impacts social relationships and emotion regulation |
| 9 | Providing general funding for ADHD research |
| 10 | Research on the long-term consequences of untreated ADHD |
| 11 | Research on what should be included when diagnosing ADHD (e.g. cognitive assessment) |
| 12 | Research on what it means to be “impaired” by symptoms of ADHD |
| 13 | Research on new non-drug treatments |
| 14 | Research on how ADHD impacts families (parents, partners, siblings) |
| 15 | Research on how well treatment works, and how safe it is, for older adults (age 50+) |
| 16 | Increasing awareness about ADHD among the general public (e.g. through national campaigns) |
| 17 | Increasing awareness about ADHD among employers and in workplaces |
| 18 | Research on the benefits of treatments, relative to their costs (“cost-benefit analysis”) |
| 19 | Research on how to improve treatment compliance (i.e. making sure people take their medication and/or follow their treatment plan) |
| 20 | Research on diagnosing ADHD in older adults (age 50+) |
| 21 | Providing housing programs for people with ADHD |
|  |  |

ADHD=Attention-Deficit/Hyperactivity Disorder; CBT=Cognitive Behavioural Therapy.
