# Supplementary material for: Top ten priorities identified by healthcare professionals to support the clinical care of individuals with attention-deficit/hyperactivity disorder: A Canadian Delphi study
Source: PLoS One. 2025 Dec 19;20(12):e0339378. doi: 10.1371/journal.pone.0339378 (PMC12716771; doi:10.1371/journal.pone.0339378)
Supplement: S2 Table — This included 11 panelists in Round 1, who identified: ADHD coaching, social work (3), forensic psychiatry, internal medicine, grade 6–10 school counselling, screening and referral, occupational therapy, behavioural analysis, and children and families impacted by ADHD. In Round 2, the 10 panelists were identical to the other categories except without ADHD coaching. (DOCX) [file pone.0339378.s002.docx]

**S2 Table. Panelist characteristics from Round 1 to Round 3**

| **Characteristics** | **Round 1 (N = 96)** | | **Round 2 (N = 82)** | | **Round 3 (N = 73)** | |
| --- | --- | --- | --- | --- | --- | --- |
| **Gender (n, %)** |  |  |  |  |  |  |
| Female | 73 | (76.0) | 66 | (80.5) | 58 | (79.5) |
| Male | 20 | (20.8) | 14 | (17.1) | 12 | (16.4) |
| Other | 2 | (2.1) | 1 | (1.2) | 2 | (2.7) |
| Prefer not to say | 1 | (1.0) | 1 | (1.2) | 1 | (1.4) |
| **Age Category (n, %)** |  |  |  |  |  |  |
| 25-39 | 22 | (22.9) | 19 | (23.2) | 16 | (21.9) |
| 40-59 | 43 | (44.8) | 36 | (43.9) | 34 | (46.6) |
| 60+ | 31 | (32.3) | 27 | (32.9) | 23 | (31.5) |
| Missing**^a^** | 1 | (1.0) | 1 | (1.2) |  | - |
| **Province (n, %)** |  |  |  |  |  |  |
| Alberta | 19 | (19.8) | 15 | (18.3) | 14 | (19.2) |
| British Columbia | 11 | (11.5) | 11 | (13.4) | 10 | (13.7) |
| Manitoba | 2 | (2.1) | 1 | (1.2) | 1 | (1.4) |
| New Brunswick | 2 | (2.1) | 2 | (2.4) | 2 | (2.7) |
| Newfoundland and Labrador | 7 | (7.3) | 5 | (6.1) | 6 | (8.2) |
| Nova Scotia | 3 | (3.1) | 3 | (3.7) | 1 | (1.4) |
| Ontario | 30 | (31.2) | 27 | (32.9) | 21 | (28.8) |
| Prince Edward Island | 2 | (2.1) | 2 | (2.4) | 2 | (2.7) |
| Quebec | 16 | (16.7) | 12 | (14.6) | 13 | (17.8) |
| Saskatchewan | 4 | (4.2) | 4 | (4.9) | 3 | (4.1) |
| **Clinical Discipline (n, %)** |  |  |  |  |  |  |
| Adult Psychiatry | 9 | (9.4) | 9 | (11.0) | 4 | (5.5) |
| Adult Psychology | 12 | (12.5) | 10 | (12.2) | 10 | (13.7) |
| Child & Adolescent Psychiatry | 5 | (5.2) | 4 | (4.9) | 3 | (4.1) |
| Child & Adolescent Psychology | 9 | (9.4) | 9 | (11.0) | 8 | (11.0) |
| Psychotherapy | 15 | (15.6) | 15 | (18.3) | 14 | (19.2) |
| Family Medicine | 12 | (12.5) | 9 | (11.0) | 10 | (13.7) |
| Pediatrics | 9 | (9.4) | 7 | (8.5) | 7 | (9.6) |
| Nursing | 4 | (4.2) | 4 | (4.9) | 3 | (4.1) |
| Other^b^ | 11 | (11.5) | 10 | (12.2) | 5 | (6.8) |
| Missing | 10 | (10.4) | 5 | (6.1) | 9 | (12.3) |
|  |  |  |  |  |  |  |

^a^Panelists that did not provide a response for “Age Category”.

^b^Panelists selected “Other” because they identified a clinical discipline not otherwise categorized. This included 11 panelists in Round 1, who identified: ADHD coaching, social work (3), forensic psychiatry, internal medicine, grade 6-10 school counselling, screening and referral, occupational therapy, behavioural analysis, and children and families impacted by ADHD. In Round 2, the 10 panelists were identical to the other categories except without ADHD coaching.
