# Supplementary material for: Top ten priorities identified by healthcare professionals to support the clinical care of individuals with attention-deficit/hyperactivity disorder: A Canadian Delphi study
Source: PLoS One. 2025 Dec 19;20(12):e0339378. doi: 10.1371/journal.pone.0339378 (PMC12716771; doi:10.1371/journal.pone.0339378)
Supplement: S3 Table — ADHD = Attention-Deficit/Hyperactivity Disorder; CBT: = Cognitive Behavioural Therapy; CI = confidence interval; IQR = Interquartile Range; Max = maximum Likert score; Mean = mean Likert score; Median = median Likert score; Min = minimum Likert score; N = number of healthcare professionals that responded; SD = standard deviation. (DOCX) [file pone.0339378.s003.docx]

**S3 Table. Ranking of all 21 predetermined items from highest to lowest identified by healthcare professionals in Round 2 (N=82)**

| **Order** | **Item** | **Percentage Agreement (%)** | **Mean** | **95% CI** | **SD** | **Median** | **IQR** | **Min** | **Max** | **N** |
| --- | --- | --- | --- | --- | --- | --- | --- | --- | --- | --- |
| 1 | Providing access to healthcare providers who are well-trained to recognize ADHD | 100.00 | 4.74 | 4.65-4.84 | 0.44 | 5.00 | 0.75 | 4.00 | 5.00 | 82 |
| 2 | Providing access to ADHD services (e.g. CBT, coaching, skills-based training, employment programs) | 100.00 | 4.50 | 4.39-4.61 | 0.50 | 4.50 | 1.00 | 4.00 | 5.00 | 82 |
| 3 | Research on how co-existing experiences (e.g. depression, anxiety) should be considered when diagnosing ADHD | 96.30 | 4.48 | 4.35-4.61 | 0.57 | 5.00 | 1.00 | 3.00 | 5.00 | 81 |
| 4 | Research on diagnosing ADHD in girls and women | 96.30 | 4.44 | 4.32-4.57 | 0.57 | 4.00 | 1.00 | 3.00 | 5.00 | 81 |
| 5 | Increasing knowledge about ADHD among teachers and educators | 93.90 | 4.54 | 4.39-4.68 | 0.65 | 5.00 | 1.00 | 2.00 | 5.00 | 82 |
| 6 | Increasing knowledge about ADHD among parents | 93.90 | 4.34 | 4.20-4.48 | 0.63 | 4.00 | 1.00 | 2.00 | 5.00 | 82 |
| 7 | Providing access to support for families (spouses, parents, siblings) | 89.00 | 4.16 | 4.01-4.31 | 0.68 | 4.00 | 1.00 | 2.00 | 5.00 | 82 |
| 8 | Creating new tools to capture how ADHD impacts social relationships and emotion regulation | 87.20 | 4.28 | 4.10-4.46 | 0.79 | 4.00 | 1.00 | 2.00 | 5.00 | 78 |
| 9 | Providing general funding for ADHD research | 87.20 | 4.09 | 3.93-4.25 | 0.71 | 4.00 | 0.75 | 2.00 | 5.00 | 78 |
| 10 | Research on the long-term consequences of untreated ADHD | 85.90 | 4.13 | 3.94-4.32 | 0.84 | 4.00 | 1.00 | 2.00 | 5.00 | 78 |
| 11 | Research on what should be included when diagnosing ADHD (e.g. cognitive assessment) | 85.90 | 4.03 | 3.82-4.23 | 0.93 | 4.00 | 1.00 | 1.00 | 5.00 | 78 |
| 12 | Research on what it means to be “impaired” by symptoms of ADHD | 82.10 | 4.01 | 3.84-4.19 | 0.76 | 4.00 | 0.00 | 2.00 | 5.00 | 78 |
| 13 | Research on new non-drug treatments | 80.80 | 3.90 | 3.71-4.08 | 0.83 | 4.00 | 0.00 | 2.00 | 5.00 | 78 |
| 14 | Research on how ADHD impacts families (parents, partners, siblings) | 78.90 | 3.95 | 3.73-4.17 | 0.83 | 4.00 | 0.00 | 1.00 | 5.00 | 57 |
| 15 | Research on how well treatment works, and how safe it is, for older adults (age 50+) | 77.80 | 3.90 | 3.72-4.08 | 0.80 | 4.00 | 0.00 | 2.00 | 5.00 | 81 |
| 16 | Increasing awareness about ADHD among the general public (e.g. through national campaigns) | 76.80 | 3.83 | 3.69-3.97 | 0.64 | 4.00 | 0.00 | 2.00 | 5.00 | 82 |
| 17 | Increasing awareness about ADHD among employers and in workplaces | 75.60 | 3.91 | 3.74-4.08 | 0.77 | 4.00 | 0.00 | 2.00 | 5.00 | 82 |
| 18 | Research on the benefits of treatments, relative to their costs (“cost-benefit analysis”) | 74.40 | 3.90 | 3.72-4.07 | 0.78 | 4.00 | 0.75 | 2.00 | 5.00 | 78 |
| 19 | Research on how to improve treatment compliance (i.e. making sure people take their medication and/or follow their treatment plan) | 65.40 | 3.73 | 3.55-3.91 | 0.78 | 4.00 | 1.00 | 2.00 | 5.00 | 78 |
| 20 | Research on diagnosing ADHD in older adults (age 50+) | 63.00 | 3.68 | 3.49-3.87 | 0.85 | 4.00 | 1.00 | 2.00 | 5.00 | 81 |
| 21 | Providing housing programs for people with ADHD | 11.50 | 2.54 | 2.34-2.74 | 0.89 | 3.00 | 1.00 | 1.00 | 5.00 | 78 |
|  |  |  |  |  |  |  |  |  |  |  |
| ADHD=Attention-Deficit/Hyperactivity Disorder; Cognitive Behavioural Therapy; CI=Confidence Interval; IQR=Interquartile Range; Max=maximum Likert score; Mean=mean Likert score; Median=median Likert score; Min=minimum Likert score; N=number of healthcare professionals that responded; SD=Standard Deviation. | | | | | | | | | | |
