# Supplementary material for: Top ten priorities identified by healthcare professionals to support the clinical care of individuals with attention-deficit/hyperactivity disorder: A Canadian Delphi study
Source: PLoS One. 2025 Dec 19;20(12):e0339378. doi: 10.1371/journal.pone.0339378 (PMC12716771; doi:10.1371/journal.pone.0339378)
Supplement: S4 Table — ADHD = Attention-Deficit/Hyperactivity Disorder; CI = confidence interval; IQR = Interquartile Range; Max = maximum Likert score; Mean = mean Likert score; Median = median Likert score; Min = minimum Likert score; N = number of healthcare professionals that responded; SD = standard deviation. (DOCX) [file pone.0339378.s004.docx]

**S4 Table. Ranking of all 34 items from highest to lowest identified by healthcare professionals in Round 3 (n=73)**

| **Order** | **Item** | **Percentage Agreement (%)** | **Mean** | **95% CI** | **SD** | **Median** | **IQR** | **Min** | **Max** | **N** |
| --- | --- | --- | --- | --- | --- | --- | --- | --- | --- | --- |
| 1 | Research on socio-emotional functioning in ADHD (e.g. self-esteem issues, ability to regulate emotions) and its impact on relationships | 98.50 | 4.50 | 4.37-4.63 | 0.53 | 5.00 | 1.00 | 3.00 | 5.00 | 68 |
| 2 | Increasing knowledge and training about ADHD and associated stigmas among all healthcare and mental health professionals (e.g. family doctors, nurse practitioners, pharmacists, psychologists, counsellors) | 97.30 | 4.71 | 4.58-4.84 | 0.56 | 5.00 | 0.00 | 2.00 | 5.00 | 73 |
| 3 | Providing access to funded services for individuals with ADHD and their loved ones (e.g. healthcare coverage for psychological services, and/or affordable options) | 97.30 | 4.59 | 4.46-4.72 | 0.55 | 5.00 | 1.00 | 3.00 | 5.00 | 73 |
| 4 | Educating personnel in the school systems on how to best support and teach individuals with ADHD | 97.20 | 4.72 | 4.60-4.84 | 0.51 | 5.00 | 0.50 | 3.00 | 5.00 | 71 |
| 5 | Providing access to resources and services to smaller and/or rural communities | 95.90 | 4.41 | 4.28-4.54 | 0.57 | 4.00 | 1.00 | 3.00 | 5.00 | 73 |
| 6 | Providing more accessible information and support to navigate the healthcare system and find appropriate services/personnel to assist and advocate for individuals with ADHD | 95.90 | 4.37 | 4.24-4.50 | 0.57 | 4.00 | 1.00 | 3.00 | 5.00 | 73 |
| 7 | Optimizing the assessment process through the use of validated tools to improve early diagnosis and diagnostic accuracy, and reduce misdiagnosis | 93.20 | 4.45 | 4.31-4.60 | 0.62 | 5.00 | 1.00 | 3.00 | 5.00 | 73 |
| 8 | Research on best treatments for addictions within the context of ADHD (e.g. substances, gaming, gambling, and screens) | 92.40 | 4.48 | 4.33-4.64 | 0.64 | 5.00 | 1.00 | 3.00 | 5.00 | 66 |
| 9 | Identifying delays/barriers to assessment and treatment, and the impacts they may have on different systems | 91.80 | 4.49 | 4.34-4.64 | 0.65 | 5.00 | 1.00 | 3.00 | 5.00 | 73 |
| 10 | Increasing general awareness of ADHD and its impacts in girls and women (e.g. among healthcare providers, across the lifespan, education, workplace) | 91.20 | 4.31 | 4.12-4.50 | 0.80 | 4.00 | 1.00 | 2.00 | 5.00 | 68 |
| 11 | Research on recognizing and diagnosing ADHD in mid-life (ages 35-50) | 91.20 | 4.12 | 3.96-4.27 | 0.64 | 4.00 | 0.00 | 2.00 | 5.00 | 68 |
| 12 | Increasing understanding of ADHD as a condition warranting recognition by government and educational systems | 90.80 | 4.48 | 4.31-4.64 | 0.66 | 5.00 | 1.00 | 3.00 | 5.00 | 65 |
| 13 | Providing individuals with ADHD with the tools, information, and strategies to self-advocate | 90.40 | 4.21 | 4.05-4.37 | 0.69 | 4.00 | 1.00 | 2.00 | 5.00 | 73 |
| 14 | Research on the impact of hormones (e.g. hormonal fluctuations, hormone replacement therapy, or contraceptives) on ADHD symptoms, and their interactions with ADHD medications | 89.70 | 4.38 | 4.19-4.57 | 0.79 | 5.00 | 1.00 | 2.00 | 5.00 | 68 |
| 15 | Educating individuals with ADHD and their loved ones about medication management, different medications available, and best treatment options | 89.40 | 4.23 | 4.05-4.40 | 0.72 | 4.00 | 1.00 | 2.00 | 5.00 | 66 |
| 16 | Increasing the availability of adapted supports within the school system (e.g. report cards specific to students with ADHD, ADHD-friendly learning and aftercare programs) | 88.70 | 4.24 | 4.08-4.40 | 0.69 | 4.00 | 1.00 | 2.00 | 5.00 | 71 |
| 17 | Researching how different treatments affect different individuals in the short- and long-term using a person-centered approach to tailor treatments to all ADHD individuals | 87.90 | 4.29 | 4.12-4.45 | 0.67 | 4.00 | 1.00 | 3.00 | 5.00 | 66 |
| 18 | Increasing the availability of social support networks for individuals, couples, and families with ADHD | 87.70 | 4.08 | 3.95-4.22 | 0.57 | 4.00 | 0.00 | 3.00 | 5.00 | 73 |
| 19 | Increasing awareness among the general public about treated versus untreated ADHD and its implications (e.g. through awareness campaigns, school presentations and healthcare presentations) | 87.10 | 4.16 | 3.98-4.34 | 0.75 | 4.00 | 1.00 | 2.00 | 5.00 | 70 |
| 20 | Research to expand our knowledge of ADHD and co-occurring health-related conditions (e.g. sleep, eating, oral health, personal hygiene) | 85.30 | 4.10 | 3.91-4.30 | 0.81 | 4.00 | 1.00 | 1.00 | 5.00 | 68 |
| 21 | Including people with lived experience in the process of research about ADHD | 84.60 | 4.28 | 4.09-4.47 | 0.76 | 4.00 | 1.00 | 2.00 | 5.00 | 65 |
| 22 | Increase public awareness of the different ways ADHD can present (e.g. on a spectrum, with different symptom types, can be “masked”) | 82.90 | 4.00 | 3.80-4.20 | 0.83 | 4.00 | 0.75 | 2.00 | 5.00 | 70 |
| 23 | Research to understand the prevalence and unique experiences of people with ADHD and other mental health challenges | 82.40 | 4.13 | 3.95-4.32 | 0.77 | 4.00 | 1.00 | 2.00 | 5.00 | 68 |
| 24 | Providing access to holistic treatment options supported by multi-disciplinary teams (e.g. medication, nutrition, occupational therapy) embedded within systems like workplace and education | 81.80 | 3.94 | 3.73-4.15 | 0.86 | 4.00 | 0.00 | 1.00 | 5.00 | 66 |
| 25 | Research on the impact of ADHD medications on hormonal fluctuations and reproductive health | 80.90 | 4.21 | 4.00-4.41 | 0.86 | 4.00 | 1.00 | 2.00 | 5.00 | 68 |
| 26 | Increasing knowledge and awareness about the impact of inter-generational ADHD (many generations of ADHD within a family) | 80.90 | 4.01 | 3.83-4.20 | 0.76 | 4.00 | 0.25 | 2.00 | 5.00 | 68 |
| 27 | Continuing to work towards a better understanding of the causes of ADHD (e.g. genetics, hereditability, neurological mechanisms, risk factors) | 80.00 | 3.92 | 3.72-4.13 | 0.82 | 4.00 | 0.00 | 1.00 | 5.00 | 65 |
| 28 | Expanding our understanding of ADHD in under-served or marginalized populations (e.g. ethnic minorities, queer and gender-diverse communities, and Indigenous groups) | 79.40 | 4.19 | 3.98-4.40 | 0.87 | 4.00 | 1.00 | 2.00 | 5.00 | 68 |
| 29 | Providing basic general training in recognizing ADHD to all personnel who interact with youth in their line of work (e.g. police, dentists, social workers, corrections officers, educators) | 78.90 | 3.97 | 3.79-4.15 | 0.76 | 4.00 | 0.00 | 2.00 | 5.00 | 71 |
| 30 | Increasing job opportunities and workplace accommodations for all employees with ADHD | 76.10 | 3.92 | 3.74-4.09 | 0.75 | 4.00 | 0.00 | 2.00 | 5.00 | 71 |
| 31 | Research on optimizing existing non-drug treatments (e.g. meditation and mindfulness, psychotherapy, physical activity, acupuncture) | 74.20 | 3.82 | 3.57-4.06 | 0.99 | 4.00 | 0.75 | 1.00 | 5.00 | 66 |
| 32 | Targeted research examining the stigmatization of ADHD (e.g. self-stigma, parental stigma, stigmatization in schools or classrooms) | 70.80 | 3.80 | 3.58-4.02 | 0.90 | 4.00 | 1.00 | 1.00 | 5.00 | 65 |
| 33 | Encouraging positive-directed research to better understand the unique strengths of those with ADHD | 67.70 | 3.69 | 3.44-3.95 | 1.03 | 4.00 | 1.00 | 1.00 | 5.00 | 65 |
| 34 | Redefining ADHD in a more positive/normative way as a facet of neurodiversity, to de-stigmatize and de-medicalize it (e.g. by changing ADHD terminology to remove words like disorder, disability) | 58.60 | 3.41 | 3.11-3.72 | 1.27 | 4.00 | 1.00 | 1.00 | 5.00 | 70 |
|  |  |  |  |  |  |  |  |  |  |  |
| ADHD=Attention-Deficit/Hyperactivity Disorder; CI=Confidence Interval; IQR=Interquartile Range; Max=maximum Likert score; Mean=mean Likert score; Median=median Likert score; Min=minimum Likert score; N=number of healthcare professionals that responded; SD=Standard Deviation. | | | | | | | | | | |
