# Supplementary material for: Top ten priorities identified by healthcare professionals to support the clinical care of individuals with attention-deficit/hyperactivity disorder: A Canadian Delphi study
Source: PLoS One. 2025 Dec 19;20(12):e0339378. doi: 10.1371/journal.pone.0339378 (PMC12716771; doi:10.1371/journal.pone.0339378)
Supplement: S5 Table — (DOCX) [file pone.0339378.s005.docx]

**S5 Table. Subgroup analyses among healthcare professionals**

Table of Contents

[**S5A Table. Round 2 for physicians (N=29) 2**](#_Toc213348172)

[**S5B Table. Round 3 for physicians (N=24) 4**](#_Toc213348173)

[**S5C Table. Top ten priorities for physicians 9**](#_Toc213348174)

[**S5D Table. Round 2 for psychologists (N=19) 10**](#_Toc213348175)

[**S5E Table. Round 3 for psychologists (N=18) 12**](#_Toc213348176)

[**S5F Table. Top ten priorities for psychologists 17**](#_Toc213348177)

[**S5G Table. Round 2 for psychotherapists (N=15) 18**](#_Toc213348178)

[**S5H Table. Round 3 for psychotherapists (N=14) 21**](#_Toc213348179)

[**S5I Table. Top ten priorities for psychotherapists 26**](#_Toc213348180)

# S5A Table. Round 2 for physicians (N=29)

| **Order** | **Item** | **Percentage Agreement (%)** | **Mean** | **95% CI** | **SD** | **Median** | **IQR** | **Min** | **Max** | **N** |
| --- | --- | --- | --- | --- | --- | --- | --- | --- | --- | --- |
|  |  |  |  |  |  |  |  |  |  |  |
| 1 | Increasing knowledge about ADHD among teachers and educators | 100.00 | 4.69 | 4.51-4.87 | 0.47 | 5.00 | 1.00 | 4.00 | 5.00 | 29 |
|  |  |  |  |  |  |  |  |  |  |  |
| 2 | Providing access to healthcare providers who are well-trained to recognize ADHD | 100.00 | 4.66 | 4.47-4.84 | 0.48 | 5.00 | 1.00 | 4.00 | 5.00 | 29 |
|  |  |  |  |  |  |  |  |  |  |  |
| 3 | Providing access to ADHD services (e.g. CBT, coaching, skills-based training, employment programs) | 100.00 | 4.55 | 4.36-4.74 | 0.51 | 5.00 | 1.00 | 4.00 | 5.00 | 29 |
|  |  |  |  |  |  |  |  |  |  |  |
| 4 | Providing access to support for families (spouses, parents, siblings) | 100.00 | 4.34 | 4.16-4.53 | 0.48 | 4.00 | 1.00 | 4.00 | 5.00 | 29 |
|  |  |  |  |  |  |  |  |  |  |  |
| 5 | Research on how co-existing experiences (e.g. depression, anxiety) should be considered when diagnosing ADHD | 96.60 | 4.48 | 4.26-4.70 | 0.57 | 5.00 | 1.00 | 3.00 | 5.00 | 29 |
|  |  |  |  |  |  |  |  |  |  |  |
| 6 | Increasing knowledge about ADHD among parents | 96.60 | 4.45 | 4.23-4.67 | 0.57 | 4.00 | 1.00 | 3.00 | 5.00 | 29 |
|  |  |  |  |  |  |  |  |  |  |  |
| 7 | Research on diagnosing ADHD in girls and women | 96.60 | 4.41 | 4.20-4.63 | 0.57 | 4.00 | 1.00 | 3.00 | 5.00 | 29 |
|  |  |  |  |  |  |  |  |  |  |  |
| 8 | Research on new non-drug treatments | 89.30 | 4.11 | 3.84-4.37 | 0.69 | 4.00 | 0.25 | 2.00 | 5.00 | 28 |
|  |  |  |  |  |  |  |  |  |  |  |
| 9 | Research on how well treatment works, and how safe it is, for older adults (age 50+) | 86.20 | 3.97 | 3.62-4.31 | 0.91 | 4.00 | 0.00 | 2.00 | 5.00 | 29 |
|  |  |  |  |  |  |  |  |  |  |  |
| 10 | Research on the long-term consequences of untreated ADHD | 85.70 | 4.18 | 3.81-4.54 | 0.94 | 4.00 | 1.00 | 2.00 | 5.00 | 28 |
|  |  |  |  |  |  |  |  |  |  |  |
| 11 | Research on the benefits of treatments, relative to their costs ("cost-benefit analysis") | 85.70 | 4.11 | 3.86-4.35 | 0.63 | 4.00 | 0.25 | 3.00 | 5.00 | 28 |
|  |  |  |  |  |  |  |  |  |  |  |
| 12 | Increasing awareness about ADHD among the general public (e.g. through national campaigns) | 82.80 | 3.93 | 3.73-4.13 | 0.53 | 4.00 | 0.00 | 3.00 | 5.00 | 29 |
|  |  |  |  |  |  |  |  |  |  |  |
| 13 | Research on what it means to be "impaired" by symptoms of ADHD | 82.10 | 4.04 | 3.75-4.32 | 0.74 | 4.00 | 0.25 | 2.00 | 5.00 | 28 |
|  |  |  |  |  |  |  |  |  |  |  |
| 14 | Providing general funding for ADHD research | 82.10 | 4.04 | 3.75-4.32 | 0.74 | 4.00 | 0.25 | 2.00 | 5.00 | 28 |
|  |  |  |  |  |  |  |  |  |  |  |
| 15 | Research on what should be included when diagnosing ADHD (e.g. cognitive assessment) | 82.10 | 3.96 | 3.66-4.27 | 0.79 | 4.00 | 0.00 | 2.00 | 5.00 | 28 |
|  |  |  |  |  |  |  |  |  |  |  |
| 16 | Creating new tools to capture how ADHD impacts social relationships and emotion regulation | 78.60 | 4.07 | 3.69-4.45 | 0.98 | 4.00 | 1.00 | 2.00 | 5.00 | 28 |
|  |  |  |  |  |  |  |  |  |  |  |
| 17 | Research on how ADHD impacts families (parents, partners, siblings) | 76.50 | 3.88 | 3.31-4.45 | 1.11 | 4.00 | 1.00 | 1.00 | 5.00 | 17 |
|  |  |  |  |  |  |  |  |  |  |  |
| 18 | Increasing awareness about ADHD among employers and in workplaces | 75.90 | 4.00 | 3.70-4.30 | 0.80 | 4.00 | 1.00 | 2.00 | 5.00 | 29 |
|  |  |  |  |  |  |  |  |  |  |  |
| 19 | Research on how to improve treatment compliance (i.e. making sure people take their medication and/or follow their treatment plan) | 71.40 | 3.89 | 3.59-4.20 | 0.79 | 4.00 | 1.00 | 2.00 | 5.00 | 28 |
|  |  |  |  |  |  |  |  |  |  |  |
| 20 | Research on diagnosing ADHD in older adults (age 50+) | 62.10 | 3.52 | 3.19-3.85 | 0.87 | 4.00 | 1.00 | 2.00 | 5.00 | 29 |
|  |  |  |  |  |  |  |  |  |  |  |
| 21 | Providing housing programs for people with ADHD | 17.90 | 2.68 | 2.33-3.03 | 0.90 | 3.00 | 1.00 | 1.00 | 4.00 | 28 |
|  |  |  |  |  |  |  |  |  |  |  |

ADHD=Attention-Deficit/Hyperactivity Disorder; CBT=Cognitive Behavioural Therapy; CI=Confidence Interval; IQR=Interquartile Range; Max=maximum Likert score; Mean=mean Likert score; Median=median Likert score; Min=minimum Likert score; N=number of healthcare professionals that responded; SD=Standard Deviation.

# S5B Table. Round 3 for physicians (N=24)

| **Order** | **Item** | **Percentage Agreement (%)** | **Mean** | **95% CI** | **SD** | **Median** | **IQR** | **Min** | **Max** | **N** |
| --- | --- | --- | --- | --- | --- | --- | --- | --- | --- | --- |
|  |  |  |  |  |  |  |  |  |  |  |
| 1 | Educating personnel in the school systems on how to best support and teach individuals with ADHD | 95.80 | 4.71 | 4.48-4.94 | 0.55 | 5.00 | 0.25 | 3.00 | 5.00 | 24 |
|  |  |  |  |  |  |  |  |  |  |  |
| 2 | Providing access to funded services for individuals with ADHD and their loved ones (e.g. healthcare coverage for psychological services, and/or affordable options) | 95.80 | 4.58 | 4.34-4.83 | 0.58 | 5.00 | 1.00 | 3.00 | 5.00 | 24 |
|  |  |  |  |  |  |  |  |  |  |  |
| 3 | Providing access to resources and services to smaller and/or rural communities | 95.80 | 4.50 | 4.25-4.75 | 0.59 | 5.00 | 1.00 | 3.00 | 5.00 | 24 |
|  |  |  |  |  |  |  |  |  |  |  |
| 4 | Optimizing the assessment process through the use of validated tools to improve early diagnosis and diagnostic accuracy, and reduce misdiagnosis | 95.80 | 4.46 | 4.21-4.71 | 0.59 | 4.50 | 1.00 | 3.00 | 5.00 | 24 |
|  |  |  |  |  |  |  |  |  |  |  |
| 5 | Providing more accessible information and support to navigate the healthcare system and find appropriate services/personnel to assist and advocate for individuals with ADHD | 95.80 | 4.38 | 4.13-4.62 | 0.58 | 4.00 | 1.00 | 3.00 | 5.00 | 24 |
|  |  |  |  |  |  |  |  |  |  |  |
| 6 | Increasing the availability of social support networks for individuals, couples, and families with ADHD | 95.80 | 4.21 | 3.99-4.42 | 0.51 | 4.00 | 0.25 | 3.00 | 5.00 | 24 |
|  |  |  |  |  |  |  |  |  |  |  |
| 7 | Research on socio-emotional functioning in ADHD (e.g. self-esteem issues, ability to regulate emotions) and its impact on relationships | 95.50 | 4.59 | 4.33-4.85 | 0.59 | 5.00 | 1.00 | 3.00 | 5.00 | 22 |
|  |  |  |  |  |  |  |  |  |  |  |
| 8 | Continuing to work towards a better understanding of the causes of ADHD (e.g. genetics, hereditability, neurological mechanisms, risk factors) | 95.20 | 4.10 | 3.72-4.47 | 0.83 | 4.00 | 0.00 | 1.00 | 5.00 | 21 |
|  |  |  |  |  |  |  |  |  |  |  |
| 9 | Increasing knowledge and training about ADHD and associated stigmas among all healthcare and mental health professionals (e.g. family doctors, nurse practitioners, pharmacists, psychologists, counsellors) | 91.70 | 4.62 | 4.30-4.95 | 0.77 | 5.00 | 0.25 | 2.00 | 5.00 | 24 |
|  |  |  |  |  |  |  |  |  |  |  |
| 10 | Identifying delays/barriers to assessment and treatment, and the impacts they may have on different systems | 91.70 | 4.54 | 4.26-4.82 | 0.66 | 5.00 | 1.00 | 3.00 | 5.00 | 24 |
|  |  |  |  |  |  |  |  |  |  |  |
| 11 | Research on best treatments for addictions within the context of ADHD (e.g. substances, gaming, gambling, and screens) | 90.90 | 4.68 | 4.40-4.97 | 0.65 | 5.00 | 0.00 | 3.00 | 5.00 | 22 |
|  |  |  |  |  |  |  |  |  |  |  |
| 12 | Increasing understanding of ADHD as a condition warranting recognition by government and educational systems | 90.50 | 4.57 | 4.26-4.88 | 0.68 | 5.00 | 1.00 | 3.00 | 5.00 | 21 |
|  |  |  |  |  |  |  |  |  |  |  |
| 13 | Increasing the availability of adapted supports within the school system (e.g. report cards specific to students with ADHD, ADHD-friendly learning and aftercare programs) | 87.50 | 4.33 | 4.04-4.63 | 0.70 | 4.00 | 1.00 | 3.00 | 5.00 | 24 |
|  |  |  |  |  |  |  |  |  |  |  |
| 14 | Providing individuals with ADHD with the tools, information, and strategies to self-advocate | 87.50 | 4.21 | 3.88-4.54 | 0.78 | 4.00 | 1.00 | 2.00 | 5.00 | 24 |
|  |  |  |  |  |  |  |  |  |  |  |
| 15 | Increasing awareness among the general public about treated versus untreated ADHD and its implications (e.g. through awareness campaigns, school presentations and healthcare presentations) | 87.00 | 4.39 | 4.03-4.75 | 0.84 | 5.00 | 1.00 | 2.00 | 5.00 | 23 |
|  |  |  |  |  |  |  |  |  |  |  |
| 16 | Researching how different treatments affect different individuals in the short- and long-term using a person-centered approach to tailor treatments to all ADHD individuals | 86.40 | 4.32 | 4.00-4.64 | 0.72 | 4.00 | 1.00 | 3.00 | 5.00 | 22 |
|  |  |  |  |  |  |  |  |  |  |  |
| 17 | Educating individuals with ADHD and their loved ones about medication management, different medications available, and best treatment options | 86.40 | 4.18 | 3.83-4.53 | 0.80 | 4.00 | 1.00 | 2.00 | 5.00 | 22 |
|  |  |  |  |  |  |  |  |  |  |  |
| 18 | Increasing general awareness of ADHD and its impacts in girls and women (e.g. among healthcare providers, across the lifespan, education, workplace) | 86.40 | 4.05 | 3.67-4.42 | 0.84 | 4.00 | 0.75 | 2.00 | 5.00 | 22 |
|  |  |  |  |  |  |  |  |  |  |  |
| 19 | Research on recognizing and diagnosing ADHD in mid-life (35-50) | 86.40 | 4.00 | 3.69-4.31 | 0.69 | 4.00 | 0.00 | 2.00 | 5.00 | 22 |
|  |  |  |  |  |  |  |  |  |  |  |
| 20 | Increase public awareness of the different ways ADHD can present (e.g. on a spectrum, with different symptom types, can be "masked") | 82.60 | 4.04 | 3.66-4.42 | 0.88 | 4.00 | 1.00 | 2.00 | 5.00 | 23 |
|  |  |  |  |  |  |  |  |  |  |  |
| 21 | Expanding our understanding of ADHD in under-served or marginalized populations (e.g. ethnic minorities, queer and gender-diverse communities, and Indigenous groups) | 81.80 | 4.23 | 3.80-4.66 | 0.97 | 4.50 | 1.00 | 2.00 | 5.00 | 22 |
|  |  |  |  |  |  |  |  |  |  |  |
| 22 | Research to understand the prevalence and unique experiences of people with ADHD and other mental health challenges | 81.80 | 4.18 | 3.80-4.56 | 0.85 | 4.00 | 1.00 | 2.00 | 5.00 | 22 |
|  |  |  |  |  |  |  |  |  |  |  |
| 23 | Research on the impact of hormones (e.g. hormonal fluctuations, hormone replacement therapy, or contraceptives) on ADHD symptoms, and their interactions with ADHD medications | 81.80 | 4.05 | 3.65-4.44 | 0.90 | 4.00 | 1.00 | 2.00 | 5.00 | 22 |
|  |  |  |  |  |  |  |  |  |  |  |
| 24 | Targeted research examining the stigmatization of ADHD (e.g. self-stigma, parental stigma, stigmatization in schools or classrooms) | 81.00 | 4.00 | 3.59-4.41 | 0.89 | 4.00 | 1.00 | 2.00 | 5.00 | 21 |
|  |  |  |  |  |  |  |  |  |  |  |
| 25 | Providing basic general training in recognizing ADHD to all personnel who interact with youth in their line of work (e.g. police, dentists, social workers, corrections officers, educators) | 79.20 | 3.96 | 3.64-4.28 | 0.75 | 4.00 | 0.00 | 2.00 | 5.00 | 24 |
|  |  |  |  |  |  |  |  |  |  |  |
| 26 | Research to expand our knowledge of ADHD and co-occurring health-related conditions (e.g. sleep, eating, oral health, personal hygiene) | 77.30 | 3.95 | 3.49-4.42 | 1.05 | 4.00 | 1.00 | 1.00 | 5.00 | 22 |
|  |  |  |  |  |  |  |  |  |  |  |
| 27 | Providing access to holistic treatment options supported by multi-disciplinary teams (e.g. medication, nutrition, occupational therapy) embedded within systems like workplace and education | 77.30 | 3.86 | 3.38-4.34 | 1.08 | 4.00 | 0.75 | 1.00 | 5.00 | 22 |
|  |  |  |  |  |  |  |  |  |  |  |
| 28 | Including people with lived experience in the process of research about ADHD | 76.20 | 4.14 | 3.73-4.56 | 0.91 | 4.00 | 1.00 | 2.00 | 5.00 | 21 |
|  |  |  |  |  |  |  |  |  |  |  |
| 29 | Increasing knowledge and awareness about the impact of inter-generational ADHD (many generations of ADHD within a family) | 72.70 | 3.95 | 3.58-4.33 | 0.84 | 4.00 | 1.50 | 2.00 | 5.00 | 22 |
|  |  |  |  |  |  |  |  |  |  |  |
| 30 | Research on optimizing existing non-drug treatments (e.g. meditation and mindfulness, psychotherapy, physical activity, acupuncture) | 72.70 | 3.64 | 3.08-4.19 | 1.26 | 4.00 | 0.75 | 1.00 | 5.00 | 22 |
|  |  |  |  |  |  |  |  |  |  |  |
| 31 | Encouraging positive-directed research to better understand the unique strengths of those with ADHD | 71.40 | 3.81 | 3.24-4.38 | 1.25 | 4.00 | 2.00 | 1.00 | 5.00 | 21 |
|  |  |  |  |  |  |  |  |  |  |  |
| 32 | Increasing job opportunities and workplace accommodations for all employees with ADHD | 70.80 | 3.83 | 3.47-4.20 | 0.87 | 4.00 | 1.00 | 2.00 | 5.00 | 24 |
|  |  |  |  |  |  |  |  |  |  |  |
| 33 | Research on the impact of ADHD medications on hormonal and reproductive health | 68.20 | 3.95 | 3.56-4.35 | 0.90 | 4.00 | 2.00 | 2.00 | 5.00 | 22 |
|  |  |  |  |  |  |  |  |  |  |  |
| 34 | Redefining ADHD in a more positive/normative way as a facet of neurodiversity, to de-stigmatize and de-medicalize it (e.g. by changing ADHD terminology to remove words like disorder, disability) | 56.50 | 3.30 | 2.71-3.89 | 1.36 | 4.00 | 1.50 | 1.00 | 5.00 | 23 |
|  |  |  |  |  |  |  |  |  |  |  |

ADHD=Attention-Deficit/Hyperactivity Disorder; CI=Confidence Interval; IQR=Interquartile Range; Max=maximum Likert score; Mean=mean Likert score; Median=median Likert score; Min=minimum Likert score; N=number of healthcare professionals that responded; SD=Standard Deviation.

# S5C Table. Top ten priorities for physicians

| **Order** | **Item** | **Percentage Agreement (%)** | **Mean** | **Round** |
| --- | --- | --- | --- | --- |
|  |  |  |  |  |
| 1 | Increasing knowledge about ADHD among teachers and educators | 100.00 | 4.69 | 2 |
|  |  |  |  |  |
| 2 | Providing access to healthcare providers who are well-trained to recognize ADHD | 100.00 | 4.66 | 2 |
|  |  |  |  |  |
| 3 | Providing access to ADHD services (e.g. CBT, coaching, skills-based training, employment programs) | 100.00 | 4.55 | 2 |
|  |  |  |  |  |
| 4 | Providing access to support for families (spouses, parents, siblings) | 100.00 | 4.34 | 2 |
|  |  |  |  |  |
| 5 | Research on how co-existing experiences (e.g., depression, anxiety) should be considered when diagnosing ADHD | 96.60 | 4.48 | 2 |
|  |  |  |  |  |
| 6 | Increasing knowledge about ADHD among parents | 96.60 | 4.45 | 2 |
|  |  |  |  |  |
| 7 | Research on diagnosing ADHD in girls and women | 96.60 | 4.41 | 2 |
|  |  |  |  |  |
| 8 | Educating personnel in the school systems on how to best support and teach individuals with ADHD | 95.80 | 4.71 | 3 |
|  |  |  |  |  |
| 9 | Providing access to funded services for individuals with ADHD and their loved ones (e.g. healthcare coverage for psychological services, and/or affordable options) | 95.80 | 4.58 | 3 |
|  |  |  |  |  |
| 10 | Providing access to resources and services to smaller and/or rural communities | 95.80 | 4.50 | 3 |
|  |  |  |  |  |

ADHD=Attention-Deficit/Hyperactivity Disorder; Cognitive Behavioural Therapy; Mean=mean Likert score.

# S5D Table. Round 2 for psychologists (N=19)

| **Order** | **Item** | **Percentage Agreement (%)** | **Mean** | **95% CI** | **SD** | **Median** | **IQR** | **Min** | **Max** | **N** |
| --- | --- | --- | --- | --- | --- | --- | --- | --- | --- | --- |
|  |  |  |  |  |  |  |  |  |  |  |
| 1 | Providing access to healthcare providers who are well-trained to recognize ADHD | 100.00 | 4.79 | 4.59-4.99 | 0.42 | 5.00 | 0.00 | 4.00 | 5.00 | 19 |
|  |  |  |  |  |  |  |  |  |  |  |
| 2 | Providing access to ADHD services (e.g. CBT, coaching, skills-based training, employment programs) | 100.00 | 4.42 | 4.18-4.67 | 0.51 | 4.00 | 1.00 | 4.00 | 5.00 | 19 |
|  |  |  |  |  |  |  |  |  |  |  |
| 3 | Research on diagnosing ADHD in girls and women | 100.00 | 4.42 | 4.18-4.67 | 0.51 | 4.00 | 1.00 | 4.00 | 5.00 | 19 |
|  |  |  |  |  |  |  |  |  |  |  |
| 4 | Research on how co-existing experiences (e.g. depression, anxiety) should be considered when diagnosing ADHD | 100.00 | 4.42 | 4.18-4.67 | 0.51 | 4.00 | 1.00 | 4.00 | 5.00 | 19 |
|  |  |  |  |  |  |  |  |  |  |  |
| 5 | Increasing knowledge about ADHD among teachers and educators | 94.70 | 4.63 | 4.34-4.92 | 0.60 | 5.00 | 1.00 | 3.00 | 5.00 | 19 |
|  |  |  |  |  |  |  |  |  |  |  |
| 6 | Providing access to support for families (spouses, parents, siblings) | 94.70 | 4.16 | 3.92-4.40 | 0.50 | 4.00 | 0.00 | 3.00 | 5.00 | 19 |
|  |  |  |  |  |  |  |  |  |  |  |
| 7 | Creating new tools to capture how ADHD impacts social relationships and emotion regulation | 94.40 | 4.28 | 3.99-4.56 | 0.57 | 4.00 | 1.00 | 3.00 | 5.00 | 18 |
|  |  |  |  |  |  |  |  |  |  |  |
| 8 | Increasing knowledge about ADHD among parents | 89.50 | 4.16 | 3.87-4.45 | 0.60 | 4.00 | 0.50 | 3.00 | 5.00 | 19 |
|  |  |  |  |  |  |  |  |  |  |  |
| 9 | Research on what it means to be "impaired" by symptoms of ADHD | 88.90 | 4.22 | 3.90-4.54 | 0.65 | 4.00 | 1.00 | 3.00 | 5.00 | 18 |
|  |  |  |  |  |  |  |  |  |  |  |
| 10 | Providing general funding for ADHD research | 88.90 | 4.06 | 3.69-4.42 | 0.73 | 4.00 | 0.00 | 2.00 | 5.00 | 18 |
|  |  |  |  |  |  |  |  |  |  |  |
| 11 | Increasing awareness about ADHD among employers and in workplaces | 84.20 | 3.89 | 3.58-4.21 | 0.66 | 4.00 | 0.00 | 2.00 | 5.00 | 19 |
|  |  |  |  |  |  |  |  |  |  |  |
| 12 | Research on the long-term consequences of untreated ADHD | 83.30 | 4.22 | 3.79-4.66 | 0.88 | 4.00 | 1.00 | 2.00 | 5.00 | 18 |
|  |  |  |  |  |  |  |  |  |  |  |
| 13 | Research on new non-drug treatments | 83.30 | 3.89 | 3.47-4.30 | 0.83 | 4.00 | 0.00 | 2.00 | 5.00 | 18 |
|  |  |  |  |  |  |  |  |  |  |  |
| 14 | Increasing awareness about ADHD among the general public (e.g. through national campaigns) | 78.90 | 3.79 | 3.49-4.09 | 0.63 | 4.00 | 0.00 | 2.00 | 5.00 | 19 |
|  |  |  |  |  |  |  |  |  |  |  |
| 15 | Research on what should be included when diagnosing ADHD (e.g. cognitive assessment) | 77.80 | 3.67 | 3.01-4.33 | 1.33 | 4.00 | 0.00 | 1.00 | 5.00 | 18 |
|  |  |  |  |  |  |  |  |  |  |  |
| 16 | Research on how to improve treatment compliance (i.e. making sure people take their medication and/or follow their treatment plan) | 72.20 | 3.83 | 3.53-4.14 | 0.62 | 4.00 | 0.75 | 3.00 | 5.00 | 18 |
|  |  |  |  |  |  |  |  |  |  |  |
| 17 | Research on how ADHD impacts families (parents, partners, siblings) | 68.80 | 3.81 | 3.46-4.16 | 0.66 | 4.00 | 1.00 | 3.00 | 5.00 | 16 |
|  |  |  |  |  |  |  |  |  |  |  |
| 18 | Research on the benefits of treatments, relative to their costs ("cost-benefit analysis") | 66.70 | 3.67 | 3.25-4.08 | 0.84 | 4.00 | 1.00 | 2.00 | 5.00 | 18 |
|  |  |  |  |  |  |  |  |  |  |  |
| 19 | Research on how well treatment works, and how safe it is, for older adults (age 50+) | 63.20 | 3.79 | 3.38-4.20 | 0.85 | 4.00 | 1.00 | 2.00 | 5.00 | 19 |
|  |  |  |  |  |  |  |  |  |  |  |
| 20 | Research on diagnosing ADHD in older adults (age 50+) | 57.90 | 3.68 | 3.29-4.08 | 0.82 | 4.00 | 1.00 | 2.00 | 5.00 | 19 |
|  |  |  |  |  |  |  |  |  |  |  |
| 21 | Providing housing programs for people with ADHD | 5.60 | 2.44 | 1.96-2.93 | 0.98 | 2.50 | 1.00 | 1.00 | 5.00 | 18 |
|  |  |  |  |  |  |  |  |  |  |  |

ADHD=Attention-Deficit/Hyperactivity Disorder; CBT=Cognitive Behavioural Therapy; CI=Confidence Interval; IQR=Interquartile Range; Max=maximum Likert score; Mean=mean Likert score; Median=median Likert score; Min=minimum Likert score; N=number of healthcare professionals that responded; SD=Standard Deviation.

# S5E Table. Round 3 for psychologists (N=18)

| **Order** | **Item** | **Percentage Agreement (%)** | **Mean** | **95% CI** | **SD** | **Median** | **IQR** | **Min** | **Max** | **N** |
| --- | --- | --- | --- | --- | --- | --- | --- | --- | --- | --- |
|  |  |  |  |  |  |  |  |  |  |  |
| 1 | Increasing knowledge and training about ADHD and associated stigmas among all healthcare and mental health professionals (e.g. family doctors, nurse practitioners, pharmacists, psychologists, counsellors) | 100.00 | 4.78 | 4.57-4.99 | 0.43 | 5.00 | 0.00 | 4.00 | 5.00 | 18 |
|  |  |  |  |  |  |  |  |  |  |  |
| 2 | Providing access to funded services for individuals with ADHD and their loved ones (e.g. healthcare coverage for psychological services, and/or affordable options) | 100.00 | 4.50 | 4.24-4.76 | 0.51 | 4.50 | 1.00 | 4.00 | 5.00 | 18 |
|  |  |  |  |  |  |  |  |  |  |  |
| 3 | Research on socio-emotional functioning in ADHD (e.g. self-esteem issues, ability to regulate emotions) and its impact on relationships | 100.00 | 4.44 | 4.19-4.70 | 0.51 | 4.00 | 1.00 | 4.00 | 5.00 | 18 |
|  |  |  |  |  |  |  |  |  |  |  |
| 4 | Providing access to resources and services to smaller and/or rural communities | 100.00 | 4.39 | 4.14-4.64 | 0.50 | 4.00 | 1.00 | 4.00 | 5.00 | 18 |
|  |  |  |  |  |  |  |  |  |  |  |
| 5 | Providing more accessible information and support to navigate the healthcare system and find appropriate services/personnel to assist and advocate for individuals with ADHD | 100.00 | 4.22 | 4.01-4.43 | 0.43 | 4.00 | 0.00 | 4.00 | 5.00 | 18 |
|  |  |  |  |  |  |  |  |  |  |  |
| 6 | Educating personnel in the school systems on how to best support and teach individuals with ADHD | 94.40 | 4.72 | 4.44-5.01 | 0.57 | 5.00 | 0.00 | 3.00 | 5.00 | 18 |
|  |  |  |  |  |  |  |  |  |  |  |
| 7 | Increasing general awareness of ADHD and its impacts in girls and women (e.g. among healthcare providers, across the lifespan, education, workplace) | 94.40 | 4.67 | 4.37-4.96 | 0.59 | 5.00 | 0.75 | 3.00 | 5.00 | 18 |
|  |  |  |  |  |  |  |  |  |  |  |
| 8 | Research on the impact of hormones (e.g. hormonal fluctuations, hormone replacement therapy, or contraceptives) on ADHD symptoms, and their interactions with ADHD medications | 94.40 | 4.50 | 4.19-4.81 | 0.62 | 5.00 | 1.00 | 3.00 | 5.00 | 18 |
|  |  |  |  |  |  |  |  |  |  |  |
| 9 | Optimizing the assessment process through the use of validated tools to improve early diagnosis and diagnostic accuracy, and reduce misdiagnosis | 94.40 | 4.44 | 4.14-4.75 | 0.62 | 4.50 | 1.00 | 3.00 | 5.00 | 18 |
|  |  |  |  |  |  |  |  |  |  |  |
| 10 | Research on recognizing and diagnosing ADHD in mid-life (35-50) | 94.40 | 4.11 | 3.88-4.35 | 0.47 | 4.00 | 0.00 | 3.00 | 5.00 | 18 |
|  |  |  |  |  |  |  |  |  |  |  |
| 11 | Educating individuals with ADHD and their loved ones about medication management, different medications available, and best treatment options | 94.10 | 4.35 | 4.04-4.66 | 0.61 | 4.00 | 1.00 | 3.00 | 5.00 | 17 |
|  |  |  |  |  |  |  |  |  |  |  |
| 12 | Researching how different treatments affect different individuals in the short- and long-term using a person-centered approach to tailor treatments to all ADHD individuals | 94.10 | 4.29 | 3.99-4.60 | 0.59 | 4.00 | 1.00 | 3.00 | 5.00 | 17 |
|  |  |  |  |  |  |  |  |  |  |  |
| 13 | Identifying delays/barriers to assessment and treatment, and the impacts they may have on different systems | 88.90 | 4.17 | 3.86-4.47 | 0.62 | 4.00 | 0.75 | 3.00 | 5.00 | 18 |
|  |  |  |  |  |  |  |  |  |  |  |
| 14 | Increasing knowledge and awareness about the impact of inter-generational ADHD (many generations of ADHD within a family) | 88.90 | 4.17 | 3.86-4.47 | 0.62 | 4.00 | 0.75 | 3.00 | 5.00 | 18 |
|  |  |  |  |  |  |  |  |  |  |  |
| 15 | Providing individuals with ADHD with the tools, information, and strategies to self-advocate | 88.90 | 4.11 | 3.82-4.40 | 0.58 | 4.00 | 0.00 | 3.00 | 5.00 | 18 |
|  |  |  |  |  |  |  |  |  |  |  |
| 16 | Including people with lived experience in the process of research about ADHD | 88.20 | 4.18 | 3.85-4.50 | 0.64 | 4.00 | 1.00 | 3.00 | 5.00 | 17 |
|  |  |  |  |  |  |  |  |  |  |  |
| 17 | Research on the impact of ADHD medications on hormonal and reproductive health | 83.30 | 4.22 | 3.86-4.59 | 0.73 | 4.00 | 1.00 | 3.00 | 5.00 | 18 |
|  |  |  |  |  |  |  |  |  |  |  |
| 18 | Research to expand our knowledge of ADHD and co-occurring health-related conditions (e.g. sleep, eating, oral health, personal hygiene) | 83.30 | 4.17 | 3.82-4.52 | 0.71 | 4.00 | 1.00 | 3.00 | 5.00 | 18 |
|  |  |  |  |  |  |  |  |  |  |  |
| 19 | Increasing the availability of adapted supports within the school system (e.g. report cards specific to students with ADHD, ADHD-friendly learning and aftercare programs) | 83.30 | 4.06 | 3.66-4.45 | 0.80 | 4.00 | 0.75 | 2.00 | 5.00 | 18 |
|  |  |  |  |  |  |  |  |  |  |  |
| 20 | Research to understand the prevalence and unique experiences of people with ADHD and other mental health challenges | 83.30 | 4.00 | 3.62-4.38 | 0.77 | 4.00 | 0.00 | 2.00 | 5.00 | 18 |
|  |  |  |  |  |  |  |  |  |  |  |
| 21 | Increasing understanding of ADHD as a condition warranting recognition by government and educational systems | 82.40 | 4.24 | 3.85-4.62 | 0.75 | 4.00 | 1.00 | 3.00 | 5.00 | 17 |
|  |  |  |  |  |  |  |  |  |  |  |
| 22 | Research on best treatments for addictions within the context of ADHD (e.g. substances, gaming, gambling, and screens) | 82.40 | 4.18 | 3.80-4.55 | 0.73 | 4.00 | 1.00 | 3.00 | 5.00 | 17 |
|  |  |  |  |  |  |  |  |  |  |  |
| 23 | Expanding our understanding of ADHD in under-served or marginalized populations (e.g. ethnic minorities, queer and gender-diverse communities, and Indigenous groups) | 77.80 | 4.17 | 3.78-4.56 | 0.79 | 4.00 | 1.00 | 3.00 | 5.00 | 18 |
|  |  |  |  |  |  |  |  |  |  |  |
| 24 | Increasing the availability of social support networks for individuals, couples, and families with ADHD | 77.80 | 4.00 | 3.66-4.34 | 0.69 | 4.00 | 0.00 | 3.00 | 5.00 | 18 |
|  |  |  |  |  |  |  |  |  |  |  |
| 25 | Increase public awareness of the different ways ADHD can present (e.g. on a spectrum, with different symptom types, can be "masked") | 77.80 | 3.83 | 3.41-4.26 | 0.86 | 4.00 | 0.00 | 2.00 | 5.00 | 18 |
|  |  |  |  |  |  |  |  |  |  |  |
| 26 | Increasing awareness among the general public about treated versus untreated ADHD and its implications (e.g. through awareness campaigns, school presentations and healthcare presentations) | 72.20 | 3.78 | 3.41-4.14 | 0.73 | 4.00 | 0.75 | 2.00 | 5.00 | 18 |
|  |  |  |  |  |  |  |  |  |  |  |
| 27 | Providing access to holistic treatment options supported by multi-disciplinary teams (e.g. medication, nutrition, occupational therapy) embedded within systems like workplace and education | 70.60 | 3.76 | 3.27-4.26 | 0.97 | 4.00 | 1.00 | 1.00 | 5.00 | 17 |
|  |  |  |  |  |  |  |  |  |  |  |
| 28 | Providing basic general training in recognizing ADHD to all personnel who interact with youth in their line of work (e.g. police, dentists, social workers, corrections officers, educators) | 66.70 | 3.83 | 3.41-4.26 | 0.86 | 4.00 | 1.00 | 2.00 | 5.00 | 18 |
|  |  |  |  |  |  |  |  |  |  |  |
| 29 | Increasing job opportunities and workplace accommodations for all employees with ADHD | 66.70 | 3.67 | 3.33-4.01 | 0.69 | 4.00 | 1.00 | 2.00 | 5.00 | 18 |
|  |  |  |  |  |  |  |  |  |  |  |
| 30 | Continuing to work towards a better understanding of the causes of ADHD (e.g. genetics, hereditability, neurological mechanisms, risk factors) | 64.70 | 3.88 | 3.41-4.36 | 0.93 | 4.00 | 2.00 | 2.00 | 5.00 | 17 |
|  |  |  |  |  |  |  |  |  |  |  |
| 31 | Research on optimizing existing non-drug treatments (e.g. meditation and mindfulness, psychotherapy, physical activity, acupuncture) | 64.70 | 3.82 | 3.30-4.35 | 1.01 | 4.00 | 2.00 | 2.00 | 5.00 | 17 |
|  |  |  |  |  |  |  |  |  |  |  |
| 32 | Targeted research examining the stigmatization of ADHD (e.g. self-stigma, parental stigma, stigmatization in schools or classrooms) | 52.90 | 3.53 | 3.08-3.98 | 0.87 | 4.00 | 1.00 | 2.00 | 5.00 | 17 |
|  |  |  |  |  |  |  |  |  |  |  |
| 33 | Encouraging positive-directed research to better understand the unique strengths of those with ADHD | 41.20 | 3.29 | 2.73-3.86 | 1.10 | 3.00 | 1.00 | 1.00 | 5.00 | 17 |
|  |  |  |  |  |  |  |  |  |  |  |
| 34 | Redefining ADHD in a more positive/normative way as a facet of neurodiversity, to de-stigmatize and de-medicalize it (e.g. by changing ADHD terminology to remove words like disorder, disability) | 38.90 | 2.89 | 2.21-3.57 | 1.37 | 3.00 | 2.00 | 1.00 | 5.00 | 18 |
|  |  |  |  |  |  |  |  |  |  |  |

ADHD=Attention-Deficit/Hyperactivity Disorder; CI=Confidence Interval; IQR=Interquartile Range; Max=maximum Likert score; Mean=mean Likert score; Median=median Likert score; Min=minimum Likert score; N=number of healthcare professionals that responded; SD=Standard Deviation.

# S5F Table. Top ten priorities for psychologists

| **Order** | **Item** | **Percentage Agreement (%)** | **Mean** | **Round** |
| --- | --- | --- | --- | --- |
|  |  |  |  |  |
| 1 | Providing access to healthcare providers who are well-trained to recognize ADHD | 100.00 | 4.79 | 2 |
|  |  |  |  |  |
| 2 | Increasing knowledge and training about ADHD and associated stigmas among all healthcare and mental health professionals (e.g. family doctors, nurse practitioners, pharmacists, psychologists, counsellors) | 100.00 | 4.78 | 3 |
|  |  |  |  |  |
| 3 | Providing access to funded services for individuals with ADHD and their loved ones (e.g. healthcare coverage for psychological services, and/or affordable options) | 100.00 | 4.50 | 3 |
|  |  |  |  |  |
| 4 | Research on socio-emotional functioning in ADHD (e.g. self-esteem issues, ability to regulate emotions) and its impact on relationships | 100.00 | 4.44 | 3 |
|  |  |  |  |  |
| 5 | Providing access to ADHD services (e.g. CBT, coaching, skills-based training, employment programs) | 100.00 | 4.42 | 2 |
|  |  |  |  |  |
| 6 | Research on diagnosing ADHD in girls and women | 100.00 | 4.42 | 2 |
|  |  |  |  |  |
| 7 | Research on how co-existing experiences (e.g., depression, anxiety) should be considered when diagnosing ADHD | 100.00 | 4.42 | 2 |
|  |  |  |  |  |
| 8 | Providing access to resources and services to smaller and/or rural communities | 100.00 | 4.39 | 3 |
|  |  |  |  |  |
| 9 | Providing more accessible information and support to navigate the healthcare system and find appropriate services/personnel to assist and advocate for individuals with ADHD | 100.00 | 4.22 | 3 |
|  |  |  |  |  |
| 10 | Increasing knowledge about ADHD among teachers and educators | 94.70 | 4.63 | 2 |
|  |  |  |  |  |

ADHD=Attention-Deficit/Hyperactivity Disorder; CBT=Cognitive Behavioural Therapy; Mean=mean Likert score. Note items 5,6, and 7 are equally ranked 5^th^.

# S5G Table. Round 2 for psychotherapists (N=15)

| **Order** | **Item** | **Percentage Agreement (%)** | **Mean** | **95% CI** | **SD** | **Median** | **IQR** | **Min** | **Max** | **N** |
| --- | --- | --- | --- | --- | --- | --- | --- | --- | --- | --- |
|  |  |  |  |  |  |  |  |  |  |  |
| 1 | Providing access to healthcare providers who are well-trained to recognize ADHD | 100.00 | 4.80 | 4.57-5.03 | 0.41 | 5.00 | 0.00 | 4.00 | 5.00 | 15 |
|  |  |  |  |  |  |  |  |  |  |  |
| 2 | Research on diagnosing ADHD in girls and women | 100.00 | 4.60 | 4.32-4.88 | 0.51 | 5.00 | 1.00 | 4.00 | 5.00 | 15 |
|  |  |  |  |  |  |  |  |  |  |  |
| 3 | Providing access to ADHD services (e.g. CBT, coaching, skills-based training, employment programs) | 100.00 | 4.53 | 4.25-4.82 | 0.52 | 5.00 | 1.00 | 4.00 | 5.00 | 15 |
|  |  |  |  |  |  |  |  |  |  |  |
| 4 | Creating new tools to capture how ADHD impacts social relationships and emotion regulation | 100.00 | 4.50 | 4.20-4.80 | 0.52 | 4.50 | 1.00 | 4.00 | 5.00 | 14 |
|  |  |  |  |  |  |  |  |  |  |  |
| 5 | Increasing knowledge about ADHD among parents | 100.00 | 4.47 | 4.18-4.75 | 0.52 | 4.00 | 1.00 | 4.00 | 5.00 | 15 |
|  |  |  |  |  |  |  |  |  |  |  |
| 6 | Increasing knowledge about ADHD among teachers and educators | 93.30 | 4.47 | 4.11-4.82 | 0.64 | 5.00 | 1.00 | 3.00 | 5.00 | 15 |
|  |  |  |  |  |  |  |  |  |  |  |
| 7 | Research on how co-existing experiences (e.g. depression, anxiety) should be considered when diagnosing ADHD | 93.30 | 4.47 | 4.11-4.82 | 0.64 | 5.00 | 1.00 | 3.00 | 5.00 | 15 |
|  |  |  |  |  |  |  |  |  |  |  |
| 8 | Research on what should be included when diagnosing ADHD (e.g. cognitive assessment) | 92.90 | 4.36 | 3.99-4.72 | 0.63 | 4.00 | 1.00 | 3.00 | 5.00 | 14 |
|  |  |  |  |  |  |  |  |  |  |  |
| 9 | Research on the long-term consequences of untreated ADHD | 85.70 | 4.00 | 3.55-4.45 | 0.78 | 4.00 | 0.00 | 2.00 | 5.00 | 14 |
|  |  |  |  |  |  |  |  |  |  |  |
| 10 | Providing access to support for families (spouses, parents, siblings) | 80.00 | 4.00 | 3.53-4.47 | 0.85 | 4.00 | 0.50 | 2.00 | 5.00 | 15 |
|  |  |  |  |  |  |  |  |  |  |  |
| 11 | Research on how well treatment works, and how safe it is, for older adults (age 50+) | 80.00 | 3.87 | 3.46-4.28 | 0.74 | 4.00 | 0.00 | 2.00 | 5.00 | 15 |
|  |  |  |  |  |  |  |  |  |  |  |
| 12 | Research on how ADHD impacts families (parents, partners, siblings) | 78.60 | 4.07 | 3.54-4.60 | 0.92 | 4.00 | 1.00 | 2.00 | 5.00 | 14 |
|  |  |  |  |  |  |  |  |  |  |  |
| 13 | Research on the benefits of treatments, relative to their costs ("cost-benefit analysis") | 78.60 | 4.07 | 3.54-4.60 | 0.92 | 4.00 | 1.00 | 2.00 | 5.00 | 14 |
|  |  |  |  |  |  |  |  |  |  |  |
| 14 | Providing general funding for ADHD research | 78.60 | 4.00 | 3.49-4.51 | 0.88 | 4.00 | 0.75 | 2.00 | 5.00 | 14 |
|  |  |  |  |  |  |  |  |  |  |  |
| 15 | Increasing awareness about ADHD among employers and in workplaces | 73.30 | 3.93 | 3.44-4.42 | 0.88 | 4.00 | 1.00 | 2.00 | 5.00 | 15 |
|  |  |  |  |  |  |  |  |  |  |  |
| 16 | Increasing awareness about ADHD among the general public (e.g. through national campaigns) | 73.30 | 3.73 | 3.34-4.12 | 0.70 | 4.00 | 0.50 | 2.00 | 5.00 | 15 |
|  |  |  |  |  |  |  |  |  |  |  |
| 17 | Research on what it means to be “impaired” by symptoms of ADHD | 64.30 | 3.50 | 2.96-4.04 | 0.94 | 4.00 | 1.00 | 2.00 | 5.00 | 14 |
|  |  |  |  |  |  |  |  |  |  |  |
| 18 | Research on diagnosing ADHD in older adults (age 50+) | 60.00 | 3.80 | 3.20-4.40 | 1.08 | 4.00 | 2.00 | 2.00 | 5.00 | 15 |
|  |  |  |  |  |  |  |  |  |  |  |
| 19 | Research on new non-drug treatments | 57.10 | 3.36 | 2.78-3.94 | 1.01 | 4.00 | 1.75 | 2.00 | 5.00 | 14 |
|  |  |  |  |  |  |  |  |  |  |  |
| 20 | Research on how to improve treatment compliance (i.e. making sure people take their medication and/or follow their treatment plan) | 50.00 | 3.43 | 2.94-3.92 | 0.85 | 3.50 | 1.00 | 2.00 | 5.00 | 14 |
|  |  |  |  |  |  |  |  |  |  |  |
| 21 | Providing housing programs for people with ADHD | 14.30 | 2.64 | 2.16-3.13 | 0.84 | 3.00 | 1.00 | 1.00 | 4.00 | 14 |
|  |  |  |  |  |  |  |  |  |  |  |

ADHD=Attention-Deficit/Hyperactivity Disorder; CBT=Cognitive Behavioural Therapy; CI=Confidence Interval; IQR=Interquartile Range; Max=maximum Likert score; Mean=mean Likert score; Median=median Likert score; Min=minimum Likert score; N=number of healthcare professionals that responded; SD=Standard Deviation.

# S5H Table. Round 3 for psychotherapists (N=14)

| **Order** | **Item** | **Percentage Agreement (%)** | **Mean** | **95% CI** | **SD** | **Median** | **IQR** | **Min** | **Max** | **N** |
| --- | --- | --- | --- | --- | --- | --- | --- | --- | --- | --- |
|  |  |  |  |  |  |  |  |  |  |  |
| 1 | Increasing understanding of ADHD as a condition warranting recognition by government and educational systems | 100.00 | 4.77 | 4.50-5.03 | 0.44 | 5.00 | 0.00 | 4.00 | 5.00 | 13 |
|  |  |  |  |  |  |  |  |  |  |  |
| 2 | Providing access to funded services for individuals with ADHD and their loved ones (e.g. healthcare coverage for psychological services, and/or affordable options) | 100.00 | 4.71 | 4.44-4.98 | 0.47 | 5.00 | 0.75 | 4.00 | 5.00 | 14 |
|  |  |  |  |  |  |  |  |  |  |  |
| 3 | Increasing knowledge and training about ADHD and associated stigmas among all healthcare and mental health professionals (e.g. family doctors, nurse practitioners, pharmacists, psychologists, counsellors) | 100.00 | 4.71 | 4.44-4.98 | 0.47 | 5.00 | 0.75 | 4.00 | 5.00 | 14 |
|  |  |  |  |  |  |  |  |  |  |  |
| 4 | Educating personnel in the school systems on how to best support and teach individuals with ADHD | 100.00 | 4.71 | 4.44-4.98 | 0.47 | 5.00 | 0.75 | 4.00 | 5.00 | 14 |
|  |  |  |  |  |  |  |  |  |  |  |
| 5 | Research on best treatments for addictions within the context of ADHD (e.g. substances, gaming, gambling, and screens) | 100.00 | 4.62 | 4.31-4.92 | 0.51 | 5.00 | 1.00 | 4.00 | 5.00 | 13 |
|  |  |  |  |  |  |  |  |  |  |  |
| 6 | Providing more accessible information and support to navigate the healthcare system and find appropriate services/personnel to assist and advocate for individuals with ADHD | 100.00 | 4.57 | 4.27-4.87 | 0.51 | 5.00 | 1.00 | 4.00 | 5.00 | 14 |
|  |  |  |  |  |  |  |  |  |  |  |
| 7 | Research on socio-emotional functioning in ADHD (e.g. self-esteem issues, ability to regulate emotions) and its impact on relationships | 100.00 | 4.50 | 4.20-4.80 | 0.52 | 4.50 | 1.00 | 4.00 | 5.00 | 14 |
|  |  |  |  |  |  |  |  |  |  |  |
| 8 | Providing access to resources and services to smaller and/or rural communities | 100.00 | 4.43 | 4.13-4.73 | 0.51 | 4.00 | 1.00 | 4.00 | 5.00 | 14 |
|  |  |  |  |  |  |  |  |  |  |  |
| 9 | Research on the impact of hormones (e.g. hormonal fluctuations, hormone replacement therapy, or contraceptives) on ADHD symptoms, and their interactions with ADHD medications | 92.90 | 4.71 | 4.24-5.19 | 0.83 | 5.00 | 0.00 | 2.00 | 5.00 | 14 |
|  |  |  |  |  |  |  |  |  |  |  |
| 10 | Identifying delays/barriers to assessment and treatment, and the impacts they may have on different systems | 92.90 | 4.64 | 4.28-5.01 | 0.63 | 5.00 | 0.75 | 3.00 | 5.00 | 14 |
|  |  |  |  |  |  |  |  |  |  |  |
| 11 | Optimizing the assessment process through the use of validated tools to improve early diagnosis and diagnostic accuracy, and reduce misdiagnosis | 92.90 | 4.50 | 4.12-4.88 | 0.65 | 5.00 | 1.00 | 3.00 | 5.00 | 14 |
|  |  |  |  |  |  |  |  |  |  |  |
| 12 | Increasing awareness among the general public about treated versus untreated ADHD and its implications (e.g. through awareness campaigns, school presentations and healthcare presentations) | 92.90 | 4.29 | 3.81-4.76 | 0.83 | 4.00 | 1.00 | 2.00 | 5.00 | 14 |
|  |  |  |  |  |  |  |  |  |  |  |
| 13 | Research on recognizing and diagnosing ADHD in mid-life (35-50) | 92.90 | 4.29 | 3.81-4.76 | 0.83 | 4.00 | 1.00 | 2.00 | 5.00 | 14 |
|  |  |  |  |  |  |  |  |  |  |  |
| 14 | Providing individuals with ADHD with the tools, information, and strategies to self-advocate | 92.90 | 4.21 | 3.88-4.55 | 0.58 | 4.00 | 0.75 | 3.00 | 5.00 | 14 |
|  |  |  |  |  |  |  |  |  |  |  |
| 15 | Increasing the availability of adapted supports within the school system (e.g. report cards specific to students with ADHD, ADHD-friendly learning and aftercare programs) | 92.90 | 4.21 | 3.88-4.55 | 0.58 | 4.00 | 0.75 | 3.00 | 5.00 | 14 |
|  |  |  |  |  |  |  |  |  |  |  |
| 16 | Increasing general awareness of ADHD and its impacts in girls and women (e.g. among healthcare providers, across the lifespan, education, workplace) | 92.90 | 4.21 | 3.75-4.68 | 0.80 | 4.00 | 1.00 | 2.00 | 5.00 | 14 |
|  |  |  |  |  |  |  |  |  |  |  |
| 17 | Including people with lived experience in the process of research about ADHD | 92.30 | 4.31 | 3.93-4.69 | 0.63 | 4.00 | 1.00 | 3.00 | 5.00 | 13 |
|  |  |  |  |  |  |  |  |  |  |  |
| 18 | Providing access to holistic treatment options supported by multi-disciplinary teams (e.g. medication, nutrition, occupational therapy) embedded within systems like workplace and education | 92.30 | 4.23 | 3.87-4.59 | 0.60 | 4.00 | 1.00 | 3.00 | 5.00 | 13 |
|  |  |  |  |  |  |  |  |  |  |  |
| 19 | Research on the impact of ADHD medications on hormonal and reproductive health | 85.70 | 4.50 | 3.96-5.04 | 0.94 | 5.00 | 0.75 | 2.00 | 5.00 | 14 |
|  |  |  |  |  |  |  |  |  |  |  |
| 20 | Providing basic general training in recognizing ADHD to all personnel who interact with youth in their line of work (e.g. police, dentists, social workers, corrections officers, educators) | 85.70 | 4.07 | 3.59-4.55 | 0.83 | 4.00 | 0.75 | 2.00 | 5.00 | 14 |
|  |  |  |  |  |  |  |  |  |  |  |
| 21 | Research to expand our knowledge of ADHD and co-occurring health-related conditions (e.g. sleep, eating, oral health, personal hygiene) | 85.70 | 4.00 | 3.55-4.45 | 0.78 | 4.00 | 0.00 | 2.00 | 5.00 | 14 |
|  |  |  |  |  |  |  |  |  |  |  |
| 22 | Researching how different treatments affect different individuals in the short- and long-term using a person-centered approach to tailor treatments to all ADHD individuals | 84.60 | 4.23 | 3.79-4.67 | 0.73 | 4.00 | 1.00 | 3.00 | 5.00 | 13 |
|  |  |  |  |  |  |  |  |  |  |  |
| 23 | Educating individuals with ADHD and their loved ones about medication management, different medications available, and best treatment options | 84.60 | 4.15 | 3.61-4.70 | 0.90 | 4.00 | 1.00 | 2.00 | 5.00 | 13 |
|  |  |  |  |  |  |  |  |  |  |  |
| 24 | Research on optimizing existing non-drug treatments (e.g. meditation and mindfulness, psychotherapy, physical activity, acupuncture) | 84.60 | 4.08 | 3.69-4.46 | 0.64 | 4.00 | 0.00 | 3.00 | 5.00 | 13 |
|  |  |  |  |  |  |  |  |  |  |  |
| 25 | Increasing job opportunities and workplace accommodations for all employees with ADHD | 78.60 | 4.14 | 3.70-4.59 | 0.77 | 4.00 | 1.00 | 3.00 | 5.00 | 14 |
|  |  |  |  |  |  |  |  |  |  |  |
| 26 | Expanding our understanding of ADHD in under-served or marginalized populations (e.g. ethnic minorities, queer and gender-diverse communities, and Indigenous groups) | 78.60 | 4.07 | 3.54-4.60 | 0.92 | 4.00 | 1.00 | 2.00 | 5.00 | 14 |
|  |  |  |  |  |  |  |  |  |  |  |
| 27 | Research to understand the prevalence and unique experiences of people with ADHD and other mental health challenges | 78.60 | 4.07 | 3.65-4.49 | 0.73 | 4.00 | 0.75 | 3.00 | 5.00 | 14 |
|  |  |  |  |  |  |  |  |  |  |  |
| 28 | Increasing the availability of social support networks for individuals, couples, and families with ADHD | 78.60 | 4.00 | 3.61-4.39 | 0.68 | 4.00 | 0.00 | 3.00 | 5.00 | 14 |
|  |  |  |  |  |  |  |  |  |  |  |
| 29 | Increasing knowledge and awareness about the impact of inter-generational ADHD (many generations of ADHD within a family) | 78.60 | 3.86 | 3.31-4.41 | 0.95 | 4.00 | 0.00 | 2.00 | 5.00 | 14 |
|  |  |  |  |  |  |  |  |  |  |  |
| 30 | Encouraging positive-directed research to better understand the unique strengths of those with ADHD | 76.90 | 3.69 | 3.18-4.21 | 0.85 | 4.00 | 0.00 | 2.00 | 5.00 | 13 |
|  |  |  |  |  |  |  |  |  |  |  |
| 31 | Increase public awareness of the different ways ADHD can present (e.g. on a spectrum, with different symptom types, can be "masked") | 71.40 | 3.86 | 3.26-4.45 | 1.03 | 4.00 | 1.50 | 2.00 | 5.00 | 14 |
|  |  |  |  |  |  |  |  |  |  |  |
| 32 | Targeted research examining the stigmatization of ADHD (e.g. self-stigma, parental stigma, stigmatization in schools or classrooms) | 69.20 | 3.69 | 2.98-4.41 | 1.18 | 4.00 | 1.00 | 1.00 | 5.00 | 13 |
|  |  |  |  |  |  |  |  |  |  |  |
| 33 | Continuing to work towards a better understanding of the causes of ADHD (e.g. genetics, hereditability, neurological mechanisms, risk factors) | 69.20 | 3.62 | 3.22-4.01 | 0.65 | 4.00 | 1.00 | 2.00 | 4.00 | 13 |
|  |  |  |  |  |  |  |  |  |  |  |
| 34 | Redefining ADHD in a more positive/normative way as a facet of neurodiversity, to de-stigmatize and de-medicalize it (e.g. by changing ADHD terminology to remove words like disorder, disability) | 50.00 | 3.36 | 2.69-4.02 | 1.15 | 3.50 | 1.00 | 1.00 | 5.00 | 14 |
|  |  |  |  |  |  |  |  |  |  |  |

ADHD=Attention-Deficit/Hyperactivity Disorder; CI=Confidence Interval; IQR=Interquartile Range; Max=maximum Likert score; Mean=mean Likert score; Median=median Likert score; Min=minimum Likert score; N=number of healthcare professionals that responded; SD=Standard Deviation.

# S5I Table. Top ten priorities for psychotherapists

| **Order** | **Item** | **Percentage Agreement (%)** | **Mean** | **Round** |
| --- | --- | --- | --- | --- |
|  |  |  |  |  |
| 1 | Providing access to healthcare providers who are well-trained to recognize ADHD | 100.00 | 4.80 | 2 |
|  |  |  |  |  |
| 2 | Increasing understanding of ADHD as a condition warranting recognition by government and educational systems | 100.00 | 4.77 | 3 |
|  |  |  |  |  |
| 3 | Providing access to funded services for individuals with ADHD and their loved ones (e.g. healthcare coverage for psychological services, and/or affordable options) | 100.00 | 4.71 | 3 |
|  |  |  |  |  |
| 4 | Increasing knowledge and training about ADHD and associated stigmas among all healthcare and mental health professionals (e.g. family doctors, nurse practitioners, pharmacists, psychologists, counsellors) | 100.00 | 4.71 | 3 |
|  |  |  |  |  |
| 5 | Educating personnel in the school systems on how to best support and teach individuals with ADHD | 100.00 | 4.71 | 3 |
|  |  |  |  |  |
| 6 | Research on best treatments for addictions within the context of ADHD (e.g. substances, gaming, gambling, and screens) | 100.00 | 4.62 | 3 |
|  |  |  |  |  |
| 7 | Research on diagnosing ADHD in girls and women | 100.00 | 4.60 | 2 |
|  |  |  |  |  |
| 8 | Providing more accessible information and support to navigate the healthcare system and find appropriate services/personnel to assist and advocate for individuals with ADHD | 100.00 | 4.57 | 3 |
|  |  |  |  |  |
| 9 | Providing access to ADHD services (e.g. CBT, coaching, skills-based training, employment programs) | 100.00 | 4.53 | 2 |
|  |  |  |  |  |
| 10 | Creating new tools to capture how ADHD impacts social relationships and emotion regulation | 100.00 | 4.50 | 2 |
|  |  |  |  |  |
| 11 | Research on socio-emotional functioning in ADHD (e.g. self-esteem issues, ability to regulate emotions) and its impact on relationships | 100.00 | 4.50 | 3 |
|  |  |  |  |  |

ADHD=Attention-Deficit/Hyperactivity Disorder; CBT=Cognitive Behavioural Therapy; Mean=mean Likert score. Note that items 2,3,4 and 5 are equally ranked as 2^nd^ and items 10 and 11 are both equally ranked.
