# Supplementary material for: Top ten priorities identified by healthcare professionals to support the clinical care of individuals with attention-deficit/hyperactivity disorder: A Canadian Delphi study
Source: PLoS One. 2025 Dec 19;20(12):e0339378. doi: 10.1371/journal.pone.0339378 (PMC12716771; doi:10.1371/journal.pone.0339378)
Supplement: S1 Appendix — (DOCX) [file pone.0339378.s006.docx]

**S1 Appendix: Derivation of 34 new items**

Table of Contents

S1A. Flowchart for the [derivation of items 2](#_Toc213166062)

[S1B. 34 new items 4](#_Toc213166063)

[S1C. Items dropped as similar to 21 predetermined items 7](#_Toc213166064)

[S1D. Items dropped due to <10 respondents 8](#_Toc213166065)

# **S1A. Flowchart for the derivation of items**

**1015** Participants provided free-text responses in Round 1

**28** Items excluded:

**6** Similar to 21 predetermined items

**22** Had <10 respondents

**715** Items excluded:
**152** Vague or

uninterpretable
**563** Duplicates of 21

predetermined items

**1084** Items excluded:

**1084** Duplicate items

**62** Priority items

**34** New items

**1956** Total free-text items

**1241** Items assessed for eligibility

**Identification**

**Screening**

**157** Items reviewed for similarity

and consolidated through team consensus

**Inclusion**

Note: A total of 1015 free-text responses were received from survey participants. Two trained raters each reviewed roughly half of the free-text responses to determine whether they: (i) were too vague, (ii) duplicated one of the 21 predetermined items, or (iii) represented a potential new item. Following review of the free-text responses, there were a total of 1956 items identified because some participants entered at least one item. The classifications performed by the two raters were then reviewed and validated by two researchers (BLC and EAC). The 157 items in category (iii) were discussed in a team meeting with the two researchers and the raters. Among the 157 items, those that were very similar to each other were grouped into a single item during a process of discussion and consensus resulting in 62 priority items. These 62 items were brought to the attention of the entire research team for review and discussion to determine the feasibility of including all 62 items in a survey. The team concluded that 62 items would be too burdensome for participants, so a team decision was made to drop the 28 items that had been suggested by fewer than 10 respondents (corresponding to <1% of all free-text suggestions provided). Although the cut-off of 10 was chosen arbitrarily, the research team used a process of discussion and consensus to determine the final 34 new items to ensure a good balance between capturing a broad range of participant suggestions while minimizing survey burden.

# **S1B. 34 new items**

| **Number** | **Item** | **Number of Respondents** |
| --- | --- | --- |
|  |  |  |
| 1 | Increasing knowledge and training about ADHD and associated stigmas among all healthcare and mental health professionals (e.g. family doctors, nurse practitioners, pharmacists, psychologists, counsellors) | 100 |
|  |  |  |
| 2 | Providing access to funded services for individuals with ADHD and their loved ones (e.g. healthcare coverage for psychological services, and/or affordable options) | 99 |
|  |  |  |
| 3 | Providing more accessible information and support to navigate the healthcare system and find appropriate services/personnel to assist individuals with ADHD | 73 |
|  |  |  |
| 4 | Optimizing the assessment process through the use of validated tools to improve early diagnosis and diagnostic accuracy, and reduce misdiagnosis | 49 |
|  |  |  |
| 5 | Increasing general awareness of ADHD and its impacts in girls and women (e.g. among healthcare providers, across the lifespan, education, workplace) | 49 |
|  |  |  |
| 6 | Redefining ADHD in a more positive/normative way as a facet of neurodiversity, to de-stigmatize and de-medicalize it (e.g. by changing ADHD terminology to remove words like disorder, disability) | 41 |
|  |  |  |
| 7 | Research on the impact of hormones (e.g. hormonal fluctuations, hormone replacement therapy or contraceptives) on ADHD symptoms, and their interactions with ADHD medications | 39 |
|  |  |  |
| 8 | Increasing the availability of social support networks for individuals, couples, and families with ADHD | 34 |
|  |  |  |
| 9 | Increasing the availability of adapted supports within the school system (e.g. report cards specific to students with ADHD, ADHD-friendly learning and aftercare programs) | 33 |
|  |  |  |
| 10 | Expanding our understanding of ADHD in under-served or marginalized populations (e.g. ethnic minorities, queer and gender-diverse communities, and Indigenous groups) | 33 |
|  |  |  |
| 11 | Research to understand the prevalence and unique experiences of people with ADHD and other mental health challenges | 33 |
|  |  |  |
| 12 | Providing individuals with ADHD with the tools, information, and strategies to self-advocate | 30 |
|  |  |  |
| 13 | Continuing to work towards a better understanding of the causes of ADHD (e.g. genetics, hereditability, neurological mechanisms, risk factors) | 30 |
|  |  |  |
| 14 | Increase public awareness of the different ways ADHD can present (e.g. on a spectrum, with different symptom types, can be “masked”) | 29 |
|  |  |  |
| 15 | Providing basic general training in recognizing ADHD to all personnel who interact with youth in their line of work (e.g. police, dentists, social workers, corrections officers, educators) | 24 |
|  |  |  |
| 16 | Encouraging positive-directed research to better understand the unique strengths of those with ADHD | 24 |
|  |  |  |
| 17 | Increasing job opportunities and workplace accommodations for all employees with ADHD | 23 |
|  |  |  |
| 18 | Increasing awareness among the general public about treated versus untreated ADHD and its implications (e.g. through awareness campaigns, school presentations and healthcare presentations) | 22 |
|  |  |  |
| 19 | Research on best treatments for addictions within the context of ADHD (e.g. substances, gaming, gambling, and screens) | 21 |
|  |  |  |
| 20 | Research on socio-emotional functioning in ADHD (e.g. self-esteem issues, ability to regulate emotions) and its impact on relationships | 20 |
|  |  |  |
| 21 | Providing access to holistic treatment options supported by multi-disciplinary teams (e.g. medication, nutrition, occupational therapy) embedded within systems like workplace and education | 20 |
|  |  |  |
| 22 | Educating personnel in the school systems on how to best support and teach individuals with ADHD | 17 |
|  |  |  |
| 23 | Research to expand our knowledge of ADHD and co-occurring health-related conditions (e.g. sleep, eating, oral health, personal hygiene) | 17 |
|  |  |  |
| 24 | Increasing knowledge and awareness about the impact of inter-generational ADHD (many generations of ADHD within a family) | 16 |
|  |  |  |
| 25 | Including people with lived experience in the process of research about ADHD | 16 |
|  |  |  |
| 26 | Researching how different treatments affect different individuals in the short- and long-term, using a person-centered approach to tailor treatments to all ADHD individuals | 15 |
|  |  |  |
| 27 | Educating individuals with ADHD and their loved ones about medication management, different medications available, and best treatment options | 14 |
|  |  |  |
| 28 | Targeted research examining the stigmatization of ADHD (e.g. self-stigma, parental stigma, stigmatization in schools or classrooms) | 14 |
|  |  |  |
| 29 | Identifying delays/barriers to assessment and treatment, and the impacts they may have on different systems | 13 |
|  |  |  |
| 30 | Research on the impact of ADHD medications on hormonal fluctuations and reproductive health | 13 |
|  |  |  |
| 31 | Research on optimizing existing non-drug treatments (e.g. meditation and mindfulness, psychotherapy, physical activity, acupuncture) | 13 |
|  |  |  |
| 32 | Providing access to resources and services to smaller and/or rural communities | 12 |
|  |  |  |
| 33 | Research on recognizing and diagnosing ADHD in mid-life (ages 35-50) | 12 |
|  |  |  |
| 34 | Increasing understanding of ADHD as a condition warranting recognition by government and educational systems | 12 |
|  |  |  |

ADHD=Attention-Deficit/Hyperactivity Disorder

# **S1C. Items dropped as similar to 21 predetermined items**

| **Number** | **Item** | **Number of Respondents** |
| --- | --- | --- |
|  |  |  |
| 1 | Research on best treatments for ADHD with co-occurring challenges (e.g. other mental health concerns, intellectual disabilities, autism) | 56 |
|  | *→ Embedded within Item 26 above* |  |
|  |  |  |
| 2 | Research on factors that impact treatment compliance and medication adherence (e.g. related to fear/stigma, access to medication) | 22 |
|  | *→ Dropped because it was similar one of the Initial 21 predetermined Items (“Research on how to improve treatment compliance (i.e., making sure people take their medication and/or follow their treatment plan)”)* |  |
|  |  |  |
| 3 | Providing training to parents and families on advocacy, navigating systems, and supporting loved ones with ADHD | 20 |
|  | *→ Embedded within Items 3 and 12 above* |  |
|  |  |  |
| 4 | Consider alternative terminology to refer to ADHD (e.g. remove words like disorder, disability) | 17 |
|  | *→ Embedded within Item 6 above* |  |
|  |  |  |
| 5 | Increasing the availability of evidence-based interventions within different systems, including workplace and educational settings | 11 |
|  | *→ Embedded within Item 21 above* |  |
|  |  |  |
| 6 | Increasing knowledge and training about ADHD and associated stigmas among all mental health professionals (e.g. psychologists, therapists, counsellors) | 11 |
|  | *→ Embedded within Item 1 above* |  |
|  |  |  |

ADHD=Attention-Deficit/Hyperactivity Disorder.

# **S1D. Items dropped due to <10 respondents**

| **Number** | **Item** | **Number of Respondents** |
| --- | --- | --- |
|  |  |  |
| 1 | Increased research on ADHD and gender (e.g. gender differences, gender roles, gender norms, stigma, societal expectations) | 9 |
|  |  |  |
| 2 | Increasing supports for girls and women with ADHD | 7 |
|  |  |  |
| 3 | Research on ADHD and the relationship with dementia, Alzheimer’s and/or or other neurodegenerative disorders | 7 |
|  |  |  |
| 4 | Increasing access to medications | 7 |
|  |  |  |
| 5 | Create and provide access to integrated treatment for families with multiple ADHD diagnoses | 7 |
|  |  |  |
| 6 | Increasing knowledge about ADHD among personnel in justice institutions (e.g. police, lawyers and legal aid, judiciary and corrections) | 6 |
|  |  |  |
| 7 | Providing access to ADHD services for older adults (e.g. specialized seniors’ homes, social networks) | 5 |
|  |  |  |
| 8 | Research on how ADHD medications interact with other medical factors (e.g. genetic predispositions, pre-existing health conditions, other prescription drugs) | 5 |
|  |  |  |
| 9 | Research on prevalence of individuals with ADHD within the criminal justice system | 4 |
|  |  |  |
| 10 | Research on how executive functioning relates to ADHD | 4 |
|  |  |  |
| 11 | Research on biometric tools for diagnosing ADHD, such as brain scans (e.g. MRI or EEG) or biofeedback | 3 |
|  |  |  |
| 12 | Providing institutional funding to support individuals with ADHD (e.g. in schools, in healthcare) | 2 |
|  |  |  |
| 13 | Research on the impact of ADHD on families with different incomes, and from different socioeconomic backgrounds | 2 |
|  |  |  |
| 14 | Research on ADHD and driving behaviour | 2 |
|  |  |  |
| 15 | Research on the co-occurrence of ADHD and autism in girls and women | 1 |
|  |  |  |
| 16 | Disability justice framed assessments | 1 |
|  |  |  |
| 17 | Research on new drug treatments to alleviate symptoms of ADHD (e.g. marijuana or psilocybin) | 1 |
|  |  |  |
| 18 | Demonstrating impact of impairment and outlining supports | 1 |
|  |  |  |
| 19 | Research ADHD and its impacts on one’s education | 1 |
|  |  |  |
| 20 | Research on implications of community-based programs | 1 |
|  |  |  |
| 21 | Research on ADHD and vulnerability to victimization | 1 |
|  |  |  |
| 22 | Research on ways to engage and stimulate individuals with ADHD | 1 |
|  |  |  |

ADHD=Attention-Deficit/Hyperactivity Disorder; EEG=electroencephalogram; MRI=magnetic resonance imaging.
