# Supplementary material for: Top ten priorities identified by healthcare professionals to support the clinical care of individuals with attention-deficit/hyperactivity disorder: A Canadian Delphi study
Source: PLoS One. 2025 Dec 19;20(12):e0339378. doi: 10.1371/journal.pone.0339378 (PMC12716771; doi:10.1371/journal.pone.0339378)
Supplement: S1 Survey — (DOCX) [file pone.0339378.s009.docx]

S1 Survey. All surveys pertaining to healthcare professionals

Table of Contents

[**Survey Round 1. English version 2**](#_Toc212537823)

[**Survey Round 1. French version 13**](#_Toc212537824)

[**Survey Round 2. English version 24**](#_Toc212537825)

[**Survey Round 2. French version 50**](#_Toc212537826)

[**Survey Round 3. English version 80**](#_Toc212537827)

[**Survey Round 3. French version 103**](#_Toc212537828)

# Survey Round 1. English version

Unravelling the Puzzles in ADHD: A Delphi Study (Wave 1)

Start of Block: CONSENT

Consent Form
**Name of Researcher, Faculty, Department, Telephone & Email**: XX

**Co-investigators:** XX

**Title of Project:** Unravelling the Puzzles in ADHD: Identifying Research Challenges and Opportunities across Canada Using a Delphi Approach
   This consent form, which you are encouraged to print for your records, is only part of the process of informed consent. If you want more details about something mentioned here, or information not included here, you should feel free to ask. Please take the time to read this carefully and to understand any accompanying information. The Research Ethics Board has approved this research study (REB22-0011). Participation is completely voluntary, and confidential.  
 **Purpose of the Study** The purpose of this study is to integrate and advance perspectives on topics and themes that should be prioritized in Canadian research on attention-deficit/hyperactivity disorder (ADHD). To this end, we would like to solicit your views, as a professional, researcher, or trainee involved and/or interested in ADHD, or as someone who has/supports someone with ADHD on what is most important to address to better serve our ADHD community. In this study, we are using a Delphi technique, which is a repetitive process that first aims to gather a broad range of opinions and views, which are then summarized to provide the basis for further discussion and feedback in subsequent surveys. The goal is to achieve general consensus on important ADHD-related priorities by the end of the final survey.  
 **What Will I Be Asked To Do?** If you decide to participate in this research, you will be asked to complete a brief survey (5 to 10 minutes), asking you about your views on themes and issues that should be prioritized for future research or funding in ADHD. Your results will be integrated with those of other participants, and you will then be recontacted via email in a few months to complete a second brief survey. We anticipate that consensus will be reached after the second survey, but it is possible we may reach out to you again via email with a third brief survey a few months following the second survey, if we have not yet reached consensus. Participation in the follow up surveys is completely voluntary. The content of the surveys will consist of gaps, challenges and opportunities related to ADHD, which you will be asked to rank in terms of importance (e.g., “How important do you feel it is to increase awareness about ADHD in post-secondary settings?”). Participation is completely voluntary. You may refuse to participate altogether, may refuse to participate in parts of the study, may decline to answer any and all questions, and may withdraw from the study without penalty or adverse consequence.  
 **What Type of Personal Information Will Be Collected?** Should you agree to participate, you will be asked to provide some information about your background (e.g., whether you are a researcher, clinician, educator, trainee, or person with lived ADHD experience) and general demographic location (e.g., province or country of residence) so that we can get a general sense of the make-up of the participant sample providing feedback. This is important because some issues will be relatively more important to educators than to clinicians, for example, and having this background information on participants will allow us to contextualize our findings. Your name and email address will also be retained to contact you for follow-up surveys.  
**Are there Risks or Benefits if I Participate?** There are no risks to you in participating. Those who choose to participate will be entered in a draw to win one of ten $50 gift cards.  
**What Happens to the Information I Provide?** Your data will be stored in an encrypted, password-protected file on a secure server at the University. Only members of the research team will have access to the results you provide, and XX will remove any identifying information (name, email address) from the data file before giving access to the co-investigators. You will be free to withdraw your responses from the survey until 2 weeks after the first survey closes. (If you wish to withdraw, please contact XX). After this time, withdrawal will no longer be possible, because your responses will be integrated with those of other participants and will form the basis for the next round of surveys. Only group information will be summarized for any presentation or publication of results. It is possible that the de-identified anonymized datafile will be uploaded to an online data repository. Data repositories can be used to share de-identified and non-confidential data only. Contributors are required to remove, replace, or redact such information from datasets prior to upload. The researchers acknowledge that the host of the online survey (Qualtrics) may automatically collect participant data without their knowledge (i.e., IP addresses.) Although this information may be provided or made accessible to the researchers, it will not be used or saved without participant’s consent on the researchers’ system. Further, because this project employs e-based collection techniques, data may be subject to access by third parties as a result of various security legislation now in place in many countries and thus the confidentiality and privacy of data cannot be guaranteed during web-based transmission.
**Signatures**  By clicking “I consent to participate in this research study” below, you are indicating that 1) you understand to your satisfaction the information provided to you about your participation in this research project, and 2) you agree to participate in the research project. In no way does this waive your legal rights nor release the investigators, sponsors, or involved institutions from their legal and professional responsibilities. You are free to withdraw from this research project, and should feel free to ask for clarification or new information throughout your participation.

**Questions/Concerns** If you have any further questions or want clarification regarding this research and/or your participation, please contact: XX. You are encouraged to save or print a copy of this consent form for your records and reference. The investigator also has a copy of this consent form.

Obtain Consent

- I consent to participate in this research study
- I do not consent to participate in this research study

Skip To: End of Survey If = I do not consent to participate in this research study

| Page Break |  |
| --- | --- |

Bot Check Disclaimer We will be asking you some questions about your personal background and your perceptions of ADHD priorities, along with other unrelated questions to ensure that respondents (not bots) are answering and reading the questions thoroughly.

End of Block: CONSENT

Start of Block: DEMOGRAPHICS

Province What Canadian province or territory do you reside in?

- Alberta
- British Columbia
- Manitoba
- New Brunswick
- Newfoundland and Labrador
- Northwest Territories
- Nova Scotia
- Nunavut
- Ontario
- Prince Edward Island
- Québec
- Saskatchewan
- Yukon
- I do not live in Canada

Skip To: End of Survey If What Canadian province or territory do you reside in? = I do not live in Canada

Role What is your primary role with regard to ADHD?

- I am an ADHD researcher
- I am a clinical practitioner serving clientele with ADHD
- I am an educator working with individuals with ADHD
- I am a student/trainee with plans to continue in research on ADHD
- I am a student/trainee with plans to continue as a clinician
- I am a student/trainee with plans to continue as an educator
- I have ADHD
- I am the parent/guardian or partner of a loved one living with ADHD
- Other (please specify)

OtherRole If 'other', please specify:

________________________________________________________________

Display this question:

If What is your primary role with regard to ADHD? = I am a clinical practitioner serving clientele with ADHD

Or What is your primary role with regard to ADHD? = I am a student/trainee with plans to continue in research on ADHD

Discipline What is your primary discipline?

- Adult Psychiatry
- Adult Psychology
- Child & Adolescent Psychiatry
- Child & Adolescent Psychology
- Psychotherapy
- Family Medicine
- Pediatrics
- Nursing
- Other (please specify)

Display this question:

If What is your primary role with regard to ADHD? = I am a clinical practitioner serving clientele with ADHD

Or What is your primary role with regard to ADHD? = I am a student/trainee with plans to continue in research on ADHD

OtherDiscipline If 'other', please specify:

________________________________________________________________

Attention Check1 I live on planet Earth.

- Yes
- No

| Page Break |  |
| --- | --- |

Age What is your age range?

- 16-24 years
- 25-39 years
- 40-59 years
- 60+ years

Gender What gender do you identify as?

- Man
- Woman
- Other
- Prefer not to say

End of Block: DEMOGRAPHICS

Start of Block: ASSESSMENT

AttentionCheck2 'B' is the first letter of the alphabet

- Yes
- No

Start of Block: PRIORITIES

Instruction We would like to hear your opinions about the types of ADHD-related themes and issues that should be prioritized presently in Canada. Please provide your perspective about the following topics for future research and training.

Socialchallenges **Social challenges** Which of these social challenges do you think are important to address? Please indicate what you think is important, not what you believe would necessarily benefit society at the general level.

|  | Not a priority (1) | Low priority (2) | Undecided (3) | High priority (4) | Critical priority (5) |
| --- | --- | --- | --- | --- | --- |
| Providing access to healthcare providers who are well-trained to recognize ADHD |  |  |  |  |  |
| Providing access to ADHD services (CBT, coaching, skills-based training, employment programs, etc.) |  |  |  |  |  |
| Providing access to support for families (spouses, parents, siblings) |  |  |  |  |  |
| Providing general funding for ADHD research |  |  |  |  |  |
| Providing housing programs for people with ADHD |  |  |  |  |  |

SocialClarification Please provide any clarification to your above ratings, if needed:

________________________________________________________________

KTchallenges **Challenges related to knowledge and awareness of ADHD** Which of these knowledge/awareness challenges do you think are important to address? Please indicate what you think is important, not what you believe would necessarily benefit society at the general level.

|  | Not a priority (1) | Low priority (2) | Undecided (3) | High priority (4) | Critical priority (5) |
| --- | --- | --- | --- | --- | --- |
| Increasing knowledge about ADHD among teachers and educators |  |  |  |  |  |
| Increasing knowledge about ADHD among parents |  |  |  |  |  |
| Increasing awareness about ADHD among employers and in workplaces |  |  |  |  |  |
| Increasing awareness about ADHD among the general public (e.g., through national campaigns) |  |  |  |  |  |

KTClarification Please provide any clarification to your above ratings, if needed:

________________________________________________________________

| Page Break |  |
| --- | --- |

Attention Check3 The Earth is flat.

- Yes
- No

DXchallenges **Challenges related to diagnosing ADHD** Which of these diagnosis challenges do you think are important to address? Please indicate what you think is important, not what you believe would necessarily benefit society at the general level.

|  | Not a priority (1) | Low priority (2) | Undecided (3) | High priority (4) | Critical priority (5) |
| --- | --- | --- | --- | --- | --- |
| Research on diagnosing ADHD in girls and women |  |  |  |  |  |
| Research on diagnosing ADHD in older adults (age 50+) |  |  |  |  |  |
| Research on how co-existing experiences (e.g., depression, anxiety) should be considered when diagnosing ADHD |  |  |  |  |  |
| Research on what should be included when diagnosing ADHD (e.g., cognitive assessment) |  |  |  |  |  |
| Creating new tools to capture how ADHD impacts social relationships and emotion regulation |  |  |  |  |  |
| Research on what it means to be "impaired" by symptoms of ADHD |  |  |  |  |  |

DxClarification Please provide any clarification to your above ratings, if needed:

________________________________________________________________

TXchallenges **Challenges related to treating ADHD** Which of these treatment challenges do you think are important to address? Please indicate what you think is important, not what you believe would necessarily benefit society at the general level.

|  | Not a priority (1) | Low priority (2) | Undecided (3) | High priority (4) | Critical priority (5) |
| --- | --- | --- | --- | --- | --- |
| Research on new non-drug treatments |  |  |  |  |  |
| Research on how ADHD impacts families (parents, partners, siblings) |  |  |  |  |  |
| Research on the long-term consequences of untreated ADHD |  |  |  |  |  |
| Research on how well treatment works, and how safe it is, for older adults (age 50+) |  |  |  |  |  |
| Research on how to improve treatment compliance (i.e., making sure people take their medication and/or follow their treatment program) |  |  |  |  |  |
| Research on the benefits of treatments, relative to their costs ('cost-benefit analysis') |  |  |  |  |  |

TxClarification Please provide any clarification to your above ratings, if needed:

________________________________________________________________

FreeTextSuggestions Please provide any **additional** suggestions of ADHD-related priorities you think may be important to consider.

________________________________________________________________

End of Block: PRIORITIES

Start of Block: CONTACT

Giftcard Draw Please enter your email address below, if you wish to be entered in a draw to win one of ten $50 gift cards.

________________________________________________________________

Follow-up We would like to contact you in a few months from now, to ask you a few more very brief questions to refine our list of ADHD-related priorities. If you participate, you will be entered in another gift card draw. If you accept to be contacted, please enter your email address.

________________________________________________________________

End of Block: CONTACT

Start of Block: EndBlock

**Thank you for your responses.**

We will integrate your responses with those of other participants, and re-contact you again shortly to finalize this priority list and hone in on areas of agreement.

If you have any additional feedback, please enter it here.

________________________________________________________________

End of Block: EndBlock

# Survey Round 1. French version

Unravelling the Puzzles in ADHD: A Delphi Study (Wave 1)

Start of Block: CONSENT

Nom de la chercheure, faculté, département, téléphone et courriel : XX

**Co-chercheures :** XX

**Titre du projet :** Démêler les énigmes du TDAH avec la méthode Delphi : défis et opportunités de recherche à travers le Canada
   Ce formulaire de consentement, que nous vous encourageons à imprimer pour vos dossiers, n'est qu'une partie du processus de consentement éclairé. Si vous voulez plus de détails sur le contenu de ce formulaire ou des informations non incluses ici, n'hésitez pas à nous le demander. Veuillez prendre le temps de le lire attentivement et de bien en comprendre le contenu. Cette étude a été approuvée par le comité d'éthique de la recherche (REB22-0011). Votre participation est entièrement volontaire et confidentielle. **But de l'étude**Le but de cette étude est d'établir un consensus sur les sujets et thèmes qui devraient être prioritaires dans la recherche canadienne sur le trouble déficitaire de l'attention/hyperactivité (TDAH). À cette fin, nous aimerions solliciter votre point de vue, en tant que professionnel(le), chercheur(e) ou étudiant(e) intéressé(e) par le TDAH, sur les points qui seront importants à prioriser pour mieux servir notre communauté. Dans cette étude, nous utilisons la méthode Delphi, un processus itératif qui vise d'abord à recueillir un grand nombre d'opinions et de points de vue, qui sont ensuite résumés pour guider des discussions ultérieures. L'objectif, à la fin de l'enquête finale est d'établir un consensus général sur les priorités de recherche importantes liées au TDAH.
 **Que devrai-je faire?** Nous vous demanderons de participer à un bref sondage (5 à 10 minutes) sollicitant votre point de vue sur les thèmes qui devraient être prioritaires pour les recherches futures sur le TDAH. Vos réponses seront agrégées avec celles d'autres participants, et nous vous recontacterons par courriel dans quelques mois afin de compléter un deuxième bref sondage. Nous prévoyons atteindre un consensus après ce deuxième sondage, mais il est possible que nous vous recontactions par courriel pour un troisième sondage si nous ne sommes toujours pas parvenus à un consensus. Votre participation est entièrement volontaire et vous aurez la possibilité de refuser de répondre à chaque sondage. Dans chaque sondage, vous serez appelé(e) à évaluer l'importance de différents défis liés au TDAH (p.ex., « Dans quelle mesure pensez-vous qu'il est important de prioriser la sensibilisation au TDAH dans les milieux postsecondaires? » ). Votre participation est entièrement volontaire. Vous pouvez refuser de participer entièrement, ou choisir de répondre à seulement certaines parties du sondage, et pouvez vous retirer de l'étude sans pénalité ou conséquence négative.  
 **Quel type d'informations personnelles seront recueillies?** Nous vous demanderons de fournir certaines informations sur votre parcours professionnel (p.ex., si vous œuvrez dans le domaine de la recherche, de l'éducation, de la santé, etc.) ainsi que certaines informations démographiques (p.ex., votre province ou pays de résidence) afin d'avoir une idée générale de la composition de notre échantillon de participants. Ceci est important puisque nous anticipons que certaines questions auront une plus grande importance pour, par exemple, les éducateurs/éducatrices que pour les médecins, et donc obtenir ces informations nous permettra de contextualiser nos résultats. Votre nom et adresse courriel seront également retenus pour vous recontacter dans le cadre des sondages de suivi.   
 **Y a-t-il des risques ou avantages si je participe?**Non, il n'y a aucun risque à participer. Ceux et celles qui choisiront de participer seront inscrits à un tirage pour gagner l'une de dix cartes-cadeaux d'une valeur de 50$.
**Que deviennent les informations que je fournis?** Vos données seront retenues dans un fichier crypté et protégé par un mot de passe sur un serveur sécurité. Seuls les membres de l'équipe de recherche auront accès aux réponses que vous fournissez, et XX supprimera toute information pouvant vous identifier (votre nom, adresse courriel) du fichier de données avant de la transmettre aux co-chercheures. Vous serez libre de retirer vos réponses au sondage jusqu'à deux semaines suivant la clôture du premier sondage. Après cette date, il ne sera plus possible de vous retirer, car vos réponses seront intégrées à celles des autres participants et constitueront la base du prochain sondage. Seuls des résultats agrégés feront l'objet de présentations publiques ou publications scientifiques. Il est possible que les données anonymisées dépersonnalisées soient téléchargées dans un référentiel de données en ligne. Les référentiels de données peuvent être utilisés pour partager uniquement des données anonymisées et non-confidentielles. Les contributeurs sont tenus de supprimer, de remplacer ou de censurer toute information confidentielle de la base de données avant le téléchargement.   Les chercheures reconnaissent que l'hébergeur de l'enquête en ligne (Qualtrics) peut recueillir automatiquement curtained données à l’insu des participants (c'est-à-dire leur adresse IP). Bien que ces informations puissent être fournies ou rendues accessibles aux chercheures, elles ne seront pas utilisées ou sauvegardées sans le consentement du participant sur le système des chercheures. En outre, puisque ce projet utilise des techniques de collecte en ligne, les données peuvent faire l'objet d'un accès par des tiers en raison de diverses législations sur la sécurité actuellement en place dans de nombreux pays et, par conséquent, la confidentialité des données ne peut être garantie lors de la transmission en ligne.
   **Signatures**  En cliquant sur « Je donne mon consentement à participer à cette étude de recherche » ci-dessous, vous indiquez que 1) vous comprenez les informations qui vous ont été fournies ci-dessus, et 2) vous acceptez de participer au projet de recherche. Cela ne renonce en aucun cas à vos droits légaux ni ne libère les enquêteurs ou les institutions impliquées de leurs responsabilités légales et professionnelles. Vous êtes libre de vous retirer de cette étude, et n'hésitez pas à demander des informations supplémentaires au besoin.   **Questions** Si vous avez des questions ou voulez obtenir des informations supplémentaires concernant l'étude et/ou votre participation, veuillez contacter XX. Si vous avez des inquiétudes quant à la manière dont vous avez été traité en tant que participant, veuillez contacter l'analyste en éthique de la recherche au Bureau des services de recherche XX ou envoyez un courriel à XX. Nous vous encourageons à conserver une copie de ce formulaire de consentement pour vos dossiers et à titre de référence. L'équipe de recherche détient également une copie de ce formulaire.

Obtain Consent

Je donne mon consentement à participer à cette étude

Je ne donne pas mon consentement à participer à cette étude

Skip To: End of Survey If = Je ne donne pas mon consentement à participer à cette étude

| Page Break |  |
| --- | --- |

BotCheck Disclaimer Nous allons vous poser des questions sur votre profil démographique et sur vos perceptions de priorités liées au TDAH, ainsi que d'autres questions pour nous assurer que de réels participants (et non des robots) répondent et lisent attentivement les questions.

End of Block: CONSENT

Start of Block: DEMOGRAPHICS

Province Dans quelle province candienne résidez-vous?

Alberta

Colombie-Britannique

Île-du-Prince-Édouard

Manitoba

Nouveau-Brunswick

Nouvelle-Écosse

Nunavut

Ontario

Saskatchewan

Terre-Neuve et Labrador

Territoires du Nord-Ouest

Québec

Yukon

J'habite à l'extérieur du Canada

Skip To: End of Survey If Dans quelle province candienne résidez-vous? = J'habite à l'extérieur du Canada

Role Quel est votre rôle principal vis-à-vis du TDAH?

Je suis chercheur/chercheuse dans le domaine du TDAH

Je suis clinicien(ne) au service d'une clientèle atteinte de TDAH

Je suis éducateur/éducatrice au service d'une clientèle atteinte de TDAH

Je suis étudiant(e)/stagiaire avec l'intention de poursuivre une carrière en recherche sur le TDAH

Je suis étudiant(e)/stagiaire et j'ai l'intention de continuer en tant que clinicien(ne)

Je suis étudiant(e)/stagiaire avec l'intention de continuer en tant qu'éducateur/trice

J'ai un TDAH

Je suis le parent/tuteur/tutrice, ou le/la conjoint(e) d'un proche vivant avec un TDAH

Autre (SVP préciser)

OtherRole Si 'autre', SVP précisez:

________________________________________________________________

| Page Break |  |
| --- | --- |

Display this question:

If Quel est votre rôle principal vis-à-vis du TDAH? = Je suis clinicien(ne) au service d'une clientèle atteinte de TDAH

Or Quel est votre rôle principal vis-à-vis du TDAH? = Je suis étudiant(e)/stagiaire avec l'intention de poursuivre une carrière en recherche sur le TDAH

Discipline Quelle est votre discipline principale?

- Psychiatrie adulte
- Psychologie adulte
- Pédopsychiatrie
- Pédopsychologie
- Psychothérapie
- Médicine familiale
- Pédiatrie
- Sciences infirmières
- Autre (SVP préciser)

Display this question:

If Quel est votre rôle principal vis-à-vis du TDAH? = Je suis clinicien(ne) au service d'une clientèle atteinte de TDAH

Or Quel est votre rôle principal vis-à-vis du TDAH? = Je suis étudiant(e)/stagiaire avec l'intention de poursuivre une carrière en recherche sur le TDAH

OtherDiscipline Autre:

________________________________________________________________

Attention Check1 Je vis sur la planète Terre.

Oui

Non

Age Quelle est votre catégorie d'âge?

16-24 ans

25-39 ans

30-59 ans

60+ years

Gender À quel genre vous identifiez-vous?

Homme

Femme

Autre

Je préfère ne pas le dire

End of Block: DEMOGRAPHICS

Start of Block: Assessment

Attention Check2 'B' est la première lettre de l'alphabet

Oui

Non

Start of Block: PRIORITIES

Instruction Nous aimerions connaître votre opinion sur les types de thèmes et enjeux liés au TDAH qui devraient être priorisés actuellement au Canada. Veuillez indiquer votre point de vue sur les sujets suivants.

SocialChallenges **Défis sociaux liés au TDAH** Lesquels de ces enjeux sociaux devraient être priorisés? Veuillez indiquer ceux que vous croyez sont importants, et non ceux qui pourraient bénéficier à la société de façon plus générale.

|  | Pas une priorité (1) | Priorité basse (2) | Indécis(e) (3) | Priorité élevée (4) | Priorité critique (5) |
| --- | --- | --- | --- | --- | --- |
| Améliorer l'accès aux fournisseurs de soins qui sont compétents pour évaluer le TDAH |  |  |  |  |  |
| Améliorer l'accès aux services pour le TDAH (p.ex. thérapie, coaching, formations axées sur les compétences, programmes d'emploi) |  |  |  |  |  |
| Améliorer l'accès au soutien aux proches (conjoints, parents, frères/soeurs) |  |  |  |  |  |
| Mieux financer la recherche sur le TDAH de manière générale |  |  |  |  |  |
| Offrir des programmes de logement pour les personnes avec TDAH |  |  |  |  |  |

SocialClarification Avez-vous des précisions ou détails supplémentaires à apporter vos évaluations précédentes?

________________________________________________________________

KTchallenges **Défis liés aux connaissances et à la sensibilisation au TDAH** Lesquels de ces enjeux liés aux connaissances et à la sensibilisation devraient être priorisés? Veuillez indiquer ceux que vous croyez sont importants, et non ceux qui pourraient bénéficier à la société de façon plus générale.

|  | Pas une priorité (1) | Priorité basse (2) | Indécis(e) (3) | Priorité élevée (4) | Priorité critique (5) |
| --- | --- | --- | --- | --- | --- |
| Améliorer les connaissances sur le TDAH chez les enseignants |  |  |  |  |  |
| Améliorer les connaissances sur le TDAH chez les parents |  |  |  |  |  |
| Améliorer la sensibilisation au TDAH dans les lieux de travail |  |  |  |  |  |
| Améliorer la sensibilisation au TDAH auprès du grand public (p.ex., via des campagnes nationales) |  |  |  |  |  |

KTClarification Avez-vous des précisions ou détails supplémentaires à apporter vos évaluations précédentes?

________________________________________________________________

Attention Check3 La Terre est plate.

Oui

Non

DXchallenges **Défis liés au diagnostic du TDAH** Lesquels de ces enjeux liés au diagnostic devraient être priorisés? Veuillez indiquer ceux que vous croyez sont importants, et non ceux qui pourraient bénéficier à la société de façon plus générale.

|  | Pas une priorité (1) | Priorité basse (2) | Indécis(e) (3) | Priorité élevée (4) | Priorité critique (5) |
| --- | --- | --- | --- | --- | --- |
| Études sur le diagnostic du TDAH chez les filles et les femmes |  |  |  |  |  |
| Études sur le diagnostic du TDAH chez les personnes plus âgées (50 ans et plus) |  |  |  |  |  |
| Études sur la façon dont d'autres difficultés (p.ex., dépression, anxiété) devraient être prises en compte lors du diagnostic d'un TDAH |  |  |  |  |  |
| Études sur les éléments à inclure dans un bilan diagnostique du TDAH (p.ex., évaluation cognitive) |  |  |  |  |  |
| Créer de nouveaux outils pour mesurer l'impact du TDAH sur le fonctionnement social et émotionnel |  |  |  |  |  |
| Études sur ce que réprésente une "difficulté de fonctionnement" liée aux symptômes du TDAH |  |  |  |  |  |

DxClarification Avez-vous des précisions ou détails supplémentaires à apporter vos évaluations précédentes?

________________________________________________________________

| Page Break |  |
| --- | --- |

TXchallenges **Défis liés au traitement du TDAH** Lesquels de ces enjeux liés au traitement devraient être priorisés? Veuillez indiquer ceux que vous croyez sont importants, et non ceux qui pourraient bénéficier à la société de façon plus générale.

|  | Pas une priorité (1) | Priorité basse (2) | Indécis(e) (3) | Priorité élevée (4) | Priorité critique (5) |
| --- | --- | --- | --- | --- | --- |
| Études sur de nouveaux traitements non-pharmacologiques |  |  |  |  |  |
| Études sur l'impact du TDAH sur les proches (parents, conjoints, frères/soeurs) |  |  |  |  |  |
| Études sur les conséquences à long terme du TDAH non traité |  |  |  |  |  |
| Études sur l'efficacité et la sécurité de l'utilisation des médicaments chez les personnes plus âgées (50 ans et plus) |  |  |  |  |  |
| Étudies sur comment optimiser l'adhésion au traitement (c.à.d. pour aider les gens à suivre leur régime de traitement) |  |  |  |  |  |
| Études sur les bénéfices de différents traitements, relatifs à leurs coûts («analyse coûts-bénéfices») |  |  |  |  |  |

TxClarification Avez-vous des précisions ou détails supplémentaires à apporter vos évaluations précédentes?

________________________________________________________________

| Page Break |  |
| --- | --- |

FreeTextSuggestion S'il y a lieu, nous vous invitons à apporter **d'autres** suggestions de priorités à considérer au niveau du TDAH:

________________________________________________________________

End of Block: PRIORITIES

Start of Block: ContactInfo

Giftcard Draw S'il vous plaît veuillez indiquer votre adresse courriel pour vous inscrire à un tirage d'une carte cadeau d'une valeur de 50$ (parmi dix).

Follow-up Nous aimerions vous recontacter dans quelques mois avec quelques autres questions très brèves afin de peaufiner notre liste de priorités liées au TDAH. Si vous participez, vous serez inscrit à un autre tirage de carte-cadeau. Si vous acceptez que nous vous recontactions, s'il vous plaît veuillez indiquer votre adresse courriel.

________________________________________________________________

End of Block: ContactInfo

Start of Block: EndBlock

ThankYou
 
**Merci pour vos réponses.**
 
Nous intégrerons vos réponses à celles des autres participants et vous recontacterons sous peu pour finaliser cette liste de priorités et affiner les points d'accord.
 

Si vous avez des commentaires supplémentaires, veuillez les saisir ici.

________________________________________________________________

End of Block: EndBlock

# Survey Round 2. English version

**Unravelling the Puzzles in ADHD: A Delphi Study (Wave 2)**

**Start of Block: CONSENT**

Consent Form
**Thank you for taking part in Wave 1 of our research study, "Unravelling the Puzzles in ADHD: Identifying Research Challenges and Opportunities across Canada Using a Delphi Approach."** In December 2022/January 2023, you completed a short survey asking you about your perspectives on topics and themes that should be prioritized in Canadian research on attention-deficit/hyperactivity disorder (ADHD). That survey was Wave 1 of a three-wave procedure (the goal of which is to achieve general consensus on important ADHD-related priorities by the third wave). Today you are being contacted to complete Wave 2, consisting of a similar brief survey. At most, **this survey should take you 10-15 minutes to complete**, and you will be entered into a draw to win one of ten $50 gift cards.   Please also expect to receive one final 5-minute survey in the coming months, which you will be asked to complete for a chance to be entered in another gift card draw. You have already consented to participating in this study; if you would like to review the consent form again, please click here. Thank you for your continued participation and very valuable input into this important project! Sincerely, The CAPS Team Canadian ***A****DHD* ***P****riorities* ***S****urvey*. The Ethics Board has approved this research study (REB22-0011). Participation is completely voluntary, and confidential. **Researcher**XX **Co-investigators** XX
 **Questions/Concerns** If you have any further questions or want clarification regarding this research and/or your participation, please contact: XX

**END of Block: CONSENT**

CliniciansPrompt Following the first survey conducted earlier this winter, we gathered the opinions of Canadian clinicians, including yours, on the types of ADHD-related topics and issues that should be a priority right now. Clinicians primarily identified themselves as having training in family medicine, psychiatry, psychology, social work, or occupational therapy. Please answer the next set of questions with your role, interests, and perspectives as an ADHD clinician in mind.

Q248 ***ISSUES RELATED TO HEALTHCARE QUALITY/ACCESS***

TrainedHCP_Clin Ninety percent (89.8%) of Canadian clinicians surveyed thought that **providing access to healthcare providers who are well-trained to recognize ADHD** is currently a *high priority* or *critical priority*. Considering this summary, please indicate the extent to which you feel **providing access to healthcare providers who are well-trained to recognize ADHD** is important to address.

- Critical priority
- High priority
- Undecided
- Low priority
- Not a priority

AccessServices_Clin Ninety-one percent (90.8%) of Canadian clinicians surveyed thought that **providing access to ADHD services (CBT, coaching, skills-based training, employment programs, etc.)** is currently a *high priority* or *critical priority*. Considering this summary, please

indicate the extent to which you feel **providing access to ADHD services (CBT, coaching, skills-based training, employment programs, etc.)** is important to address.

- Critical priority
- High priority
- Undecided
- Low priority
- Not a priority

FamilySupport_Clin Eighty-three percent (83.0%) of Canadian clinicians surveyed thought that **providing access to support for families (spouses, parents, siblings)** is currently a *high priority* or *critical priority*. Considering this summary, please indicate the extent to which you feel **providing access to support for families (spouses, parents, siblings)**  is important to address.

- Critical priority
- High priority
- Undecided
- Low priority
- Not a priority

NewAccess_Clin We also received the following **additional**suggestions related to finding/accessing care that may be important to consider in Canada. Please provide your

perspectives about these new topics, indicating what you think is important (not what you believe would necessarily benefit society at the general level).

|  | Not a priority (1) | Low priority (2) | Undecided (3) | High priority (4) | Critical priority (5) |
| --- | --- | --- | --- | --- | --- |
| Providing access to funded services for individuals with ADHD and their loved ones (for example, healthcare coverage for psychological services, and/or affordable options, etc.) |  |  |  |  |  |
| Providing more accessible information and support to navigate the healthcare system and find appropriate services/personnel to assist and advocate for individuals with ADHD |  |  |  |  |  |
| Providing individuals with ADHD with the tools, information, and strategies to self-advocate |  |  |  |  |  |
| Increasing the availability of social support networks for individuals, couples, and families with ADHD |  |  |  |  |  |
| Providing access to resources and services to smaller and/or rural communities |  |  |  |  |  |
| Increasing knowledge and training about ADHD and associated stigmas among all healthcare and mental health professionals (for example, family doctors, nurse practitioners, pharmacists, psychologists, counsellors, etc.) |  |  |  |  |  |
| Identifying delays/barriers to assessment and treatment, and the impacts they may have on different systems |  |  |  |  |  |
| Optimizing the assessment process through the use of validated tools to improve early diagnosis and diagnostic accuracy, and reduce misdiagnosis |  |  |  |  |  |

Q253 ***ISSUES RELATED TO ADHD AWARENESS IN SCHOOLS & WORKPLACES***

TeacherKnowledg_Clin Eighty-eight percent (87.9%) of Canadian clinicians surveyed thought that **increasing knowledge about ADHD among teachers and educators** is currently a *high priority* or *critical priority*. Considering this summary, please indicate the extent to which you feel **increasing knowledge about ADHD among teachers and educators** is important to address.

- Critical priority
- High priority
- Undecided
- Low priority
- Not a priority

WorkplaceKnowle_Clin Seventy-seven percent (77.2%) of Canadian clinicians surveyed thought that **increasing awareness about ADHD among employers and in workplaces** is currently a *high priority* or *critical priority*. Considering this summary, please indicate the extent to which you feel **increasing awareness about ADHD among employers and in workplaces** is important to address.

- Critical priority
- High priority
- Undecided
- Low priority
- Not a priority

NewSchoolWork_Clin We also received the following **additional** suggestions related to educational and workplace supports that may be important to consider in Canada. Please

provide your perspectives about these new topics, indicating what you think is important (not what you believe would necessarily benefit society at the general level).

|  | Not a priority (1) | Low priority (2) | Undecided (3) | High priority (4) | Critical priority (5) |
| --- | --- | --- | --- | --- | --- |
| Increasing the availability of adapted supports within the school system (for example, report cards specific to students with ADHD, ADHD-friendly learning and aftercare programs, etc.) |  |  |  |  |  |
| Educating personnel in the school systems on how to best support and teach individuals with ADHD |  |  |  |  |  |
| Providing basic general training in recognizing ADHD to all personnel who interact with youth in their line of work (for example, police, dentists, social workers, corrections officers, educators, etc.) |  |  |  |  |  |
| Increasing job opportunities and workplace accommodations for all employees with ADHD |  |  |  |  |  |

Q257 ***ISSUES RELATED TO ADHD AWARENESS IN COMMUNITIES***

ParentsKnowledg_Clin Eighty-five percent (85.4%) of Canadian clinicians surveyed thought that **increasing knowledge about ADHD among parents** is currently a *high priority* or *critical priority*. Considering this summary, please indicate the extent to which you feel **increasing knowledge about ADHD among parents** is important to address.

- Critical priority
- High priority
- Undecided
- Low priority
- Not a priority

PublicKnowledge_Clin Seventy-seven percent (77.2%) of Canadian clinicians surveyed thought that **increasing awareness about ADHD among the general public** is currently a *high priority*

or *critical priority*. Considering this summary, please indicate the extent to which you feel **increasing awareness about ADHD among the general public** is important to address.

- Critical priority
- High priority
- Undecided
- Low priority
- Not a priority

NewGenAware_Clin We also received the following **additional** suggestions related to general public awareness of ADHD that may be important to consider in Canada. Please provide your

perspectives about these new topics, indicating what you think is important (not what you believe would necessarily benefit society at the general level).

|  | Not a priority (1) | Low priority (2) | Undecided (3) | High priority (4) | Critical priority (5) |
| --- | --- | --- | --- | --- | --- |
| Redefining ADHD in a more positive/normative way as a facet of neurodiversity, to de-stigmatize and de-medicalize it (for example, by changing ADHD terminology to remove words like disorder, disability) |  |  |  |  |  |
| Increasing public awareness of the different ways ADHD can present (for example, on a spectrum, with different symptom types, can be ‘masked’, etc.) |  |  |  |  |  |
| Increasing awareness among the general public about treated vs. untreated ADHD and its implications among the general public (for example, through awareness campaigns, school presentations or healthcare presentations) |  |  |  |  |  |

Q261 ***ISSUES RELATED TO ADHD RESEARCH IN SPECIFIC GROUPS***

AxGirlsWomen_Clin Seventy-nine percent (79.1%) of Canadian clinicians surveyed thought that **research on diagnosing ADHD in girls and women** is currently a *high priority* or *critical priority*. Considering this summary, please indicate the extent to which you feel **research on diagnosing ADHD in girls and women** is important to address.

- Critical priority
- High priority
- Undecided
- Low priority
- Not a priority

AxOlderAdults_Clin Fifty-nine percent (58.7%) of Canadian clinicians surveyed thought that **research on diagnosing ADHD in older adults (age 50+)** is currently a *high priority* or *critical*

*priority*. Considering this summary, please indicate the extent to which you feel **research on diagnosing ADHD in older adults (age 50+)** is important to address.

- Critical priority
- High priority
- Undecided
- Low priority
- Not a priority

TxOlderAdults_Clin Sixty-two percent (61.7%) of Canadian clinicians surveyed thought that **research on how well treatment works, and how safe it is, for older adults (age 50+)** is currently a *high priority* or *critical priority*. Considering this summary, please indicate the extent to which you feel **research on how well treatment works, and how safe it is, for older adults (age 50+)** is important to address.

- Critical priority
- High priority
- Undecided
- Low priority
- Not a priority

AxComorbidities_Clin Eighty percent (80.1%) of Canadian clinicians surveyed thought that **research on how co-existing experiences (e.g., depression, anxiety) should be considered when diagnosing ADHD** is currently a *high priority* or *critical priority*. Considering this summary, please indicate the extent to which you feel **research on how co-existing experiences (e.g., depression, anxiety) should be considered when diagnosing ADHD** is important to address.

- Critical priority
- High priority
- Undecided
- Low priority
- Not a priority

NewSpecialGroups_Cli We also received the following **additional** suggestions related to expanding knowledge of ADHD to specific groups that may be important to consider in

Canada. Please provide your perspectives about these new topics, indicating what you think is important (not what you believe would necessarily benefit society at the general level).

|  | Not a priority (1) | Low priority (2) | Undecided (3) | High priority (4) | Critical priority (5) |
| --- | --- | --- | --- | --- | --- |
| Increasing general awareness of ADHD and its impacts in girls and women (for example, among healthcare providers, across the lifespan, education, workplace, etc.) |  |  |  |  |  |
| Research on the impact of hormones (for example, hormonal fluctuations, hormone replacement therapy or contraceptives, etc.) on ADHD symptoms, and their interactions with ADHD medications |  |  |  |  |  |
| Research on the impact of ADHD medications on hormonal fluctuations and reproductive health |  |  |  |  |  |
| Research on recognizing and diagnosing ADHD in mid-life (ages 35-50) |  |  |  |  |  |
| Expanding our understanding of ADHD in under-served or marginalized populations (for example, ethnic minorities, queer and gender-diverse communities, and Indigenous groups) |  |  |  |  |  |
| Research to understand the prevalence and unique experiences of people with ADHD and other mental health challenges |  |  |  |  |  |

Q267 ***ISSUES RELATED TO MEASURING THE SYMPTOMS & IMPACTS OF ADHD***

AxShouldInclude_Clin Seventy-five percent (75.2%) of Canadian clinicians surveyed thought that **research on what should be included when diagnosing ADHD (e.g., cognitive assessment)** is currently a *high priority* or *critical priority*. Considering this summary, please indicate the extent to which you feel **research on what should be included when diagnosing ADHD (e.g., cognitive assessment)** is important to address.

- Critical priority
- High priority
- Undecided
- Low priority
- Not a priority

AxEmotionDysreg_Clin Eighty percent (80.1%) of Canadian clinicians surveyed thought that **creating new tools to capture how ADHD impacts social relationships and emotion regulation** is currently a *high priority* or *critical priority*. Considering this summary, please

indicate the extent to which you feel **creating new tools to capture how ADHD impacts social relationships and emotion regulation** is important to address.

- Critical priority
- High priority
- Undecided
- Low priority
- Not a priority

AxImpairment_Clin Seventy percent (69.9%) of Canadian clinicians surveyed thought that **research on what it means to be "impaired" by symptoms of ADHD** is currently a *high priority* or *critical priority*. Considering this summary, please indicate the extent to which you feel **research on what it means to be "impaired" by symptoms of ADHD** is important to address.

- Critical priority
- High priority
- Undecided
- Low priority
- Not a priority

FamilyImpact_Clin Sixty-three percent (63.6%) of Canadian clinicians surveyed thought that **research on how ADHD impacts families (parents, partners, siblings)** is currently a *high priority* or *critical priority*. Considering this summary, please indicate the extent to which you

feel **research on how ADHD impacts families (parents, partners, siblings)** is important to address.

- Critical priority
- High priority
- Undecided
- Low priority
- Not a priority

NewAx_Clin We also received the following **additional** suggestions related to measuring the impacts of ADHD that may be important to consider in Canada. Please provide your

perspectives about these new topics, indicating what you think is important (not what you believe would necessarily benefit society at the general level).

|  | Not a priority (1) | Low priority (2) | Undecided (3) | High priority (4) | Critical priority (5) |
| --- | --- | --- | --- | --- | --- |
| Increasing knowledge and awareness about the impacts of inter-generational ADHD (many generations of ADHD within a family) |  |  |  |  |  |
| Research to expand our knowledge of ADHD and co-occurring health-related conditions (for example, related to sleep, eating, oral health, personal hygiene, etc.) |  |  |  |  |  |
| Research on socio-emotional functioning in ADHD (for example, self-esteem issues, ability to regulate emotions, etc.) and its impact on relationships |  |  |  |  |  |

Q273 ***ISSUES RELATED TO TREATMENTS FOR ADHD***

NondrugTx_Clin Seventy-one percent (70.9%) of Canadian clinicians surveyed thought that **research on new non-drug treatments** is currently a *high priority* or *critical priority*. Considering this summary, please indicate the extent to which you feel **research on new non-drug treatments** is important to address.

- Critical priority
- High priority
- Undecided
- Low priority
- Not a priority

TxAdherence_Clin Sixty percent (60.2%) of Canadian clinicians surveyed thought that **research on how to improve treatment compliance** is currently a *high priority* or *critical priority*.

Considering this summary, please indicate the extent to which you feel **research on how to improve treatment compliance** is important to address.

- Critical priority
- High priority
- Undecided
- Low priority
- Not a priority

LongTermCnsq_Clin Seventy-three percent (72.8%) of Canadian clinicians surveyed thought that **research on the long-term consequences of untreated ADHD** is currently a *high priority* or *critical priority*. Considering this summary, please indicate the extent to which you feel **research on the long-term consequences of untreated ADHD** is important to address.

- Critical priority
- High priority
- Undecided
- Low priority
- Not a priority

TxCostBenefit_Clin Sixty-four percent (64.1%) of Canadian clinicians surveyed thought that **research on the benefits of treatments, relative to their costs ('cost-benefit analysis')** is currently a *high priority* or *critical priority*. Considering this summary, please indicate the extent to which you feel **research on the benefits of treatments, relative to their costs ('cost-benefit analysis’) is** important to address.

- Critical priority
- High priority
- Undecided
- Low priority
- Not a priority

NewTx_Clin We also received the following **additional** suggestions related to ADHD treatments that may be important to consider in Canada. Please provide your perspectives about these

new topics, indicating what you think is important (not what you believe would necessarily benefit society at the general level).

|  | Not a priority (1) | Low priority (2) | Undecided (3) | High priority (4) | Critical priority (5) |
| --- | --- | --- | --- | --- | --- |
| Providing access to holistic treatment options supported by multi-disciplinary teams (for example, medication, nutrition, occupational therapy, etc.) embedded within different systems, including workplace and educational settings |  |  |  |  |  |
| Researching how different treatments affect different individuals in the short- and long-term, using a person-centered approach to tailor treatments to all ADHD individuals (including those with co-occurring challenges like mental health concerns, intellectual disabilities, autism, etc.) |  |  |  |  |  |
| Research on optimizing existing non-drug treatments (for example, meditation and mindfulness, psychotherapy, physical activity, acupuncture, etc.) |  |  |  |  |  |
| Educating individuals with ADHD and their loved ones about medication management, different medications available, and best treatment options |  |  |  |  |  |
| Research on best treatments for addictions within the context of ADHD, including addictions to substances, gaming, gambling, and screens |  |  |  |  |  |

| Page Break |  |
| --- | --- |

Q279 ***GENERAL RESEARCH DIRECTIONS***

Housing_Clin Thirty-four percent (34.0%) of Canadian clinicians surveyed thought that **providing housing programs for people with ADHD** is currently a *high priority* or *critical priority*. Considering this summary, please indicate the extent to which you feel **providing housing programs for people with ADHD** is important to address.

- Critical priority
- High priority
- Undecided
- Low priority
- Not a priority

GeneralFunding_Clin Seventy-three percent (72.8%) of Canadian clinicians surveyed thought that **providing general funding for ADHD research** is currently a *high priority* or *critical*

*priority*. Considering this summary, please indicate the extent to which you feel **providing general funding for ADHD research** is important to address.

- Critical priority
- High priority
- Undecided
- Low priority
- Not a priority

NewGeneral_Clin We also received the following **additional** suggestions related to general ADHD research directions that may be important to consider in Canada. Please provide your

perspectives about these new topics, indicating what you think is important (not what you believe would necessarily benefit society at the general level).

|  | Not a priority (1) | Low priority (2) | Undecided (3) | High priority (4) | Critical priority (5) |
| --- | --- | --- | --- | --- | --- |
| Continuing to work towards a better understanding of the causes of ADHD (for example, genetics, heritability, neurological mechanisms, risk factors, etc.) |  |  |  |  |  |
| Encouraging positive-directed research to better understand the unique strengths of those with ADHD |  |  |  |  |  |
| Including people with lived experience in the process of research about ADHD |  |  |  |  |  |
| Targeted research examining the stigmatization of ADHD (for example, feelings of self-stigma, parental stigma regarding their children, stigmatization in schools or classrooms) |  |  |  |  |  |
| Increasing understanding of ADHD as a condition warranting recognition by government and educational systems |  |  |  |  |  |

Q534 You have reached the end of the **clinician**survey.

**End of Block: CLINICIANS_PRIORITIES**

**Start of Block: CONTACT**

Giftcard Draw Thank you for your responses to Wave 2 of this study! Please enter your email address below, if you wish to be entered in a draw to win one of ten $50 gift cards.

________________________________________________________________

Follow-up We would like to contact you once more in a few weeks from now, to ask you a few more very brief questions to refine our list of ADHD-related priorities. **This final survey should take, at most, 5 minutes of your time.**If you participate, you will be entered in another gift card draw. If you accept to be contacted, please enter your email address.

________________________________________________________________

**End of Block: CONTACT**

**Start of Block: EndBlock**

**Thank you for your responses.**

We will integrate your responses with those of other participants, and re-contact you again shortly to finalize this priority list and hone in on areas of agreement.

If you have any additional feedback, please enter it here.

________________________________________________________________

**End of Block: EndBlock**

# Survey Round 2. French version

**Unravelling the Puzzles in ADHD: A Delphi Study (Wave 2)**

**Start of Block: CONSENT**

Consent Form
**Merci d'avoir participé à la première partie de notre étude de recherche, "Démêler les énigmes du TDAH avec la méthode Delphi : défis et opportunités de recherche à travers le Canada."** En décembre 2022/janvier 2023, vous avez répondu à un court sondage qui sollicitait votre perspective sur des sujets et des thèmes qui devraient être priorisés dans la recherche canadienne sur le trouble déficitaire de l'attention/hyperactivité (TDAH). Cette enquête était la première partie d'une procédure en trois parties, dont le but ultime est d'atteindre un consensus général sur les priorités importantes liées au TDAH. Aujourd'hui, nous vous contactons pour répondre à la deuxième partie de l'étude, consistant en un bref sondage similaire au premier. Tout au plus, ce sondage devrait vous prendre 10 à 15 minutes à compléter, et vous aurez la chance de gagner l'une de dix cartes-cadeaux de 50 $ dans un tirage.   Veuillez également vous attendre à recevoir un dernier sondage de 5 minutes au cours des prochains mois, suite auquel vous aurez la chance de participer à un autre tirage de cartes-cadeaux. Vous avez déjà consenti à participer à cette étude; si vous souhaitez revoir le formulaire de consentement, veuillez cliquer ici.   Merci pour votre contribution très précieuse à ce projet important! Sincèrement, L'équipe d'étude CAPS ***C****anadian* ***A****DHD* ***P****riorities* ***S****urvey* Cette étude a été approuvée par le comité d'éthique de la recherche (REB22-0011). Votre participation est entièrement volontaire et confidentielle. **Chercheure:** XX  **Co-chercheures:** XX. **Questions** Si vous avez des questions ou voulez obtenir des informations supplémentaires concernant l'étude et/ou votre participation, veuillez contacter XX.

**End of Block: CONSENT**

**Start of Block: CLINICIANS_PRIORITIES**

CliniciansPrompt Suite au premier sondage réalisé plus tôt cet hiver, nous avons colligé les opinions des **cliniciens**  canadiens, incluant les vôtres, sur les types de thèmes et de problèmes liés au TDAH qui devraient être prioritaires actuellement. Les cliniciens se sont identifiés principalement comme ayant une formation en médecine familiale, en psychiatrie, en psychologie, en travail social ou en ergothérapie. Veuillez répondre à la prochaine série de questions en pensant à votre rôle, vos intérêts et vos perspectives en tant que **clinicien(ne)** dans le domaine du TDAH.

Q248 ***ENJEUX LIÉS À LA QUALITÉ ET À L'ACCÈS AUX SOINS***

TrainedHCP_Clin Quatre-vingt-dix pourcent (89,8 %) des cliniciens canadiens sondés pensaient que **d’améliorer l'accès aux fournisseurs de soins qui sont compétents pour évaluer le TDAH** est actuellement une *priorité élevée* ou *critique*. Considérant ce résumé, veuillez indiquer dans quelle mesure vous pensez qu'il est important **d'améliorer** **l'accès aux fournisseurs de soins qui sont compétents pour évaluer le TDAH.**

- Priorité critique
- Priorité élevée
- Indécis(e)
- Priorité basse
- Pas une priorité

AccessServices_Clin Quatre-vingt-onze pourcent (90,8 %) des cliniciens canadiens sondés pensaient que **d'améliorer l'accès aux services pour le TDAH (p.ex. thérapie, coaching, formations axées sur les compétences, programmes d'emploi)** est actuellement une *priorité élevée* ou *critique.* Considérant ce résumé, veuillez indiquer dans quelle mesure vous pensez qu'il est important **d'améliorer l'accès aux services pour le TDAH (p.ex. thérapie, coaching, formations axées sur les compétences, programmes d'emploi).**

- Priorité critique
- Priorité élevée
- Indécis(e)
- Priorité basse
- Pas une priorité

FamilySupport_Clin Quatre-vingt-trois pourcent (83,0 %) des cliniciens canadiens sondés pensaient que **d'améliorer l'accès au soutien aux proches (conjoints, parents, frères/sœurs)** est actuellement une *priorité élevée* ou *critique.* Considérant ce résumé, veuillez indiquer dans quelle mesure vous pensez qu'il est important **d'améliorer l'accès au soutien aux proches (conjoints, parents, frères/sœurs).**

- Priorité critique
- Priorité élevée
- Indécis(e)
- Priorité basse
- Pas une priorité

NewAccess_Clin Nous avons également reçu des **suggestions supplémentaires** concernant la qualité et l'accès aux soins qu'il pourrait être important d'envisager au Canada. Veuillez donner votre point de vue sur ces nouveaux sujets, en indiquant ce que vous pensez être important (et non ce qui, selon vous, serait nécessairement bénéfique pour la société au niveau général).

|  | Pas une priorité (1) | Priorité basse (2) | Indécis(e) (3) | Priorité élevée (4) | Priorité critique (5) |
| --- | --- | --- | --- | --- | --- |
| Fournir un accès à des services financés aux personnes avec un TDAH et à leurs proches (par exemple, une couverture médicale pour les services psychologiques et/ou des options abordables, etc.) |  |  |  |  |  |
| Fournir des informations et un soutien plus accessibles pour naviguer dans le système de santé et trouver les services et le personnel appropriés au TDAH |  |  |  |  |  |
| Fournir aux personnes avec un TDAH les outils, les informations et les stratégies nécessaires pour renforcer leur autonomie sociale |  |  |  |  |  |
| Accroître la disponibilité des réseaux de soutien social pour les individus, les couples et les familles avec un TDAH |  |  |  |  |  |
| Fournir un accès aux ressources et aux services dans les communautés rurales |  |  |  |  |  |
| Accroître les connaissances et la formation sur le TDAH et la stigmatisation associée chez tous les professionnels de la santé et de la santé mentale (par exemple, les médecins de famille, les infirmières, les pharmaciens, les psychologues, etc.) |  |  |  |  |  |
| Identifier les obstacles à l'évaluation et au traitement, et les impacts qu'ils peuvent avoir sur différents systèmes |  |  |  |  |  |
| Optimiser le processus d'évaluation grâce à l'utilisation d'outils validés pour améliorer le diagnostic précoce et la précision du diagnostic, et réduire les erreurs diagnostiques |  |  |  |  |  |

Q253 ***ENJEUX LIÉS À LA SENSIBILISATION AU TDAH DANS LES ÉCOLES ET LES LIEUX DE TRAVAIL***

TeacherKnowledg_Clin Quatre-vingt-huit pourcent (88,0 %) des cliniciens canadiens sondés pensaient que **d'améliorer les connaissances sur le TDAH chez les enseignants** est actuellement une *priorité élevée* ou *critique.* Considérant ce résumé, veuillez indiquer dans quelle mesure vous pensez qu'il est important **d'améliorer les connaissances sur le TDAH chez les enseignants.**

- Priorité critique
- Priorité élevée
- Indécis(e)
- Priorité basse
- Pas une priorité

WorkplaceKnowle_Clin Soixante-dix-sept pourcent (77,2 %) des cliniciens canadiens sondés pensaient que **d'améliorer la sensibilisation au TDAH dans les lieux de travail** est actuellement une *priorité élevée* ou *critique.* Considérant ce résumé, veuillez indiquer dans quelle mesure vous pensez qu'il est important **d'améliorer la sensibilisation au TDAH dans les lieux de travail.**

- Priorité critique
- Priorité élevée
- Indécis(e)
- Priorité basse
- Pas une priorité

NewSchoolWork_Clin Nous avons également reçu des **suggestions supplémentaires** concernant le TDAH dans les écoles et lieux de travail qu'il pourrait être important d'envisager au Canada. Veuillez donner votre point de vue sur ces nouveaux sujets, en indiquant ce que vous pensez être important (et non ce qui, selon vous, serait nécessairement bénéfique pour la société au niveau général).

|  | Pas une priorité (1) | Priorité basse (2) | Indécis(e) (3) | Priorité élevée (4) | Priorité critique (5) |
| --- | --- | --- | --- | --- | --- |
| Accroître la disponibilité de soutiens adaptés au sein du système scolaire (par exemple, bulletins scolaires spécifiques aux élèves avec un TDAH, programmes d'apprentissage et de suivi adaptés au TDAH, etc.) |  |  |  |  |  |
| Sensibiliser le personnel scolaire sur les meilleures façons de soutenir et d'enseigner aux personnes avec un TDAH |  |  |  |  |  |
| Fournir une formation générale de base sur la reconnaissance du TDAH à tout le personnel qui interagit avec les jeunes dans leur domaine de travail (par exemple, la police, les dentistes, les travailleurs sociaux, les agents correctionnels, les éducateurs, etc.) |  |  |  |  |  |
| Accroître les possibilités d'emploi et les aménagements en milieu de travail pour tous les employés avec un TDAH |  |  |  |  |  |

Q257 ***ENJEUX LIÉS À LA SENSIBILISATION AU TDAH DANS LES COMMUNAUTÉS***

ParentsKnowledg_Clin Quatre-vingt-cinq pourcent (85,4 %) des cliniciens canadiens sondés pensaient que **d'améliorer les connaissances sur le TDAH chez les parents** est actuellement une *priorité élevée* ou *critique.* Considérant ce résumé, veuillez indiquer dans quelle mesure vous pensez qu'il est important **d'améliorer les connaissances sur le TDAH chez les parents.**

- Priorité critique
- Priorité élevée
- Indécis(e)
- Priorité basse
- Pas une priorité

PublicKnowledge_Clin Soixante-dix-sept pourcent (77,2 %) des cliniciens canadiens sondés pensaient que **d'améliorer la sensibilisation au TDAH auprès du grand public (p.ex., via des campagnes nationales)**est actuellement une *priorité élevée* ou *critique*. Considérant ce résumé, veuillez indiquer dans quelle mesure vous pensez qu'il est important **d'améliorer la sensibilisation au TDAH auprès du grand public (p.ex., via des campagnes nationales).**

- Priorité critique
- Priorité élevée
- Indécis(e)
- Priorité basse
- Pas une priorité

NewGenAware_Clin Nous avons également reçu des **suggestions supplémentaires** liées à la sensibilisation au TDAH qu'il pourrait être important d'envisager au Canada. Veuillez donner votre point de vue sur ces nouveaux sujets, en indiquant ce que vous pensez être important (et non ce qui, selon vous, serait nécessairement bénéfique pour la société au niveau général).

|  | Pas une priorité (1) | Priorité basse (2) | Indécis(e) (3) | Priorité élevée (4) | Priorité critique (5) |
| --- | --- | --- | --- | --- | --- |
| Redéfinir le TDAH de manière plus positive/normative comme une facette de la neurodiversité, pour le déstigmatiser et le démédicaliser (par exemple, en remplaçant les mots comme trouble, handicap) |  |  |  |  |  |
| Accroître la sensibilisation du public aux différentes façons dont le TDAH peut se présenter (par exemple, sur un spectre, avec différents types de symptômes, peut être "masqué", etc.) |  |  |  |  |  |
| Accroître la sensibilisation du grand public sur le TDAH traité par rapport au TDAH non traité et ses implications (par exemple, par le biais de campagnes de sensibilisation, de présentations dans les écoles ou dans les milieux de santé) |  |  |  |  |  |

Q261 ***ENJEUX LIÉS À LA RECHERCHE SUR LE TDAH DANS DES GROUPES SPÉCIFIQUES***

AxGirlsWomen_Clin Soixante-dix-neuf pourcent (79,1 %) des cliniciens canadiens sondés pensaient que la **recherche sur le diagnostic du TDAH chez les filles et les femmes** est actuellement une *priorité élevée* ou *critique.* Considérant ce résumé, veuillez indiquer dans quelle mesure vous pensez qu'il est important d'aborder la **recherche sur le diagnostic du TDAH chez les filles et les femmes.**

- Priorité critique
- Priorité élevée
- Indécis(e)
- Priorité basse
- Pas une priorité

AxOlderAdults_Clin Cinquante-neuf pourcent (58,7 %) des cliniciens canadiens sondés pensaient que la **recherche sur le diagnostic du TDAH chez les personnes plus âgées (50 ans et plus)** est actuellement une *priorité élevée* ou *critique.* Considérant ce résumé, veuillez indiquer dans quelle mesure vous pensez qu'il est important de rechercher **le diagnostic du TDAH chez les personnes plus âgées (50 ans et plus).**

- Priorité critique
- Priorité élevée
- Indécis(e)
- Priorité basse
- Pas une priorité

TxOlderAdults_Clin Soixante-deux pourcent (61,7 %) des cliniciens canadiens sondés pensaient que la **recherche sur l'efficacité et la sécurité de l'utilisation des médicaments chez les personnes plus âgées (50 ans et plus)** est actuellement une *priorité élevée* ou *critique.*  Considérant ce résumé, veuillez indiquer dans quelle mesure vous pensez qu'il est important de rechercher **l'efficacité et la sécurité de l'utilisation des médicaments chez les personnes plus âgées (50 ans et plus).**

- Priorité critique
- Priorité élevée
- Indécis(e)
- Priorité basse
- Pas une priorité

AxComorbidities_Clin Quatre-vingt pourcent (80,1 %) des cliniciens canadiens sondés pensaient que la **recherche sur la façon dont d'autres difficultés (p.ex., dépression, anxiété) devraient être prises en compte lors du diagnostic d'un TDAH** est actuellement une *priorité élevée* ou *critique*. Considérant ce résumé, veuillez indiquer dans quelle mesure vous pensez qu'il est important de rechercher **la façon dont d'autres difficultés (p.ex., dépression, anxiété) devraient être prises en compte lors du diagnostic d'un TDAH.**

- Priorité critique
- Priorité élevée
- Indécis(e)
- Priorité basse
- Pas une priorité

NewSpecialGroups_Cli Nous avons également reçu des **suggestions supplémentaires** concernant l'élargissement des connaissances sur le TDAH à des groupes spécifiques qu'il pourrait être important d'envisager au Canada. Veuillez donner votre point de vue sur ces nouveaux sujets, en indiquant ce que vous pensez être important (et non ce qui, selon vous, serait nécessairement bénéfique pour la société au niveau général).

|  | Pas une priorité (1) | Priorité basse (2) | Indécis(e) (3) | Priorité élevée (4) | Priorité critique (5) |
| --- | --- | --- | --- | --- | --- |
| Accroître la sensibilisation générale au TDAH et à ses impacts chez les filles et les femmes (par exemple, auprès des professionnels de la santé, dans les écoles et les lieux de travail, en adoptant une perspective de parcours de vie, etc.) |  |  |  |  |  |
| Études sur l'impact des hormones (par exemple, les fluctuations hormonales, l'hormonothérapie substitutive ou les contraceptifs, etc.) sur les symptômes du TDAH, et leurs interactions avec les médicaments pour le TDAH |  |  |  |  |  |
| Études sur l'impact des médicaments pour le TDAH sur les fluctuations hormonales et la santé reproductive |  |  |  |  |  |
| Études sur la reconnaissance et le diagnostic du TDAH dans la quarantaine (35-50 ans) |  |  |  |  |  |
| Répandre notre compréhension du TDAH dans les populations mal desservies ou marginalisées (par exemple, les minorités ethniques, les communautés LGBTQ+, et les groupes autochtones) |  |  |  |  |  |
| Études pour comprendre la prévalence et les expériences uniques des personnes vivant avec des troubles de santé mentale en plus du TDAH |  |  |  |  |  |

Q267 ***ENJEUX LIÉS AUX SYMPTÔMES ET AUX IMPACTS DU TDAH***

AxShouldInclude_Clin Soixante-quinze pourcent (75,2 %) des cliniciens canadiens sondés pensaient que la **recherche sur les éléments à inclure dans un bilan diagnostique du TDAH (p.ex., évaluation cognitive)** est actuellement une *priorité élevée* ou *critique.* Considérant ce résumé, veuillez indiquer dans quelle mesure vous pensez qu'il est important de **rechercher les éléments à inclure dans un bilan diagnostique du TDAH (p.ex., évaluation cognitive).**

- Priorité critique
- Priorité élevée
- Indécis(e)
- Priorité basse
- Pas une priorité

AxEmotionDysreg_Clin Quatre-vingt pourcent (80,1 %) des cliniciens canadiens sondés pensaient que la **création de nouveaux outils pour mesurer l'impact du TDAH sur le fonctionnement social et émotionnel** est actuellement une *priorité élevée* ou *critique.*  Considérant ce résumé, veuillez indiquer dans quelle mesure vous pensez qu'il est important de créer **de nouveaux outils pour mesurer l'impact du TDAH sur le fonctionnement social et émotionnel.**

- Priorité critique
- Priorité élevée
- Indécis(e)
- Priorité basse
- Pas une priorité

AxImpairment_Clin Soixante-dix pourcent (69,9 %) des cliniciens canadiens sondés pensaient que la **recherche sur ce que représente une "difficulté de fonctionnement" liée aux symptômes du TDAH** est actuellement une *priorité élevée* ou *critique*. Considérant ce résumé, veuillez indiquer dans quelle mesure vous pensez qu'il est important de **rechercher ce que représente une "difficulté de fonctionnement" liée aux symptômes du TDAH.**

- Priorité critique
- Priorité élevée
- Indécis(e)
- Priorité basse
- Pas une priorité

FamilyImpact_Clin Soixante-quatre pourcent (63,6 %) des cliniciens canadiens sondés pensaient que la **recherche sur l'impact du TDAH sur les proches (parents, conjoints, frères/sœurs)** est actuellement une *priorité élevée* ou *critique*. Considérant ce résumé, veuillez indiquer dans quelle mesure vous pensez qu'il est important d'aborder la **recherche sur l'impact du TDAH sur les proches (parents, conjoints, frères/sœurs).**

- Priorité critique
- Priorité élevée
- Indécis(e)
- Priorité basse
- Pas une priorité

NewAx_Clin Nous avons également reçu des **suggestions supplémentaires** suivantes liées aux impacts du TDAH qu'il pourrait être important d'envisager au Canada. Veuillez donner votre point de vue sur ces nouveaux sujets, en indiquant ce que vous pensez être important (et non ce qui, selon vous, serait nécessairement bénéfique pour la société au niveau général).

|  | Pas une priorité (1) | Priorité basse (2) | Indécis(e) (3) | Priorité élevée (4) | Priorité critique (5) |
| --- | --- | --- | --- | --- | --- |
| Accroître les connaissances et la sensibilisation aux impacts du TDAH intergénérationnel (plusieurs générations de TDAH au sein d'une famille) |  |  |  |  |  |
| Études pour élargir nos connaissances sur le TDAH et les problématiques concomitantes liées à la santé (par exemple, liées au sommeil, à l'alimentation, à la santé bucco-dentaire, à l'hygiène personnelle, etc.) |  |  |  |  |  |
| Études sur le fonctionnement socio-émotionnel dans le TDAH (par exemple, les problèmes d'estime de soi, la capacité à réguler ses émotions, etc.) et son impact sur les relations interpersonnelles |  |  |  |  |  |

Q273 ***ENJEUX LIÉS AUX TRAITEMENTS POUR LE TDAH***

NondrugTx_Clin Soixante-et-onze pourcent (70,9 %) des cliniciens canadiens sondés pensaient que la **recherche sur les nouveaux traitements non-pharmacologiques** est actuellement une *priorité élevée* ou *critique*. Considérant ce résumé, veuillez indiquer dans quelle mesure vous pensez qu'il est important d'aborder la **recherche sur les nouveaux traitements non-pharmacologiques.**

- Priorité critique
- Priorité élevée
- Indécis(e)
- Priorité basse
- Pas une priorité

TxAdherence_Clin Soixante pourcent (60,2 %) des cliniciens canadiens sondés pensaient que la **recherche sur comment optimiser l'adhésion au traitement (c.à.d. pour aider les gens à suivre leur régime de traitement)** est actuellement une *priorité élevée* ou *critique.*  Considérant ce résumé, veuillez indiquer dans quelle mesure vous estimez qu'il est important d'aborder la **recherche comment optimiser l'adhésion au traitement (c.à.d. pour aider les gens à suivre leur régime de traitement).**

- Priorité critique
- Priorité élevée
- Indécis(e)
- Priorité basse
- Pas une priorité

LongTermCnsq_Clin Soixante-treize pourcent (72,8 %) des cliniciens canadiens sondés pensaient que la **recherche sur les conséquences à long terme du TDAH non traité** est actuellement une *priorité élevée* ou *critique.* Considérant ce résumé, veuillez indiquer dans quelle mesure vous pensez qu'il est important d'aborder la **recherche sur les conséquences à long terme du TDAH non traité.**

- Priorité critique
- Priorité élevée
- Indécis(e)
- Priorité basse
- Pas une priorité

TxCostBenefit_Clin Soixante-quatre pourcent (64,1 %) des cliniciens canadiens sondés pensaient que la **recherche sur les bénéfices de différents traitements, relatifs à leurs coûts («analyse coûts-bénéfices»)** est actuellement une *priorité élevée* ou *critique*. Considérant ce résumé, veuillez indiquer dans quelle mesure vous estimez qu'il est important d'aborder la **recherche sur les bénéfices de différents traitements, relatifs à leurs coûts («analyse coûts-bénéfices»).**

- Priorité critique
- Priorité élevée
- Indécis(e)
- Priorité basse
- Pas une priorité

NewTx_Clin Nous avons également reçu des **suggestions supplémentaires** suivantes concernant le traitement du TDAH qu'il pourrait être important d'envisager au Canada. Veuillez donner votre point de vue sur ces nouveaux sujets, en indiquant ce que vous pensez être important (et non ce qui, selon vous, serait nécessairement bénéfique pour la société au niveau général).

|  | Pas une priorité (1) | Priorité basse (2) | Indécis(e) (3) | Priorité élevée (4) | Priorité critique (5) |
| --- | --- | --- | --- | --- | --- |
| Fournir un accès à des options de traitement holistiques soutenues par des équipes multidisciplinaires (par exemple, médicaments, nutrition, ergothérapie, etc.) intégrées dans différents systèmes, y compris les milieux de travail et d'enseignement |  |  |  |  |  |
| Études sur comment différents traitements affectent différentes personnes à court et à long terme, en utilisant une approche centrée sur la personne pour adapter les traitements à toutes les personnes avec un TDAH (y compris celles présentant des problèmes concomitants tels que des problèmes de santé mentale, l'autisme, etc.) |  |  |  |  |  |
| Études sur l'optimisation des traitements non-pharmacologiques existants (par exemple, la méditation et la pleine conscience, la psychothérapie, l'activité physique, l'acuponcture, etc.) |  |  |  |  |  |
| Éduquer les personnes avec un TDAH et leurs proches sur la gestion des médicaments, les différents médicaments disponibles, et les meilleures options de traitement |  |  |  |  |  |
| Études sur les meilleurs traitements pour les dépendances dans le contexte du TDAH, y compris les dépendances aux substances, au jeu, et aux écrans |  |  |  |  |  |

Q279 ***ORIENTATIONS GÉNÉRALES DE RECHERCHE***

Housing_Clin Trente-quatre pourcent (34,0 %) des cliniciens canadiens sondés pensaient que **d'offrir des programmes de logement aux personnes avec un TDAH** est actuellement une *priorité élevée* ou *critique*. Considérant ce résumé, veuillez indiquer dans quelle mesure vous pensez qu'il est important **d'offrir des programmes de logement aux personnes avec un TDAH.**

- Priorité critique
- Priorité élevée
- Indécis(e)
- Priorité basse
- Pas une priorité

GeneralFunding_Clin Soixante-treize pourcent (72,8 %) des cliniciens canadiens sondés pensaient que **de mieux financer la recherche sur le TDAH de manière générale** est actuellement une *priorité élevée* ou *critique*. Considérant ce résumé, veuillez indiquer dans quelle mesure vous pensez qu'il est important de **mieux financer la recherche sur le TDAH de manière générale.**

- Priorité critique
- Priorité élevée
- Indécis(e)
- Priorité basse
- Pas une priorité

NewGeneral_Clin Nous avons également reçu des **suggestions supplémentaires** concernant les orientations générales de recherche sur le TDAH qu'il pourrait être important d'envisager au Canada. Veuillez donner votre point de vue sur ces nouveaux sujets, en indiquant ce que vous pensez être important (et non ce qui, selon vous, serait nécessairement bénéfique pour la société au niveau général).

|  | Pas une priorité (1) | Priorité basse (2) | Indécis(e) (3) | Priorité élevée (4) | Priorité critique (5) |
| --- | --- | --- | --- | --- | --- |
| Continuer à travailler vers une meilleure compréhension des causes du TDAH (par exemple, la génétique, l'héritabilité, les mécanismes neurologiques, les facteurs de risque, etc.) |  |  |  |  |  |
| Encourager la recherche orientée positivement pour mieux comprendre les forces uniques des personnes avec un TDAH |  |  |  |  |  |
| Inclure des personnes ayant un vécu du TDAH dans le processus de recherche sur le TDAH |  |  |  |  |  |
| Études ciblées examinant la stigmatisation du TDAH (par exemple, l'auto-stigmatisation, la stigmatisation des parents à l'égard de leurs enfants, la stigmatisation dans les écoles) |  |  |  |  |  |
| Accroître la compréhension du TDAH en tant que condition qui doit être reconnue par le gouvernement et les systèmes éducatifs |  |  |  |  |  |

Q534 Vous avez terminé le sondage à l'intention des **cliniciens**.

**End of Block: CLINICIANS_PRIORITIES**

**Start of Block: CONTACT**

Giftcard Draw S'il vous plaît veuillez indiquer votre adresse courriel pour participer au tirage d'une carte-cadeau d'une valeur de 50$ (parmi dix).

________________________________________________________________

Follow-up Nous aimerions vous recontacter dans quelques semaines avec quelques autres questions très brèves afin de peaufiner notre liste de priorités liées au TDAH. **Cette enquête finale devrait prendre au maximum 5 minutes de votre temps.**Si vous participez, vous serez inscrit(e) à un autre tirage de carte-cadeau. Si vous acceptez que nous vous recontactions, s'il vous plaît veuillez indiquer votre adresse courriel.

________________________________________________________________

**End of Block: CONTACT**

**Start of Block: EndBlock**

ThankYou
**Merci pour vos réponses.**  
Nous intégrerons vos réponses à celles des autres participants et vous recontacterons sous peu pour finaliser cette liste de priorités et affiner les points d'accord. Si vous avez des commentaires supplémentaires, veuillez les saisir ici.

________________________________________________________________

**End of Block: EndBlock**

# Survey Round 3. English version

FINAL WAVE 3 - Unravelling the Puzzles in ADHD: A Delphi Study

Start of Block: CONSENT

Consent Form
**Thank you for participating in our research study, "Unravelling the Puzzles in ADHD: Identifying Research Challenges and Opportunities across Canada Using a Delphi Approach."** Earlier this year, you completed two surveys asking you about your perspectives on topics that should be prioritized in Canadian research on attention-deficit/hyperactivity disorder (ADHD), with the ultimate goal of achieving general consensus on important ADHD-related priorities. Today you are being contacted to participate in the final component of this study, consisting of a **10-15 minute survey.** Upon completion, you will be entered into a draw to win one of ten $50 gift cards. You have already consented to participating in this study; if you would like to review the consent form again, please click here. Thank you for your continued participation and very valuable input into this important project! Sincerely, The CAPS Team ***C****anadian* ***A****DHD* ***P****riorities* ***S****urvey*. The Research Ethics Board has approved this research study (REB22-0011). Participation is completely voluntary, and confidential. **Researcher** XX. **Co-investigators** XX.
 **Questions/Concerns** If you have any further questions or want clarification regarding this research and/or your participation, please contact XX.

End of Block: CONSENT

Start of Block: CLINICIANS_PRIORITIES

Text_DemoIntro **Next, we will be asking you some questions about your primary role with regard to ADHD and your personal background.**

YearsExp_ClinR1 How many years of experience do you have in clinical practice?

- < 5 years
- 5-10 years
- 11-20 years
- > 20 years

Specialty_ClinR1 Where did you complete your clinical specialty?

- I graduated from my specialty **in Canada**
- I graduated from my specialty **outside Canada**

PractSetting_ClinR1 Which setting do you currently practice in?

- I practice in an **urban** setting
- I practice in a **rural** setting

AttentionCheck Please select "strongly agree" for this question.

- Strongly agree
- Agree
- Neither agree nor disagree
- Disagree
- Strongly disagree

Service_ClinR1 What type of services do you provide?

- Insured only
- Non-insured/private only
- Both insured and non-insured/private

Assess_ClinR1 What type of assessments do you provide?

- In-person only
- Virtual only
- Both in-person and virtual

Background_ClinR1 Please specify the racial or ethnic background of your family. (Select all that apply)

- Caucasian/European/White
- Black/African
- Latin American/Hispanic (e.g. Mexican, Chilean, etc.)
- Middle Eastern/Arab/West Asian (e.g. Egyptian, Kuwaiti, Iranian, etc.)
- South Asian/East Indian (e.g. Pakistani, Sri Lankan, etc.)
- Asian (e.g. Chinese, Korean, Japanese, etc.)
- Filipino/Pacific Islander
- First Nations/Aboriginal
- Metis
- Inuit/Inuk
- Prefer not to say
- Other (Please specify): __________________________________________________

EducLevel_ClinR1 What is your highest level of completed education?

- Less than high school education
- Some high school, no diploma
- High school graduate, diploma or an equivalent
- Some college credit, no degree
- Trade/technical/vocational training
- Professional Certificate/Diploma
- Bachelor’s degree
- Master’s degree
- Doctorate degree

AnnualIncome_ClinR1 What is your family's annual income range?

- Less than $25,000
- Between $25,001 and $50,000
- Between $50,001 and $75,000
- Between $75,001 and $100,000
- Between $100,001 and $150,000
- Between $150,001 and $200,000
- Greater than $200,001

Age_ClinR1 What is your current age?

________________________________________________________________

JobStatus_ClinR1 What is your current employment status?

- Employed full-time
- Employed part-time
- Self-employed
- Unemployed
- Student
- Retired
- Other: __________________________________________________

Gender_ClinR1 What gender do you identify as?

- Man
- Woman
- Other
- Prefer not to say

ProvReside_ClinR1 What Canadian province or territory do you reside in?

- Alberta
- British Columbia
- Manitoba
- New Brunswick
- Newfoundland and Labrador
- Northwest Territories
- Nova Scotia
- Nunavut
- Ontario
- Prince Edward Island
- Québec
- Saskatchewan
- Yukon
- I do not live in Canada

ClinicianPrompt From the previous surveys completed earlier this year, we have reviewed the opinions of Canadian **clinicians**(including yourself) about the types of ADHD-related themes and issues that should be prioritized presently. Please complete the next series of questions thinking about your role, interests, and perspectives as an ADHD **clinician**.

Title_AccessToCare ***ISSUES RELATED TO HEALTHCARE QUALITY/ACCESS***

FundedService_ClinR1 Ninety-six percent (96.3%) of Canadian clinicians surveyed thought that **providing access to funded services for individuals with ADHD and their loved ones (for example, healthcare coverage for psychological services, and/or affordable options, etc.)** is currently a *high priority* or *critical priority*. Considering this summary, please indicate the extent to which you feel **providing access to funded services for individuals with ADHD and their loved ones (for example, healthcare coverage for psychological services, and/or affordable options, etc.)** is important to address.

- Critical priority
- High priority
- Undecided
- Low priority
- Not a priority

NavigatSystem_ClinR1 Eighty-seven percent (87.0%) of Canadian clinicians surveyed thought that **providing more accessible information and support to navigate the healthcare system and find appropriate services/personnel to assist and advocate for individuals with ADHD** is currently a *high priority* or *critical priority*. Considering this summary, please indicate the extent to which you feel **providing more accessible information and support to navigate the healthcare system and find appropriate services/personnel to assist and advocate for individuals with ADHD** is important to address.

- Critical priority
- High priority
- Undecided
- Low priority
- Not a priority

SelfAdvocate_ClinR1 Ninety-one percent (90.7%) of Canadian clinicians surveyed thought that **providing individuals with ADHD with the tools, information, and strategies to self-advocate** is currently a *high priority* or *critical priority*. Considering this summary, please indicate the extent to which you feel **providing individuals with ADHD with the tools, information, and strategies to self-advocate** is important to address.

- Critical priority
- High priority
- Undecided
- Low priority
- Not a priority

SocialSupport_ClinR1 Seventy-one percent (71.3%) of Canadian clinicians surveyed thought that **increasing the availability of social support networks for individuals, couples, and families with ADHD** is currently a *high priority* or *critical priority*. Considering this summary, please indicate the extent to which you feel **increasing the availability of social support networks for individuals, couples, and families with ADHD** is important to address.

- Critical priority
- High priority
- Undecided
- Low priority
- Not a priority

RuralAccess_ClinR1 Eighty-six percent (86.1%) of Canadian clinicians surveyed thought that **providing access to resources and services to smaller and/or rural communities** is currently a *high priority* or *critical priority*.

Considering this summary, please indicate the extent to which you feel **providing access to resources and services to smaller and/or rural communities** is important to address.

- Critical priority
- High priority
- Undecided
- Low priority
- Not a priority

TrainAllHCP_ClinR1 Ninety-four percent (94.4%) of Canadian clinicians surveyed thought that **increasing knowledge and training about ADHD and associated stigmas among all healthcare and mental health professionals (for example, family doctors, nurse practitioners, pharmacists, psychologists, counsellors, etc.)** is currently a *high priority* or *critical priority*. Considering this summary, please indicate the extent to which you feel **increasing knowledge and training about ADHD and associated stigmas among all healthcare and mental health professionals (for example, family doctors, nurse practitioners, pharmacists, psychologists, counsellors, etc.)**  is important to address.

- Critical priority
- High priority
- Undecided
- Low priority
- Not a priority

Barriers_ClinR1 Ninety-four percent (93.5%) of Canadian clinicians surveyed thought that **identifying delays/barriers to assessment and treatment, and the impacts they may have on different systems**is currently a *high priority* or *critical priority*. Considering this summary, please indicate the extent to which you feel **identifying delays/barriers to assessment and treatment, and the impacts they may have on different systems** is important to address.

- Critical priority
- High priority
- Undecided
- Low priority
- Not a priority

OptimizeAx_ClinR1 Eighty-eight percent (88.0%) of Canadian clinicians surveyed thought that **optimizing the assessment process through the use of validated tools to improve early diagnosis and diagnostic accuracy, and reduce misdiagnosis** is currently a *high priority* or *critical priority*. Considering this summary, please indicate the extent to which you feel **optimizing the assessment process through the use of validated tools to improve early diagnosis and diagnostic accuracy, and reduce misdiagnosis** is important to address.

- Critical priority
- High priority
- Undecided
- Low priority
- Not a priority

Title_SchoolWork ***ISSUES RELATED TO ADHD AWARENESS IN SCHOOLS & WORKPLACES***

SchoolSupport_ClinR1 Seventy-eight percent (77.8%) of Canadian clinicians surveyed thought that **increasing the availability of adapted supports within the school system (for example, report cards specific to students with ADHD, ADHD-friendly learning and aftercare programs, etc.)** is currently a *high priority* or *critical priority*. Considering this summary, please indicate the extent to which you feel **increasing the availability of adapted supports within the school system (for example, report cards specific to students with ADHD, ADHD-friendly learning and aftercare programs, etc.)**  is important to address.

- Critical priority
- High priority
- Undecided
- Low priority
- Not a priority

TrainEducator_ClinR1 Ninety-eight percent (98.1%) of Canadian clinicians surveyed thought that **educating personnel in the school systems on how to best support and teach individuals with ADHD**is currently a *high priority* or *critical priority*. Considering this summary, please indicate the extent to which you feel **educating personnel in the school systems on how to best support and teach individuals with ADHD** is important to address.

- Critical priority
- High priority
- Undecided
- Low priority
- Not a priority

BasicGenTrain_ClinR1 Seventy-five percent (75.0%) of Canadian clinicians surveyed thought that **providing basic general training in recognizing ADHD to all personnel who interact with youth in their line of work (for example, police, dentists, social workers, corrections officers, educators, etc.)** is currently a *high priority* or *critical priority*. Considering this summary, please indicate the extent to which you feel **providing basic general training in recognizing ADHD to all personnel who interact with youth in their line of work (for example, police, dentists, social workers, corrections officers, educators, etc.)**  is important to address.

- Critical priority
- High priority
- Undecided
- Low priority
- Not a priority

JobOpportun_ClinR1 Seventy-four percent (74.1%) of Canadian clinicians surveyed thought that **increasing job opportunities and workplace accommodations for all employees with ADHD** is currently a *high priority* or *critical priority*. Considering this summary, please indicate the extent to which you feel **increasing job opportunities and workplace accommodations for all employees with ADHD** is important to address.

- Critical priority
- High priority
- Undecided
- Low priority
- Not a priority

Title_Community ***ISSUES RELATED TO ADHD AWARENESS IN COMMUNITIES***

PositiveADHD_ClinR1 Sixty-four percent (63.7%) of Canadian clinicians surveyed thought that **redefining ADHD in a more positive/normative way as a facet of neurodiversity, to de-stigmatize and de-medicalize it (for example, by changing ADHD terminology to remove words like disorder, disability)** is currently a *high priority* or *critical priority*. Considering this summary, please indicate the extent to which you feel **redefining ADHD in a**

**more positive/normative way as a facet of neurodiversity, to de-stigmatize and de-medicalize it (for example, by changing ADHD terminology to remove words like disorder, disability)** is important to address.

- Critical priority
- High priority
- Undecided
- Low priority
- Not a priority

AtypicalADHD_ClinR1 Eighty-four percent (83.5%) of Canadian clinicians surveyed thought that **increasing public awareness of the different ways ADHD can present (for example, on a spectrum, with different symptom types, can be ‘masked’, etc.)** is currently a *high priority* or *critical priority*. Considering this summary, please indicate the extent to which you feel **increasing public awareness of the different ways ADHD can present (for example, on a spectrum, with different symptom types, can be ‘masked’, etc.)**  is important to address.

- Critical priority
- High priority
- Undecided
- Low priority
- Not a priority

TreatADHD_ClinR1 Seventy-eight percent (78.0%) of Canadian clinicians surveyed thought that **increasing awareness among the general public about treated vs. untreated ADHD and its implications among the general public (e.g., through awareness campaigns, school presentations and healthcare presentations)** is currently a *high priority* or *critical priority*. Considering this summary, please indicate the extent to which you feel **increasing awareness among the general public about treated vs. untreated ADHD and its implications among the general public (e.g., through awareness campaigns, school presentations and healthcare presentations)** is important to address.

- Critical priority
- High priority
- Undecided
- Low priority
- Not a priority

AttentionCheck Enter "123" in the box below.

________________________________________________________________

| Page Break |  |
| --- | --- |

Title_SpecificGroups ***ISSUES RELATED TO ADHD RESEARCH IN SPECIFIC GROUPS***

GirlsWomen_ClinR1 Ninety-four percent (93.5%) of Canadian clinicians surveyed thought that **increasing general awareness of ADHD and its impacts in girls and women (for example, among healthcare providers, across the lifespan, education, workplace, etc.)** is currently a *high priority* or *critical priority*. Considering this summary, please indicate the extent to which you feel **increasing general awareness of ADHD and its impacts in girls and women (for example, among healthcare providers, across the lifespan, education, workplace, etc.)**  is important to address.

- Critical priority
- High priority
- Undecided
- Low priority
- Not a priority

Hormones_ClinR1 Eighty-four percent (84.1%) of Canadian clinicians surveyed thought that **research on the impact of hormones (for example, hormonal fluctuations, hormone replacement therapy or contraceptives, etc.) on ADHD symptoms, and their interactions with ADHD medications** is currently a *high priority* or *critical priority*. Considering this summary, please indicate the extent to which you feel **research on the impact of hormones (for example, hormonal fluctuations, hormone replacement therapy or contraceptives, etc.) on ADHD symptoms, and their interactions with ADHD medications** is important to address.

- Critical priority
- High priority
- Undecided
- Low priority
- Not a priority

ImpactMeds_ClinR1 Eighty-one percent (81.3%) of Canadian clinicians surveyed thought that **research on the impact of ADHD medications on hormonal fluctuations and reproductive health** is currently a *high priority* or *critical priority*. Considering this summary, please indicate the extent to which you feel **research on the impact of ADHD medications on hormonal fluctuations and reproductive health** is important to address.

- Critical priority
- High priority
- Undecided
- Low priority
- Not a priority

MidlifeADHD_ClinR1 Eighty-one percent (81.3%) of Canadian clinicians surveyed thought that **research on recognizing and diagnosing ADHD in mid-life (ages 35-50)** is currently a *high priority* or *critical priority*. Considering this summary, please indicate the extent to which you feel **research on recognizing and diagnosing ADHD in mid-life (ages 35-50)** is important to address.

- Critical priority
- High priority
- Undecided
- Low priority
- Not a priority

MinorityADHD_ClinR1 Eighty-four percent (84.1%) of Canadian clinicians surveyed thought that **expanding our understanding of ADHD in under-served or marginalized populations (for example, ethnic minorities, queer and gender-diverse communities, and Indigenous groups)** is currently a *high priority* or *critical priority*. Considering this summary, please indicate the extent to which you feel **expanding our understanding of ADHD in under-served or marginalized populations (for example, ethnic minorities, queer and gender-diverse communities, and Indigenous groups)** is important to address.

- Critical priority
- High priority
- Undecided
- Low priority
- Not a priority

ComorbidExp_ClinR1 Seventy percent (70.1%) of Canadian clinicians surveyed thought that **research to understand the prevalence and unique experiences of people with ADHD and other mental health challenges** is currently a *high priority* or *critical priority*. Considering this summary, please indicate the extent to which you feel **research to understand the prevalence and unique experiences of people with ADHD and other mental health challenges** is important to address.

- Critical priority
- High priority
- Undecided
- Low priority
- Not a priority

Title_ImpactSymptom ***ISSUES RELATED TO MEASURING THE SYMPTOMS & IMPACTS OF ADHD***

Intergen_ClinR1 Seventy-eight percent (77.7%) of Canadian clinicians surveyed thought that **increasing knowledge and awareness about the impact of inter-generational ADHD (many generations of ADHD within a family)** is currently a *high priority* or *critical priority*. Considering this summary, please indicate the extent to which you feel **increasing knowledge and awareness about the impact of inter-generational ADHD (many generations of ADHD within a family)** is important to address.

- Critical priority
- High priority
- Undecided
- Low priority
- Not a priority

HealthCondt_ClinR1 Eighty-three percent (82.5%) of Canadian clinicians surveyed thought that **research to expand our knowledge of ADHD and co-occurring health-related conditions (for example, related to sleep, eating, oral health, personal hygiene, etc.)** is currently a *high priority* or *critical priority*. Considering this summary, please indicate the extent to which you feel **research to expand our knowledge of ADHD and co-occurring health-related conditions**

**(for example, related to sleep, eating, oral health, personal hygiene, etc.)**  is important to address.

- Critical priority
- High priority
- Undecided
- Low priority
- Not a priority

Socioemotion_ClinR1 Eighty-eight percent (88.3%) of Canadian clinicians surveyed thought that **research on socio-emotional functioning in ADHD (for example, self-esteem issues, ability to regulate emotions, etc.) and its impact on relationships** is currently a *high priority* or *critical priority*. Considering this summary, please indicate the extent to which you feel **research on socio-emotional functioning in ADHD (for example, self-esteem issues, ability to regulate emotions, etc.) and its impact on relationships** is important to address.

- Critical priority
- High priority
- Undecided
- Low priority
- Not a priority

Title_Treatments ***ISSUES RELATED TO TREATMENTS FOR ADHD***

HolisticTx_ClinR1 Seventy-nine percent (78.6%) of Canadian clinicians surveyed thought that **providing access to holistic treatment options supported by multi-disciplinary teams (for example, medication, nutrition, occupational therapy, etc.) embedded within different systems, including workplace and educational settings** is currently a *high priority* or *critical priority*. Considering this summary, please indicate the extent to which you feel **providing access to holistic treatment options supported by multi-disciplinary teams (for example, medication, nutrition, occupational therapy, etc.) embedded within different systems, including workplace and educational settings** is important to address.

- Critical priority
- High priority
- Undecided
- Low priority
- Not a priority

ShortLong_ClinR1 Eighty-four percent (83.5%) of Canadian clinicians surveyed thought that **researching how different treatments affect different individuals in the short- and long-term, using a person-centered approach to tailor treatments to all ADHD individuals (including those with co-occurring challenges like mental health concerns, intellectual disabilities, autism, etc.)** is currently a *high priority* or *critical priority*. Considering this summary, please indicate the extent to which you feel **researching how different treatments affect different individuals in the short- and long-term, using a person-centered approach to tailor treatments to all ADHD individuals (including those with co-occurring challenges like mental health concerns, intellectual disabilities, autism, etc.)**  is important to address.

- Critical priority
- High priority
- Undecided
- Low priority
- Not a priority

AlternativeTx_ClinR1 Seventy-two percent (71.8%) of Canadian clinicians surveyed thought that **research on optimizing existing non-drug treatments (for example, meditation and mindfulness, psychotherapy, physical activity, acupuncture, etc.)** is currently a *high priority* or *critical priority*. Considering this summary, please indicate the extent to which you feel **research on optimizing existing non-drug treatments (for example, meditation and mindfulness, psychotherapy, physical activity, acupuncture, etc.)**  is important to address.

- Critical priority
- High priority
- Undecided
- Low priority
- Not a priority

TxManagement_ClinR1 Eighty-five percent (85.4%) of Canadian clinicians surveyed thought that **educating individuals with ADHD and their loved ones about medication management, different medications available, and best treatment options** is currently a *high priority* or *critical priority*. Considering this summary, please indicate the extent to which you feel **educating individuals with ADHD and their loved ones about medication management, different medications available, and best treatment options** is important to address.

- Critical priority
- High priority
- Undecided
- Low priority
- Not a priority

Addictions_ClinR1 Ninety percent (90.3%) of Canadian clinicians surveyed thought that **research on best treatments for addictions within the context of ADHD, including addictions to substances, gaming, gambling, and screens** is currently a *high priority* or *critical priority*. Considering this summary, please indicate the extent to which you feel **research on best treatments for addictions within the context of ADHD, including addictions to substances, gaming, gambling, and screens** is important to address.

- Critical priority
- High priority
- Undecided
- Low priority
- Not a priority

Title_General ***GENERAL RESEARCH DIRECTIONS***

ADHDCauses_ClinR1 Seventy-one percent (70.9%) of Canadian clinicians surveyed thought that **continuing to work towards a better understanding of the causes of ADHD (for example, genetics, heritability, neurological mechanisms, risk factors, etc.)** is currently a *high priority* or *critical priority*. Considering this summary, please indicate the extent to which you feel **continuing to work towards a better understanding of the causes of ADHD (for example, genetics, heritability, neurological mechanisms, risk factors, etc.)**  is important to address.

- Critical priority
- High priority
- Undecided
- Low priority
- Not a priority

Strengths_ClinR1 Sixty-six percent (66.0%) of Canadian clinicians surveyed thought that **encouraging positive-directed research to better understand the unique strengths of those with ADHD** is currently a *high priority* or *critical priority*. Considering this summary, please indicate the extent to which you feel **encouraging positive-directed research to better understand the unique strengths of those with ADHD**  is important to address.

- Critical priority
- High priority
- Undecided
- Low priority
- Not a priority

LivedExp_ClinR1 Eighty-three percent (82.5%) of Canadian clinicians surveyed thought that **including people with lived experience in the process of research about ADHD** is currently a *high priority* or *critical priority*. Considering this summary, please indicate the extent to which you feel **including people with lived experience in the process of research about ADHD** is important to address.

- Critical priority
- High priority
- Undecided
- Low priority
- Not a priority

Stigma_ClinR1 Seventy percent (69.9%) of Canadian clinicians surveyed thought that **targeted research examining the stigmatization of ADHD (for example, feelings of self-stigma, parental stigma regarding their children, stigmatization in schools or classrooms)** is currently a *high priority* or *critical priority*. Considering this summary, please indicate the extent to which you feel **targeted research examining the stigmatization of ADHD (for example, feelings of self-stigma, parental stigma regarding their children, stigmatization in schools or classrooms)**  is important to address.

- Critical priority
- High priority
- Undecided
- Low priority
- Not a priority

Government_ClinR1 Eighty-nine percent (89.3%) of Canadian clinicians surveyed thought that **increasing understanding of ADHD as a condition warranting recognition by government and educational systems** is currently a *high priority* or *critical priority*. Considering this summary, please indicate the extent to which you feel **increasing understanding of ADHD as a condition warranting recognition by government and educational systems** is important to address.

- Critical priority
- High priority
- Undecided
- Low priority
- Not a priority

You have reached the end of the **clinician**survey

Giftcard Draw Please enter your email address below, if you wish to be entered in a draw to win one of ten $50 gift cards.

________________________________________________________________

**Thank you for your responses.**

If you have any additional feedback, please enter it here.

________________________________________________________________

FutureParticipation
If you are interested in hearing about ADHD-related studies in future, please enter your email address in the box below. Please note that you are under no obligation to participate in any future studies.

________________________________________________________________

# Survey Round 3. French version

Unravelling the Puzzles in ADHD: A Delphi Study (Wave 3)

Start of Block: CONSENT

Consent Form
**Merci d'avoir participé à notre étude de recherche, « Démêler les énigmes du TDAH avec la méthode Delphi : défis et opportunités de recherche à travers le Canada. »** Plus tôt cette année, vous avez répondu à un court sondage qui sollicitait votre perspective sur des sujets et des thèmes qui devraient être priorisés dans la recherche canadienne sur le trouble déficitaire de l'attention/hyperactivité (TDAH), dont le but ultime est d'atteindre un consensus général sur les priorités importantes liées au TDAH. Aujourd'hui, nous vous contactons pour répondre à la dernière partie de l'étude. Ce sondage devrait vous prendre 10 à 15 minutes à compléter, et une fois terminé vous aurez la chance de gagner l'une de dix cartes-cadeaux de 50 $ dans un tirage. Vous avez déjà consenti à participer à cette étude; si vous souhaitez revoir le formulaire de consentement, veuillez cliquer ici.  Merci pour votre contribution très précieuse à ce projet important! Sincèrement,  L'équipe d'étude CAPS ***C****anadian* ***A****DHD* ***P****riorities* ***S****urvey* Cette étude a été approuvée par le comité d'éthique de la recherche (REB22-0011). Votre participation est entièrement volontaire et confidentielle. **Chercheure:** XX **Co-chercheures :** XX. **Questions/Inquiétudes** Si vous avez des questions ou voulez obtenir des informations supplémentaires concernant l'étude et/ou votre participation, veuillez contacter: XX.

| Page Break |  |
| --- | --- |

END of Block: CONSENT

Start of Block: CLINICIANS_PRIORITIES

Text_DemoIntro **Suivant, nous allons vous poser des questions sur votre rôle principal vis-à-vis du TDAH et votre profil démographique.**

YearsExp_ClinR1 Combien d’années d’expérience avez-vous en pratique clinique?

- < 5 ans
- 5-10 ans
- 11-20 ans
- > 20 ans

Specialty_ClinR1 Où avez-vous complété votre spécialité clinique?

- J'ai obtenu mon diplôme de spécialité **au** **Canada**
- J'ai obtenu mon diplôme de ma spécialité à **l'extérieur du Canada**

PractSetting_ClinR1 Dans quel contexte exercez-vous actuellement?

- J'exerce en milieu **urbain**
- J'exerce en milieu **rural**

AttentionCheck Veuillez sélectionner « tout à fait d'accord » pour cette question.

- Tout à fait d'accord
- D'accord
- Ni d'accord, ni pas d'accord
- Pas d'accord
- Pas de tout d'accord

Service_ClinR1 Quel type de services fournissez-vous?

- Assuré uniquement
- Non assuré/privé uniquement
- Assurés et non assurés/privés

Assess_ClinR1 Quel type d'évaluations proposez-vous?

- En personne seulement
- Virtuel uniquement
- En personne et virtuellement

Background_ClinR1 Veuillez préciser l'origine raciale ou ethnique de votre famille. (Sélectionnez tout ce qui s'y rapporte)

- Caucasien/Européen/Blanc
- Noir/Africain
- Latino-américain/Hispanique (par exemple mexicain, chilien, etc.)
- Moyen-Orient/Arabe/Asie occidentale (par exemple égyptien, koweïtien, iranien, etc.)
- Asiatique du Sud/Inde orientale (par exemple Pakistanais, Sri Lankais, etc.
- Asiatique (par exemple chinois, coréen, japonais, etc.)
- Philippin/Insulaire du Pacifique
- Premières Nations/Autochtones
- Métis
- Inuit/Inuk
- Je préfère ne pas le dire
- Autre (Veuillez préciser): __________________________________________________

EducLevel_ClinR1 Quel est votre niveau d’études le plus élevé?

- Moins qu'un diplôme d'études secondaires
- Quelques études secondaires, pas de diplôme
- Diplôme d'études secondaires, diplôme ou équivalent
- Quelques crédits universitaires, pas de diplôme
- Formation commerciale/technique/professionnelle
- Certificat/Diplôme Professionnel
- Licence
- Une maîtrise
- Doctorat

AnnualIncome_ClinR1 Quelle est la tranche de revenus annuels de votre famille?

- Moins de 25 000 $
- Entre 25 001 $ et 50 000 $
- Entre 50 001 $ et 75 000 $
- Entre 75 001 $ et 100 000 $
- Entre 100 001 $ et 150 000 $
- Entre 150 001 $ et 200 000 $
- Plus de 200 001 $

Age_ClinR1 Quel est votre âge actuel?

________________________________________________________________

JobStatus_ClinR1 Quelle est votre situation professionnelle actuelle?

- Employé à temps complet
- Employé à temps partiel
- Travailleur indépendant
- Sans emploi
- Étudiant
- À la retraite
- Autre: __________________________________________________

Gender_ClinR1 À quel genre vous identifiez-vous?

- Homme
- Femme
- Autre
- Je préfère ne pas le dire

ProvReside_ClinR1 Dans quelle province canadienne résidez-vous?

- Alberta
- Colombie-Britannique
- Île-du-Prince-Édouard
- Manitoba
- Nouveau-Brunswick
- Nouvelle-Écosse
- Nunavut
- Ontario
- Saskatchewan
- Terre-Neuve et Labrador
- Territoires du Nord-Ouest
- Québec
- Yukon
- J'habite à l'extérieur du Canada

ClinicianPrompt Suite au premier sondage réalisé plus tôt cette année, nous avons colligé les opinions des **cliniciens** canadiens, incluant les vôtres, sur les types de thèmes et de problèmes liés au TDAH qui devraient être prioritaires actuellement.  Veuillez répondre à la prochaine série de questions en pensant à votre rôle, vos intérêts et vos perspectives en tant que **clinicien(ne)** dans le domaine du TDAH.

Title_AccessToCare ***ENJEUX LIÉS À LA QUALITÉ ET À L'ACCÈS AUX SOINS***

FundedService_ClinR1 Quatre-vingt-seize pourcent (96,3 %) des cliniciens canadiens sondés pensaient que de **fournir un accès à des services financés aux personnes avec un TDAH et à leurs proches (par exemple, une couverture médicale pour les services psychologiques et/ou des options abordables, etc.)** est actuellement une *priorité élevée* ou *critique*. Considérant ce résumé, veuillez indiquer dans quelle mesure vous pensez qu'il est important de **fournir un accès à des services financés aux personnes avec un TDAH et à leurs proches (par exemple, une couverture médicale pour les services psychologiques et/ou des options abordables, etc.)**.

- Priorité critique
- Priorité élevée
- Indécis(e)
- Priorité basse
- Pas une priorité

NavigatSystem_ClinR1 Quatre-vingt-sept pourcent (87,0 %) des cliniciens canadiens sondés pensaient que de **fournir des informations et un soutien plus accessibles pour naviguer dans le système de santé et trouver les services et le personnel appropriés au TDAH** est actuellement une *priorité élevée* ou *critique*. Considérant ce résumé, veuillez indiquer dans quelle mesure vous pensez qu'il est important de **fournir des informations et un soutien plus accessibles pour naviguer dans le système de santé et trouver les services et le personnel appropriés au TDAH**.

- Priorité critique
- Priorité élevée
- Indécis(e)
- Priorité basse
- Pas une priorité

SelfAdvocate_ClinR1 Quatre-vingt-onze pourcent (90,7 %) des cliniciens canadiens sondés pensaient que de **fournir aux personnes avec un TDAH les outils, les informations et les stratégies nécessaires pour renforcer leur autonomie sociale** est actuellement une *priorité élevée* ou *critique*. Considérant ce résumé, veuillez indiquer dans quelle mesure vous pensez qu'il est important de **fournir aux personnes avec un TDAH les outils, les informations et les stratégies nécessaires pour renforcer leur autonomie sociale**.

- Priorité critique
- Priorité élevée
- Indécis(e)
- Priorité basse
- Pas une priorité

SocialSupport_ClinR1 Soixante-et-onze pourcent (71,3 %) des cliniciens canadiens sondés pensaient que **d’accroître la disponibilité des réseaux de soutien social pour les individus, les couples et les familles avec un TDAH sociale** est actuellement une *priorité élevée* ou *critique*. Considérant ce résumé, veuillez indiquer dans quelle mesure vous pensez qu'il est important **d’accroître la disponibilité des réseaux de soutien social pour les individus, les couples et les familles avec un TDAH**.

- Priorité critique
- Priorité élevée
- Indécis(e)
- Priorité basse
- Pas une priorité

RuralAccess_ClinR1 Quatre-vingt-six pourcent (86,1 %) des cliniciens canadiens sondés pensaient que de **fournir un accès aux ressources et aux services dans les communautés rurales** est actuellement une *priorité élevée* ou *critique*. Considérant ce résumé, veuillez indiquer dans quelle mesure vous pensez qu'il est important de **fournir un accès aux ressources et aux services dans les communautés rurales**.

- Priorité critique
- Priorité élevée
- Indécis(e)
- Priorité basse
- Pas une priorité

TrainAllHCP_ClinR1 Quatre-vingt-quatorze pourcent (94,4 %) des cliniciens canadiens sondés pensaient que **d’accroître les connaissances et la formation sur le TDAH et la stigmatisation associée chez tous les professionnels de la santé et de la santé mentale (par exemple, les médecins de famille, les infirmières, les pharmaciens, les psychologues, etc.)** est actuellement une *priorité élevée* ou *critique*. Considérant ce résumé, veuillez indiquer dans quelle mesure vous pensez qu'il est important **d’accroître les connaissances et la formation sur le TDAH et la stigmatisation associée chez tous les professionnels de la santé et de la santé mentale (par exemple, les médecins de famille, les infirmières, les pharmaciens, les psychologues, etc.)**.

- Priorité critique
- Priorité élevée
- Indécis(e)
- Priorité basse
- Pas une priorité

Barriers_ClinR1 Quatre-vingt-quatorze pourcent (93,5 %) des cliniciens canadiens sondés pensaient que **d’identifier les obstacles à l'évaluation et au traitement, et les impacts qu'ils peuvent avoir sur différents systèmes** est actuellement une *priorité élevée* ou *critique*. Considérant ce résumé, veuillez indiquer dans quelle mesure vous pensez qu'il est important **d’identifier les obstacles à l'évaluation et au traitement, et les impacts qu'ils peuvent avoir sur différents systèmes**.

- Priorité critique
- Priorité élevée
- Indécis(e)
- Priorité basse
- Pas une priorité

OptimizeAx_ClinR1 Quatre-vingt-huit pourcent (88,0 %) des cliniciens canadiens sondés pensaient que **d’optimiser le processus d'évaluation grâce à l'utilisation d'outils validés pour améliorer le diagnostic précoce et la précision du diagnostic, et réduire les erreurs diagnostiques** est actuellement une *priorité élevée* ou *critique*. Considérant ce résumé, veuillez indiquer dans quelle mesure vous pensez qu'il est important **d’optimiser le processus d'évaluation grâce à l'utilisation d'outils validés pour améliorer le diagnostic précoce et la précision du diagnostic, et réduire les erreurs diagnostiques**.

- Priorité critique
- Priorité élevée
- Indécis(e)
- Priorité basse
- Pas une priorité

_SchoolWork ***ENJEUX LIÉS À LA SENSIBILISATION AU TDAH DANS LES ÉCOLES ET LES LIEUX DE TRAVAIL***

SchoolSupport_ClinR1 Soixante-dix-huit pourcent (77,8 %) des cliniciens canadiens sondés pensaient que **d’accroître la disponibilité de soutiens adaptés au sein du système scolaire (par exemple, bulletins scolaires spécifiques aux élèves avec un TDAH, programmes d'apprentissage et de suivi adaptés au TDAH, etc.)** est actuellement une *priorité élevée* ou *critique*. Considérant ce résumé, veuillez indiquer dans quelle mesure vous pensez qu'il est important **d’accroître la disponibilité de soutiens adaptés au sein du système scolaire (par exemple, bulletins scolaires spécifiques aux élèves avec un TDAH, programmes d'apprentissage et de suivi adaptés au TDAH, etc.)**.

- Priorité critique
- Priorité élevée
- Indécis(e)
- Priorité basse
- Pas une priorité

TrainEducator_ClinR1 Quatre-vingt-dix-huit pourcent (98,1 %) des cliniciens canadiens sondés pensaient que de **sensibiliser le personnel scolaire sur les meilleures façons de soutenir et d'enseigner aux personnes avec un TDAH** est actuellement une *priorité élevée* ou *critique*. Considérant ce résumé, veuillez indiquer dans quelle mesure vous pensez qu'il est important de **sensibiliser le personnel scolaire sur les meilleures façons de soutenir et d'enseigner aux personnes avec un TDAH**.

- Priorité critique
- Priorité élevée
- Indécis(e)
- Priorité basse
- Pas une priorité

BasicGenTrain_ClinR1 Soixante-quinze pourcent (75,0 %) des cliniciens canadiens sondés pensaient que de **fournir une formation générale de base sur la reconnaissance du TDAH à tout le personnel qui interagit avec les jeunes dans leur domaine de travail (par exemple, la police, les dentistes, les travailleurs sociaux, les agents correctionnels, les éducateurs, etc.)** est actuellement une *priorité élevée* ou *critique*. Considérant ce résumé, veuillez indiquer dans quelle mesure vous pensez qu'il est important de **fournir une formation générale de base sur la reconnaissance du TDAH à tout le personnel qui interagit avec les jeunes dans leur domaine de travail (par exemple, la police, les dentistes, les travailleurs sociaux, les agents correctionnels, les éducateurs, etc.)**.

- Priorité critique
- Priorité élevée
- Indécis(e)
- Priorité basse
- Pas une priorité

JobOpportun_ClinR1 Soixante-quatorze pourcent (74,1 %) des cliniciens canadiens sondés pensaient que **d’accroître les possibilités d'emploi et les aménagements en milieu de travail pour tous les employés avec un TDAH** est actuellement une *priorité élevée* ou *critique*. Considérant ce résumé, veuillez indiquer dans quelle mesure vous pensez qu'il est important **d’accroître les possibilités d'emploi et les aménagements en milieu de travail pour tous les employés avec un TDAH**.

- Priorité critique
- Priorité élevée
- Indécis(e)
- Priorité basse
- Pas une priorité

Title_Community ***ENJEUX LIÉS À LA SENSIBILISATION AU TDAH DANS LES COMMUNAUTÉS***

PositiveADHD_ClinR1 Soixante-quatre pourcent (63,7 %) des cliniciens canadiens sondés pensaient que de **redéfinir le TDAH de manière plus positive/normative comme une facette de la neurodiversité, pour le déstigmatiser et le démédicaliser (par exemple, en remplaçant les mots comme trouble, handicap)** est actuellement une *priorité élevée* ou *critique*. Considérant ce résumé, veuillez indiquer dans quelle mesure vous pensez qu'il est important de **redéfinir le TDAH de manière plus positive/normative comme une facette de la neurodiversité, pour le déstigmatiser et le démédicaliser (par exemple, en remplaçant les mots comme trouble, handicap)**.

- Priorité critique
- Priorité élevée
- Indécis(e)
- Priorité basse
- Pas une priorité

AtypicalADHD_ClinR1 Quatre-vingt-quatre pourcent (83,5 %) des cliniciens canadiens sondés pensaient que **d’accroître la sensibilisation du public aux différentes façons dont le TDAH peut se présenter (par exemple, sur un spectre, avec différents types de symptômes, peut être "masqué", etc.)** est actuellement une *priorité élevée* ou *critique*. Considérant ce résumé, veuillez indiquer dans quelle mesure vous pensez qu'il est important **d'accroître la sensibilisation du public aux différentes façons dont le TDAH peut se présenter (par exemple, sur un spectre, avec différents types de symptômes, peut être "masqué", etc.)**.

- Priorité critique
- Priorité élevée
- Indécis(e)
- Priorité basse
- Pas une priorité

TreatADHD_ClinR1 Soixante-dix-huit pourcent (78,0 %) des cliniciens canadiens sondés pensaient que **d’accroître la sensibilisation du grand public sur le TDAH traité par rapport au TDAH non traité et ses implications (par exemple, par le biais de campagnes de sensibilisation, de présentations dans les écoles ou dans les milieux de santé)** est actuellement une *priorité élevée* ou *critique*. Considérant ce résumé, veuillez indiquer dans quelle mesure vous pensez qu'il est important **d'accroître la sensibilisation du grand public sur le TDAH traité par rapport au TDAH non traité et ses implications (par exemple, par le biais de campagnes de sensibilisation, de présentations dans les écoles ou dans les milieux de santé)**.

- Priorité critique
- Priorité élevée
- Indécis(e)
- Priorité basse
- Pas une priorité

AttentionCheck Entrez « 123 » dans la case ci-dessous.

________________________________________________________________

Title_SpecificGroups ***ENJEUX LIÉS À LA RECHERCHE SUR LE TDAH DANS DES GROUPES SPÉCIFIQUES***

GirlsWomen_ClinR1 Quatre-vingt-quatorze pourcent (93,5 %) des cliniciens canadiens sondés pensaient que **d’accroître la sensibilisation générale au TDAH et à ses impacts chez les filles et les femmes (par exemple, auprès des professionnels de la santé, dans les écoles et les lieux de travail, en adoptant une perspective de parcours de vie, etc.)** est actuellement une *priorité élevée* ou *critique*. Considérant ce résumé, veuillez indiquer dans quelle mesure vous pensez qu'il est important **d'accroître la sensibilisation générale au TDAH et à ses impacts chez les filles et les femmes (par exemple, auprès des professionnels de la santé, dans les écoles et les lieux de travail, en adoptant une perspective de parcours de vie, etc.)**.

- Priorité critique
- Priorité élevée
- Indécis(e)
- Priorité basse
- Pas une priorité

Hormones_ClinR1 Quatre-vingt-quatre pourcent (84,1 %) des cliniciens canadiens sondés pensaient que la **recherche sur l'impact des hormones (par exemple, les fluctuations hormonales, l'hormonothérapie substitutive ou les contraceptifs, etc.) sur les symptômes du TDAH, et leurs interactions avec les médicaments pour le TDAH** est actuellement une *priorité élevée* ou *critique*. Considérant ce résumé, veuillez indiquer dans quelle mesure vous estimez qu'il est important **d'aborder la recherche sur l'impact des hormones (par exemple, les fluctuations hormonales, l'hormonothérapie substitutive ou les contraceptifs, etc.) sur les symptômes du TDAH, et leurs interactions avec les médicaments pour le TDAH**.

- Priorité critique
- Priorité élevée
- Indécis(e)
- Priorité basse
- Pas une priorité

ImpactMeds_ClinR1 Quatre-vingt-un pourcent (81,3 %) des cliniciens canadiens sondés pensaient que la **recherche sur l'impact des médicaments pour le TDAH sur les fluctuations hormonales et la santé reproductive** est actuellement une *priorité élevée* ou *critique*. Considérant ce résumé, veuillez indiquer dans quelle mesure vous estimez qu'il est important **d'aborder la recherche sur l'impact des médicaments pour le TDAH sur les fluctuations hormonales et la santé reproductive**.

- Priorité critique
- Priorité élevée
- Indécis(e)
- Priorité basse
- Pas une priorité

MidlifeADHD_ClinR1 Quatre-vingt-un pourcent (81,3 %) des cliniciens canadiens sondés pensaient que la **recherche sur la reconnaissance et le diagnostic du TDAH dans la quarantaine (35-50 ans)** est actuellement une *priorité élevée* ou *critique*. Considérant ce résumé, veuillez indiquer dans quelle mesure vous pensez qu'il est important **d'aborder la recherche sur la reconnaissance et le diagnostic du TDAH dans la quarantaine (35-50 ans)**.

- Priorité critique
- Priorité élevée
- Indécis(e)
- Priorité basse
- Pas une priorité

MinorityADHD_ClinR1 Quatre-vingt-quatre pourcent (84,1 %) des cliniciens canadiens sondés pensaient que de **répandre notre compréhension du TDAH dans les populations mal desservies ou marginalisées (par exemple, les minorités ethniques, les communautés LGBTQ+, et les groupes autochtones)** est actuellement une *priorité élevée* ou *critique*. Considérant ce résumé, veuillez indiquer dans quelle mesure vous pensez qu'il est important de **répandre notre compréhension du TDAH dans les populations mal desservies ou marginalisées (par exemple, les minorités ethniques, les communautés LGBTQ+, et les groupes autochtones)**.

- Priorité critique
- Priorité élevée
- Indécis(e)
- Priorité basse
- Pas une priorité

ComorbidExp_ClinR1 Soixante-dix percent (70,1 %) des cliniciens canadiens sondés pensaient que **les études pour comprendre la prévalence et les expériences uniques des personnes vivant avec des troubles de santé mentale en plus du TDAH** est actuellement une *priorité élevée* ou *critique*. Considérant ce résumé, veuillez indiquer dans quelle mesure vous pensez qu'il est important **d'aborder la recherche pour comprendre la prévalence et les expériences uniques des personnes vivant avec des troubles de santé mentale en plus du TDAH**.

- Priorité critique
- Priorité élevée
- Indécis(e)
- Priorité basse
- Pas une priorité

Title_ImpactSymptom ***ENJEUX LIÉS AUX SYMPTÔMES ET AUX IMPACTS DU TDAH***

Intergen_ClinR1 Soixante-dix-huit pourcent (77,7 %) des cliniciens canadiens sondés pensaient que **d’accroître les connaissances et la sensibilisation aux impacts du TDAH intergénérationnel (plusieurs générations de TDAH au sein d'une famille)** est actuellement une *priorité élevée* ou *critique*. Considérant ce résumé, veuillez indiquer dans quelle mesure vous pensez qu'il est important **d'accroître les connaissances et la sensibilisation aux impacts du TDAH intergénérationnel (plusieurs générations de TDAH au sein d'une famille)**.

- Priorité critique
- Priorité élevée
- Indécis(e)
- Priorité basse
- Pas une priorité

HealthCondt_ClinR1 Quatre-vingt-trois pourcent (82,5 %) des cliniciens canadiens sondés pensaient que la **recherche pour élargir nos connaissances sur le TDAH et les problématiques concomitantes liées à la santé (par exemple, liées au sommeil, à l'alimentation, à la santé bucco-dentaire, à l'hygiène personnelle, etc.)** est actuellement une *priorité élevée* ou *critique*. Considérant ce résumé, veuillez indiquer dans quelle mesure vous pensez qu'il est important **d'aborder la recherche pour élargir nos connaissances sur le TDAH et les problématiques concomitantes liées à la santé (par exemple, liées au sommeil, à l'alimentation, à la santé bucco-dentaire, à l'hygiène personnelle, etc.)**.

- Priorité critique
- Priorité élevée
- Indécis(e)
- Priorité basse
- Pas une priorité

Socioemotion_ClinR1 Quatre-vingt-huit pourcent (88,3 %) des cliniciens canadiens sondés pensaient que la **recherche sur le fonctionnement socio-émotionnel dans le TDAH (par exemple, les problèmes d'estime de soi, la capacité à réguler ses émotions, etc.) et son impact sur les relations interpersonnelles** est actuellement une *priorité élevée* ou *critique*. Considérant ce résumé, veuillez indiquer dans quelle mesure vous pensez qu'il est important **d'aborder la recherche sur le fonctionnement socio-émotionnel dans le TDAH (par exemple, les problèmes d'estime de soi, la capacité à réguler ses émotions, etc.) et son impact sur les relations interpersonnelles**.

- Priorité critique
- Priorité élevée
- Indécis(e)
- Priorité basse
- Pas une priorité

Title_Treatments ***ENJEUX LIÉS AUX TRAITEMENTS POUR LE TDAH***

HolisticTx_ClinR1 Soixante-dix-neuf pourcent (78,6 %) des cliniciens canadiens sondés pensaient que de **fournir un accès à des options de traitement holistiques soutenues par des équipes multidisciplinaires (par exemple, médicaments, nutrition, ergothérapie, etc.) intégrées dans différents systèmes, y compris les milieux de travail et d'enseignement** est actuellement une *priorité élevée* ou *critique*. Considérant ce résumé, veuillez indiquer dans quelle mesure vous pensez qu'il est important de **fournir un accès à des options de traitement holistiques soutenues par des équipes multidisciplinaires (par exemple, médicaments, nutrition, ergothérapie, etc.) intégrées dans différents systèmes, y compris les milieux de travail et d'enseignement**.

- Priorité critique
- Priorité élevée
- Indécis(e)
- Priorité basse
- Pas une priorité

ShortLong_ClinR1 Quatre-vingt-quatre pourcent (83,5 %) des cliniciens canadiens sondés pensaient que la **recherche sur comment différents traitements affectent différentes personnes à court et à long terme, en utilisant une approche centrée sur la personne pour adapter les traitements à toutes les personnes avec un TDAH (y compris celles présentant des problèmes concomitants tels que des problèmes de santé mentale, l'autisme, etc.)** est actuellement une *priorité élevée* ou *critique*. Considérant ce résumé, veuillez indiquer dans quelle mesure vous pensez qu'il est important **d'aborder la recherche sur comment différents traitements affectent différentes personnes à court et à long terme, en utilisant une approche centrée sur la personne pour adapter les traitements à toutes les personnes avec un TDAH (y compris celles présentant des problèmes concomitants tels que des problèmes de santé mentale, l'autisme, etc.)**.

- Priorité critique
- Priorité élevée
- Indécis(e)
- Priorité basse
- Pas une priorité

AlternativeTx_ClinR1 Soixante-douze pourcent (71,8 %) des cliniciens canadiens sondés pensaient que la **recherche sur l'optimisation des traitements non-pharmacologiques existants (par exemple, la méditation et la pleine conscience, la psychothérapie, l'activité physique, l'acuponcture, etc.)** est actuellement une *priorité élevée* ou *critique*. Considérant ce résumé, veuillez indiquer dans quelle mesure vous pensez qu'il est important **d'aborder la recherche sur l'optimisation des traitements non-pharmacologiques existants (par exemple, la méditation et la pleine conscience, la psychothérapie, l'activité physique, l'acuponcture, etc.)**.

- Priorité critique
- Priorité élevée
- Indécis(e)
- Priorité basse
- Pas une priorité

TxManagement_ClinR1 Quatre-vingt-cinq pourcent (85,4 %) des cliniciens canadiens sondés pensaient que **d’éduquer les personnes avec un TDAH et leurs proches sur la gestion des médicaments, les différents médicaments disponibles, et les meilleures options de traitement** est actuellement une *priorité élevée* ou *critique*. Considérant ce résumé, veuillez indiquer dans quelle mesure vous pensez qu'il est important **d’éduquer les personnes avec un TDAH et leurs proches sur la gestion des médicaments, les différents médicaments disponibles, et les meilleures options de traitement**.

- Priorité critique
- Priorité élevée
- Indécis(e)
- Priorité basse
- Pas une priorité

Addictions_ClinR1 Quatre-vingt-dix pourcent (90,3 %) des cliniciens canadiens sondés pensaient que la **recherche sur les meilleurs traitements pour les dépendances dans le contexte du TDAH, y compris les dépendances aux substances, au jeu, et aux écrans** est actuellement une *priorité élevée* ou *critique*. Considérant ce résumé, veuillez indiquer dans quelle mesure vous pensez qu'il est important **d'aborder la recherche sur les meilleurs traitements pour les dépendances dans le contexte du TDAH, y compris les dépendances aux substances, au jeu, et aux écrans**.

- Priorité critique
- Priorité élevée
- Indécis(e)
- Priorité basse
- Pas une priorité

Title_General ***ORIENTATIONS GÉNÉRALES DE RECHERCHE***

ADHDCauses_ClinR1 Soixante-et-onze pourcent (70,9 %) des cliniciens canadiens sondés pensaient que de **continuer à travailler vers une meilleure compréhension des causes du TDAH (par exemple, la génétique, l'héritabilité, les mécanismes neurologiques, les facteurs de risque, etc.)** est actuellement une *priorité élevée* ou *critique*. Considérant ce résumé, veuillez indiquer dans quelle mesure vous pensez qu'il est important de **continuer à travailler vers une meilleure compréhension des causes du TDAH (par exemple, la génétique, l'héritabilité, les mécanismes neurologiques, les facteurs de risque, etc.)**.

- Priorité critique
- Priorité élevée
- Indécis(e)
- Priorité basse
- Pas une priorité

Strengths_ClinR1 Soixante-six pourcent (66,0 %) des cliniciens canadiens sondés pensaient que **d’encourager la recherche orientée positivement pour mieux comprendre les forces uniques des personnes avec un TDAH** est actuellement une *priorité élevée* ou *critique*. Considérant ce résumé, veuillez indiquer dans quelle mesure vous pensez qu'il est important **d’encourager la recherche orientée positivement pour mieux comprendre les forces uniques des personnes avec un TDAH**.

- Priorité critique
- Priorité élevée
- Indécis(e)
- Priorité basse
- Pas une priorité

LivedExp_ClinR1 Quatre-vingt-trois pourcent (82,5 %) des cliniciens canadiens sondés pensaient que **d’inclure des personnes ayant un vécu du TDAH dans le processus de recherche sur le TDAH** est actuellement une *priorité élevée* ou *critique*. Considérant ce résumé, veuillez indiquer dans quelle mesure vous pensez qu'il est important **d’inclure des personnes ayant un vécu du TDAH dans le processus de recherche sur le TDAH**.

- Priorité critique
- Priorité élevée
- Indécis(e)
- Priorité basse
- Pas une priorité

Stigma_ClinR1 Soixante-dix pourcent (69,9 %) des cliniciens canadiens sondés pensaient que **les études ciblées examinant la stigmatisation du TDAH (par exemple, l'auto-stigmatisation, la stigmatisation des parents à l'égard de leurs enfants, la stigmatisation dans les écoles)** est actuellement une *priorité élevée* ou *critique*. Considérant ce résumé, veuillez indiquer dans quelle mesure vous pensez qu'il est important **d'aborder les études ciblées examinant la stigmatisation du TDAH (par exemple, l'auto-stigmatisation, la stigmatisation des parents à l'égard de leurs enfants, la stigmatisation dans les écoles**.

- Priorité critique
- Priorité élevée
- Indécis(e)
- Priorité basse
- Pas une priorité

Government_ClinR1 Quatre-vingt-neuf pourcent (89,3 %) des cliniciens canadiens sondés pensaient que **d’accroître la compréhension du TDAH en tant que condition qui doit être reconnue par le gouvernement et les systèmes éducatifs** est actuellement une *priorité élevée* ou *critique*. Considérant ce résumé, veuillez indiquer dans quelle mesure vous pensez qu'il est important **d’accroître la compréhension du TDAH en tant que condition qui doit être reconnue par le gouvernement et les systèmes éducatifs**.

- Priorité critique
- Priorité élevée
- Indécis(e)
- Priorité basse
- Pas une priorité

Vous avez terminé le sondage à l'intention des **cliniciens**.

End of Block: CLINICIANS_PRIORITIES

Start of Block: CONTACT

Giftcard Draw S'il vous plaît veuillez indiquer votre adresse courriel pour participer au tirage d'une carte-cadeau d'une valeur de 50 $ (parmi dix).

________________________________________________________________

End of Block: CONTACT

Start of Block: EndBlock

ThankYou
**Merci pour vos réponses.**

Si vous avez des commentaires supplémentaires, veuillez les saisir ici.

________________________________________________________________

FutureParticipation

Si vous souhaitez recevoir des informations sur les études liées au TDAH à l'avenir, veuillez entrer votre adresse courriel dans la case ci-dessous. Veuillez noter que vous n'êtes pas obligé(e) de participer à d'études futures.

________________________________________________________________

End of Block: EndBlock
